# Nanocarrier imaging at single-cell resolution across entire mouse bodies with deep learning

**DOI:** 10.1038/s41587-024-02528-1

**Published:** 2025-01-14

**Authors:** Jie Luo, Muge Molbay, Ying Chen, Izabela Horvath, Karoline Kadletz, Benjamin Kick, Shan Zhao, Rami Al-Maskari, Inderjeet Singh, Mayar Ali, Harsharan Singh Bhatia, David-Paul Minde, Moritz Negwer, Luciano Hoeher, Gian Marco Calandra, Bernhard Groschup, Jinpeng Su, Ceren Kimna, Zhouyi Rong, Nikolas Galensowske, Mihail Ivilinov Todorov, Denise Jeridi, Tzu-Lun Ohn, Stefan Roth, Alba Simats, Vikramjeet Singh, Igor Khalin, Chenchen Pan, Bernardo A. Arús, Oliver T. Bruns, Reinhard Zeidler, Arthur Liesz, Ulrike Protzer, Nikolaus Plesnila, Siegfried Ussar, Farida Hellal, Johannes Paetzold, Markus Elsner, Hendrik Dietz, Ali Erturk

**Affiliations:** 1 Institute for Intelligent Biotechnologies (iBIO), Helmholtz Center Munich, Neuherberg, Germany; 2 Institute for Stroke and Dementia Research, Klinikum der Universität München, Ludwig-Maximilians University Munich, Munich, Germany; 3 Munich Cluster for Systems Neurology (SyNergy), Munich, Germany; 4 Deep Piction, Munich, Germany; 5 Munich Medical Research School (MMRS), Munich, Germany; 6 Pharmaceutical Technology and Biopharmaceutics, Department of Pharmacy, Ludwig-Maximilians-University Munich, Munich, Germany; 7 Faculty of Medicine, Ludwig-Maximilians University Munich, Munich, Germany; 8 TUM School of Computation, Information and Technology, Technical University of Munich, Munich, Germany; 9 Department of Biosciences, School of Natural Sciences, Technical University of Munich, Garching, Germany; 10 Munich Institute of Biomedical Engineering, Technical University of Munich, Garching, Germany; 11 Department of Quantitative Biomedicine, University of Zurich, Zurich, Switzerland; 12 ETH Zurich, Institute for Molecular Health Sciences, Zurich, Switzerland; 13 Research Unit Adipocytes & Metabolism (ADM), Helmholtz Diabetes Center, Helmholtz Zentrum München, Neuherberg, Germany; 14 German Center for Diabetes Research (DZD), Neuherberg, Germany; 15 Department of Medicine, Technische Universität München, Munich, Germany; 16 Graduate School of Neuroscience (GSN), Munich, Germany; 17 Institute of Computational Biology, Helmholtz Munich, Neuherberg, Germany; 18 Institute of Virology, Technical University of Munich / Helmholtz Munich, Munich, Germany; 19 German Center for Infection Research (DZIF), Munich partner site, Munich, Germany; 20 Normandie University, UNICAEN, INSERM UMR-S U1237, Physiopathology and Imaging of Neurological Disorders (PhIND), GIP Cyceron, Institute Blood and Brain @Caen-Normandie (BB@C), Caen, France; 21 Department of Functional Imaging in Surgical Oncology, National Center for Tumor Diseases (NCT/UCC), Dresden, Germany; 22 German Cancer Research Center (DKFZ), Heidelberg, Germany; 23 Medizinische Fakultät and University Hospital Carl Gustav Carus, Technische Universität Dresden, Dresden, Germany; 24 Helmholtz Zentrum Dresden-Rossendorf (HZDR), Dresden, Germany; 25 Helmholtz Pioneer Campus, Helmholtz Zentrum München, Neuherberg, Germany; 26 Helmholtz Zentrum München, German Research Center for Environmental Health, Institute of Structural Biology, Munich, Germany; 27 Department of Otorhinolaryngology, LMU University Hospital, LMU Munich, Munich, Germany; 28 Department of Computing, Imperial College London, London, UK; 29 School of Medicine, Koç University, İstanbul, Turkey

**Keywords:** Biotechnology, Fluorescence imaging

## Abstract

Efficient and accurate nanocarrier development for targeted drug delivery is hindered by a lack of methods to analyze its cell-level biodistribution across whole organisms. Here we present Single Cell Precision Nanocarrier Identification (SCP-Nano), an integrated experimental and deep learning pipeline to comprehensively quantify the targeting of nanocarriers throughout the whole mouse body at single-cell resolution. SCP-Nano reveals the tissue distribution patterns of lipid nanoparticles (LNPs) after different injection routes at doses as low as 0.0005 mg kg^−1^—far below the detection limits of conventional whole body imaging techniques. We demonstrate that intramuscularly injected LNPs carrying SARS-CoV-2 spike mRNA reach heart tissue, leading to proteome changes, suggesting immune activation and blood vessel damage. SCP-Nano generalizes to various types of nanocarriers, including liposomes, polyplexes, DNA origami and adeno-associated viruses (AAVs), revealing that an AAV2 variant transduces adipocytes throughout the body. SCP-Nano enables comprehensive three-dimensional mapping of nanocarrier distribution throughout mouse bodies with high sensitivity and should accelerate the development of precise and safe nanocarrier-based therapeutics.

## Abstract (web summary)

An integrated experimental and deep learning pipeline reveals cell-level targeting of nanocarriers in whole bodies.

## Main

Modern biomedical science offers a vast array of macromolecular drugs (for example, various RNA species, genome editing tools and protein drugs) with the potential to reverse disease-causing alterations in the human body^1,2^. However, a major hurdle in their clinical translation lies in delivering these large, charged molecules specifically to target cell populations while minimizing off-target effects.

Nanocarriers, such as lipid nanoparticles (LNPs)^3^, liposomes^4^, apolyplexes^5^ and viral vectors, such as adeno-associated viruses (AAVs)^6^, are among the most promising delivery solutions. They protect drug molecules, help to overcome biological barriers and can mediate organ and cell type targeting³. With over 30 US Food & Drug Administration/European Medicines Agency (FDA/EMA)-approved products and numerous clinical trials underway, these nanocarriers hold immense therapeutic potential, as exemplified by the success of the severe acute respiratory syndrome coronavirus 2 (SARS-CoV-2) vaccines. Emerging modalities such as DNA origami offer ease of production, modification and especially programmability^7–12^. To enhance stability and targeting, nanocarriers are often coated with polymers, such as polyethylene glycol (PEG), and functionalized with targeting moieties, such as antibodies. However, upon exposure to the in vivo environment, nanocarriers inevitably acquire a protein corona that can influence their biodistribution and cellular interactions^13^, complicating nanocarrier design and the prediction of target tissue.

A critical challenge across all nanocarrier strategies is maximizing specificity and efficiency for target tissues and cells while minimizing adverse and off-target effects. Existing methods for analyzing nanocarrier biodistribution within whole mouse bodies, such as positron emission tomography (PET), computed tomography (CT), magnetic resonance imaging (MRI) or in vivo optical imaging, lack the resolution to identify the millions of individual cells targeted by nanocarriers in three dimensions (3D) and often the sensitivity to work with the low doses employed in applications, such as preventive and therapeutic vaccines. Similarly, their ability to detect and analyze low-intensity off-target sites is limited^14–16^. Conversely, traditional histological approaches offer subcellular resolution and high sensitivity but rely on thin, pre-selected two-dimensional (2D) tissue sections, making them unsuitable for whole animal analysis^17,18^.

**Fig. 1:**
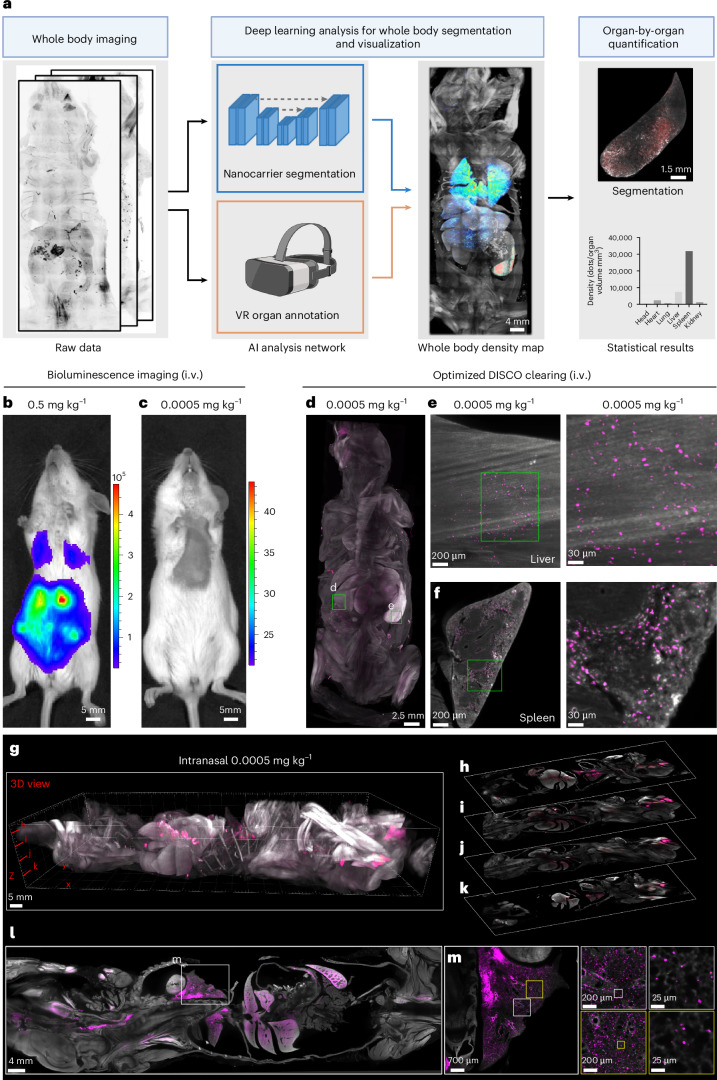
Optimized DISCO clearing for imaging nanocarriers at low doses. **a**, Scheme of SCP-Nano—a pipeline for mapping and quantifying the biodistribution of any fluorescently labeled nanocarrier throughout the entire mouse body with single-cell resolution and high sensitivity. **b**,**c**, Bioluminescence imaging (ventral) 6 h after intravenous injection of 0.5 mg kg^−1^ (**b**) and 0.0005 mg kg^−1^ (**c**) of luciferase mRNA-carrying LNPs. **d**–**f**, Whole body light sheet imaging of mice intravenously injected with 0.0005 mg kg^−1^ Alexa Fluor 647–labeled EGFP mRNA-carrying LNPs and cleared with our refined DISCO clearing methods. This approach enables the visualization of mRNA delivery throughout the entire mouse body, including the liver (**e**) and spleen (**f**), at cellular resolution. **g**–**m**, Visualization of whole mouse body LNPs after intranasal delivery of 0.0005 mg kg^−1^: maximum intensity projection (**g**) and single optical slice views (**h**–**l**); representative individual optical slices of the lung (**m**).

To address these limitations, we developed Single Cell Precision Nanocarrier Identification (SCP-Nano)—a pipeline for mapping and quantifying the biodistribution of any fluorescence-labeled nanocarrier throughout the entire mouse body with single-cell resolution and high sensitivity (Fig. 1a). SCP-Nano uses an advanced deep learning pipeline to analyze large-scale imaging data generated by a DISCO tissue clearing and light sheet microscopy method optimized for nanocarrier imaging. This approach enables precise quantification of nanomedicine delivery at the organ, tissue and single-cell level across whole mouse bodies. We demonstrate the utility of SCP-Nano for studying LNP-based mRNA delivery, quantifying biodistribution at doses as low as 0.0005 mg kg^−1^ (as commonly used in vaccines, which is 100–1,000 times lower than those typically used in conventional imaging studies for nanoparticles), uncovering application-route-dependent tissue tropism. Notably, we detected low-intensity off-target LNP accumulation in heart tissue after SARS-CoV-2 spike mRNA delivery, with subsequent proteomic analysis revealing changes in the expression of immune and vascular proteins, which might explain some of the reported clinical obervations^19–22^. Finally, we demonstrate the generalizability of SCP-Nano by applying it to liposomes, polyplexes, DNA origami and two adeno-associated virus (AAV) variants, identifying adipose tissue as a major target of the AAV2 variant Retro-AAV. Our artificial intelligence (AI)-based quantification pipeline substantially outperformed previously published approaches in terms of accuracy and scalability to millions of targeting events, and our integrated spatial proteomics analysis provides insights into the molecular basis and effects of tissue targeting. SCP-Nano should accelerate the development of precise and safe nanocarrier-based therapeutics.

## Results

### High-resolution, whole body biodistribution imaging

Conventional bioluminescence imaging, a common whole body imaging technique, has identified luciferase expression after LNP-based delivery of its mRNA at high injection doses (0.5 mg kg^−1^) with high contrast at the organ level (Fig. 1b). However, signal contrast drops drastically at low doses typically used, for example, for mRNA vaccines (0.0005 mg kg^−1^) (Fig. 1c and Supplementary Fig. 1).

To visualize LNP distribution with higher sensitivity and resolution, we generated LNPs based on the clinically approved MC3-ionizable lipid^23^ carrying EGFP mRNA—tagged with Alexa fluorescent dyes (Alexa 647 or Alexa 750).

We first optimized the DISCO whole mouse clearing technique to enable sensitive 3D imaging of clinical LNP doses at the single-cell level. We found that eliminating urea and sodium azide and reducing dichloromethane (DCM) incubation time were crucial for preserving the fluorescence signal of Alexa Fluor–tagged mRNAs throughout the mouse body^24–27^ (Supplementary Fig. 2 and Methods). Using this refined DISCO method, we imaged mice at a resolution of approximately 1–2 µm (lateral) and approximately 6 µm (axial), revealing extensive cellular targeting of the LNPs, especially in the liver and spleen, even at doses as low as 0.0005 mg kg^−1^ (Fig. 1d–f and Supplementary Fig. 3), achieving single-cell resolution across the body (Supplementary Fig. 4a–d). Dye conjugation to the mRNA did not affect LNP biodistribution, as the LNPs conjugated with two different fluorescent tags targeted the same regions equally (Supplementary Fig. 4e), and labeling of the lipid component with Alexa Fluor 647 yielded similar results (Supplementary Fig. 4f).

Next, we compared the biodistribution of LNPs administered intranasally or intramuscularly, routes under investigation in numerous clinical trials^28^, again revealing widespread cellular targeting throughout the body, particularly in the lung, liver and spleen (Fig. 1d–m, Supplementary Fig. 3 and Supplementary Video 1). Closer inspection highlighted thousands of targeted cells across these organs (Fig. 1m and Supplementary Fig. 3b,c).

To assess potential signal loss during clearing, we further validated our method using histology. After perfusion and fixation of the whole mouse, we generated histological slices of lymph nodes and liver and imaged the distribution of LNP-expressed EGFP proteins. Then, the same slices were cleared using our optimized DISCO protocol and imaged again. Both signal contrast and number of EGFP protein-positive structures were well preserved before and after clearing (Supplementary Fig. 5a). Furthermore, our clearing technique preserves nanoparticles both inside and outside cells, as seen using confocal microscopy after tissue clearing and whole body imaging (Supplementary Fig. 5b).

To show generalizability of our method, we also assessed the cell-level distribution of liposomes (based on the clinically approved Doxil formulation) and polyplexes (based on branched polyethyleneimine (PEI)) that deliver a COOH-modified Atto 647 or single-stranded DNA (ssDNA)–Alexa Fluor 647, respectively (Supplementary Fig. 6).

### AI-based cell-level quantification of LNP targeting

The optimized tissue clearing enables the visualization of LNP-targeted cells but requires unbiased and reliable quantification to compare whole body biodistribution under various conditions^29^. We found that existing methods, such as the filter-based Imaris software and the deep learning solution DeepMACT^30^, delivered suboptimal results (F1 scores < 0.50). Therefore, we developed a robust deep learning pipeline to detect and quantify tens of millions of targeted cells reliably in the different tissues of whole mice.

**Fig. 2:**
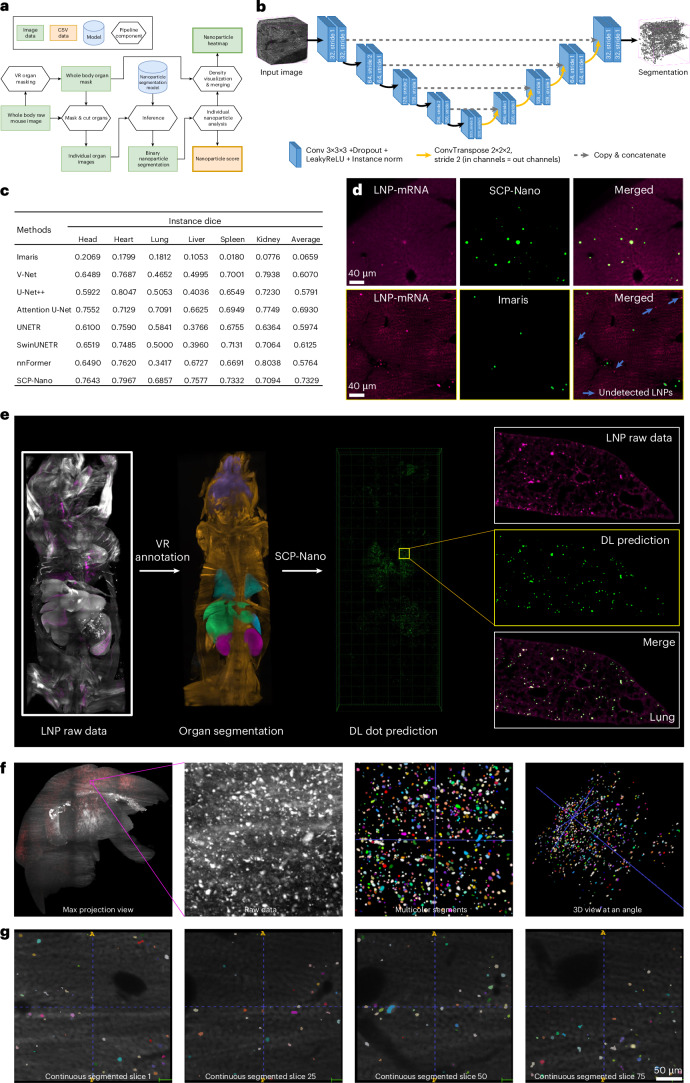
SCP-Nano—a deep-learning-based pipeline to segment and analyze all targeted cells. **a**, Flowchart of the SCP-Nano pipeline. **b**, Customized 3D U-Net architecture of the SCP-Nano deep learning segmentation model. **c**, Comparison of the F1 (instance Dice) scores of SCP-Nano segmentation model with other models. **d**, Comparison of Imaris and SCP-Nano prediction accuracy using liver images. **e**, Illustration of the entire nanoparticle prediction pipeline. After obtaining the whole body dataset via light sheet microscopy, we used VR glasses for organ annotation, followed by the application of our SCP-Nano analysis algorithm to quantify the LNP distribution in the whole body. Example images are from the lung. Compared to the ground truth data, our algorithm accurately detects all different sizes of delivery events in the lung. **f**, Raw data of LNP distribution in the liver and instance-separated multicolor segmentation obtained by SCP-Nano. Each color represents a separate delivery event as predicted by the model. **g**, Continuous segmented slice views demonstrate single-cell segmentation.

Our approach involves partitioning whole body imaging data into discrete units for deep learning analysis within the typical memory constraints imposed by the available computational resources (Fig. 2a and Supplementary Fig. 7). We created a training dataset using a virtual reality (VR)-based annotation method, previously proven superior to slice-based approaches^31^. This dataset included 31 3D patches (200 × 200 × 200 to 300 × 300 × 300 voxels) randomly selected from diverse tissues (head, heart, lungs, kidneys, liver, lymph nodes and spleen) after intramuscular and intravenous injection of LNPs. We manually split the data into training/validation and test sets to track the segmentation model’s performance across relevant organs. Performance was evaluated using the instance F1 score (or Dice coefficient)^30^.

We trained several deep learning models (VNet^32^, U-Net++^33^, Attention U-Net^34^, UNETR^35^, SwinUNETR^36^, nnFormer^36^ and 3D U=Net^37^) using established parameter selection, training protocols and five-fold cross-validation. Our highest-performing model employed a 3D U-Net architecture with six encoding and five decoding layers with a leaky rectified linear unit (ReLU) activation function (Fig. 2b). SCP-Nano achieved an average instance F1 score of 0.7329 on the independent test dataset, with organ-specific scores ranging from 0.6857 to 0.7967 (Fig. 2c,d). The segmentation performance was not affected by injection route (Supplementary Figs. 8 and 9).

We used the cc3d library^36^ to identify each segmented targeted cell/cluster instance and calculate its size and intensity contrast relative to the background. This allowed us to compute organ-level statistics and visualize nanocarrier density within organs or volumes of interest. Together with the refined DISCO imaging, this deep learning model formed our SCP-Nano pipeline to quantify LNP targeting and biodistribution throughout the whole mouse body organ by organ at the cell level (Fig. 2e,f and Supplementary Fig. 10). Notably, SCP-Nano could readily identify individual cells even in regions with high signal density, as the algorithm does not rely on single-value thresholding but, rather, makes its prediction based on factors such as particle shape and brightness value relative to its neighborhood to faithfully identify signal at different intensities (Supplementary Fig. 10).

### SCP-Nano reveals tropism of LNPs via different routes

**Fig. 3:**
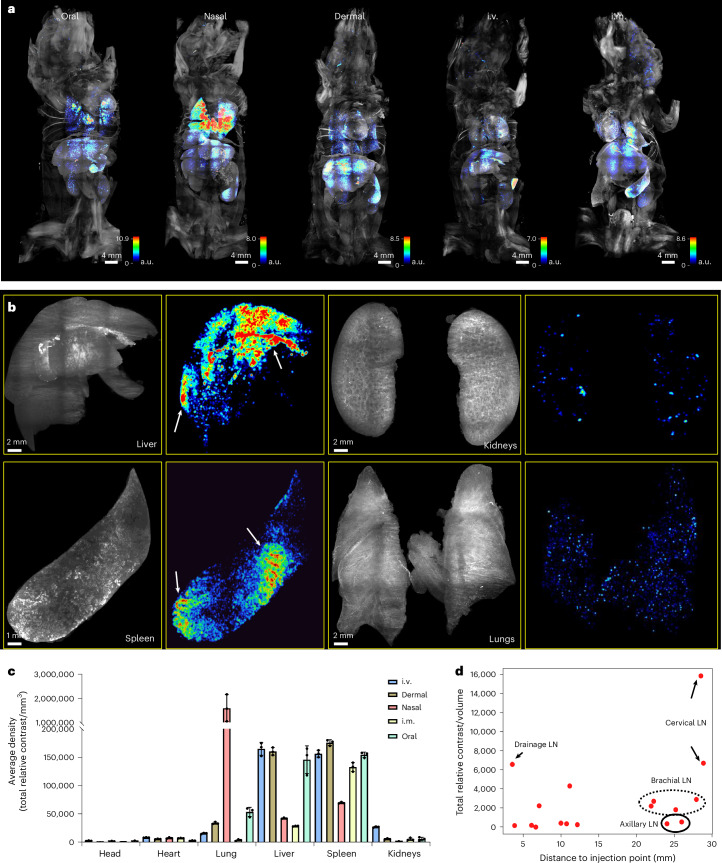
SCP-Nano reveals differences in LNP biodistribution based on different application routes. **a**, Density heatmaps of the distribution of LNP-delivered mRNA applied using different routes (0.0005 mg kg^−1^ in each case). **b**, Raw projection images (left) and density heatmaps of selected organs. Arrows point to intra-organ delivery hotspots. **c**, Organ-level quantification of mRNA delivery events across key organs for different application routes using the SCP-Nano deep learning algorithm (*n* = 3 mice per group, mean ± s.d.). **d**, Quantification of mRNA delivery events in lymph nodes of intramuscularly injected mice. i.m., intramuscular; i.v., intravenous; LN, lymph node. Source data

Next, we employed SCP-Nano to investigate how different administration routes affect the biodistribution of LNPs. LNPs carrying fluorescently tagged EGFP mRNA were delivered intramuscularly, intradermally, orally, intravenously and intranasally at a dose of 0.0005 mg kg^−1^, with analysis performed 6 h after injection (*n* = 3 mice per group). To facilitate qualitative comparisons, we generated delivery density maps of whole mouse bodies and observed substantial heterogeneity in delivery efficiency across and within individual organs (Fig. 3a). Notably, the resolution of SCP-Nano revealed localized hotspots within organs, such as in the liver and spleen (white arrows in Fig. 3b), that would be difficult to discern with conventional methods.

SCP-Nano yielded quantitative data on the distinct distribution patterns within organs based on the administration route (Fig. 3c). Intranasal administration led to the highest mRNA dose retention, primarily within the lungs. In contrast, as expected, most other routes predominantly targeted the liver and spleen (Fig. 3a–c).

The differences between the administration routes recommend them for different purposes. Nasal delivery mainly targets respiratory tissues, particularly the lungs. Intravenous and dermal delivery mainly target the liver and spleen, although we found a relative liver de-targeting and increased spleen targeting after intramuscular delivery, suggesting that this route may be beneficial for avoiding the liver when targeting immune organs (Fig. 3a,c).

Given the widespread use of LNPs for mRNA vaccine delivery, we examined their ability to transport mRNA to different lymph nodes after intramuscular (hindlimb) injection (Fig. 3d, Supplementary Fig. 11 and Supplementary Table 1). LNP-delivered mRNA was detected in all analyzed lymph nodes, with no clear correlation between distance from the injection site and mRNA levels. Notably, the directly draining lymph node and, to an even greater extent, the cervical lymph nodes near the neck received notably higher doses of mRNA than the other lymph nodes. This observation underscores the potential of LNPs to facilitate an immune response in lymph nodes, thereby enhancing vaccine effectiveness.

The deep learning model trained on LNP data was also able to segment and quantify the distribution of liposomes and polyplexes without retraining (Supplementary Fig. 12a,b).

Overall, these findings highlight the intricacies of nanocarrier biodistributions across and within organs, stressing the necessity of cellular-level analysis to understand distribution patterns fully.

### SCP-Nano reveals potential off-targeting effects

Having visualized LNP-targeted cells, we aimed to identify cells actively expressing LNP-delivered mRNAs. Previous studies indicated that not all LNP-targeted cells translate their RNA cargo^38^. To enable the simultaneous identification of both LNP-targeted cells and those expressing the encapsulated mRNA, we used an Alexa-tagged EGFP mRNA (for imaging LNP targeting) and a nanobody against EGFP protein (for visualizing mRNA translation) in the optimized DISCO clearing protocol.

**Fig. 4:**
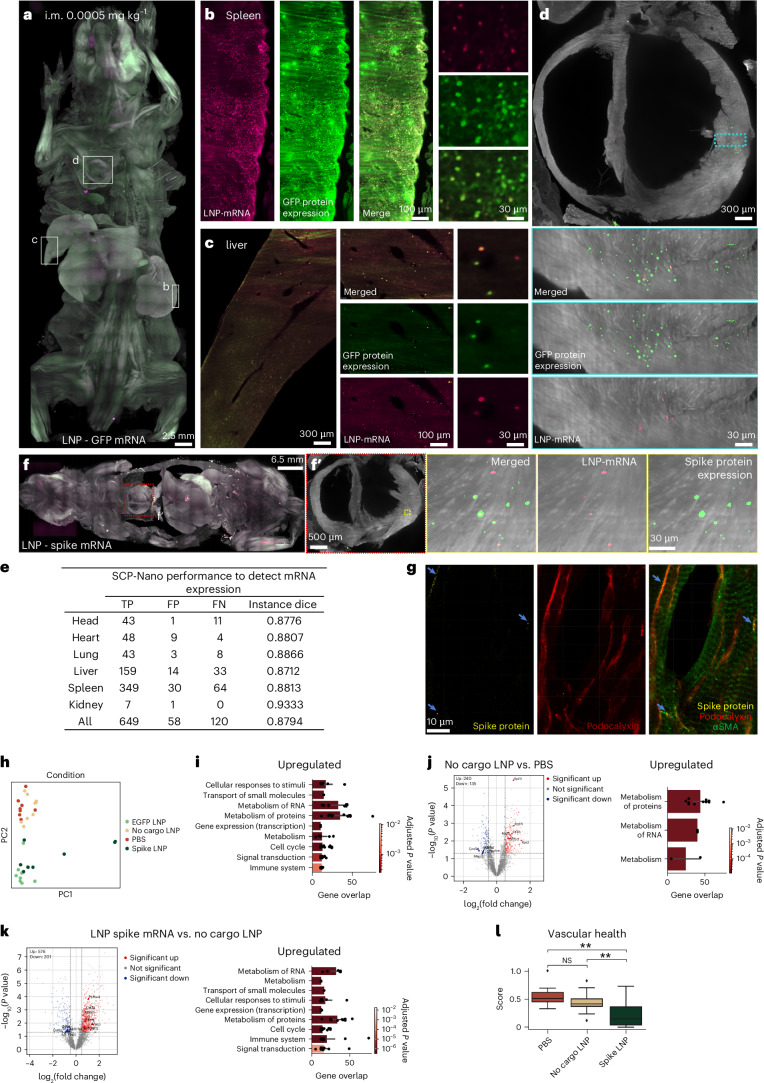
SCP-Nano reveals protein expression from LNP-delivered mRNA and LNP off-targeting. **a**. Whole body projection view of mRNA and EGFP protein expression 72 h after intramuscular injection of 0.0005 mg kg^−1^ EGFP mRNA-LNPs. **b**–**d**, Detailed views of the spleen (**b**), liver (**c**) and heart (**d**). **e**, Quantitative evaluation of the SCP-Nano segmentation model (fine-tuned on EGFP data) for detecting protein expression (FN, false negatives; FP, false positives; TP, true positives, compared to manual annotation). **f**. Body-wide distribution of SARS-CoV-2 spike S1 protein derived from LNP-delivered mRNA administered intramuscularly at 72 h after injection (**f**). Spike proteins were detected in the heart (**f′**). **g**, Confocal images of heart tissue sections stained for endothelial cells in capillaries using podocalyxin antibody (red), arteries using αSMA antibody (green) and spike S1 protein using a spike nanobody (yellow). **h**, PCA of mass-spectrometry-based proteomics data of different groups: spike LNP, EGFP LNP, no-cargo LNP and PBS. **i**, Top-level pathways in Reactome database differentially expressed between the two control groups (no-cargo LNP/PBS) and the combined spike LNP and EGFP LNP groups (*n* = 9, mean ± s.d.; one-way ANOVA). **j**,**k**, Same analysis for proteins upregulated in no-cargo LNP in comparison to the PBS (**j**) and in the spike mRNA in comparison to no-cargo LNP (**k**) (*n* = 9, mean ± s.d.; one-way ANOVA). **l**, Analysis of vascular health using typical protein markers (Supplementary Table 3) for the three different groups. NS *P* > 0.05, **P < 0.01 (*n* = 9; one-way ANOVA). i.m., intramuscular; PC, principal component; NS, not significant.

We applied SCP-Nano to visualize EGFP-expressing cells throughout mouse bodies 72 h after intramuscular injection with LNPs carrying EGFP mRNA, using both a low dose (0.0005 mg kg^−1^; Fig. 4a–d) and a high dose (0.5 mg kg^−1^; Supplementary Fig. 12c–f). EGFP expressions were detectable at both doses, whereas most of the Alexa-mRNA signal was lost due to RNA degradation at 72 h. We refined the SCP-Nano algorithms by training with an annotated EGFP dataset (Methods) to quantify EGFP expression, achieving an average F1 score of 0.81 across all organs (Fig. 4e).

The sensitivity of our pipeline allows us to detect low-intensity signals in potential off-target tissues. Here, we, for example, observed sparse but distinct LNP targeting and EGFP mRNA expression in mouse hearts (Fig. 4d and Supplementary Video 2). Given that MC3-based LNPs are used in RNA therapeutics and drug development in mice, non-human primates and humans^23,38–41^ and that there are reports of cardiac complications after vaccination using another LNP formulation^42–46^, we further explored potential effects of the LNP-driven mRNA expressions in the heart.

To this end, we intramuscularly injected MC3-based LNPs containing SARS-CoV-2 spike mRNA into mice (2.4 µg, ~0.1 mg kg^−1^), followed by perfusion 72 h later, DISCO clearing and staining with nanobodies against spike protein^47^. This higher dose, compared to 0.0005 mg kg^−1^, was selected, as it falls within the range recommended for reliable vaccine immunogenicity and efficacy studies in mice^48,49^, based on the body surface area normalization method (allometric scaling) for human-to-mouse dose conversion^50^. SCP-Nano analysis confirmed off-targeting of LNP-delivered mRNA to the heart after intramuscular injection, ruling out trafficking of spike protein from the injection site to the heart as a major source of the spike protein signal (Fig. 4f and Supplementary Video 3). We analyzed the cell types targeted by the LNPs at the whole body level by staining for the immune cell marker CD45 and the SARS-CoV-2 spike proteins expressed from LNP-delivered mRNA. LNP-mRNA-derived spike protein was observed in both immune cells and non-immune cells throughout the body (Supplementary Fig. 13).

To identify the specific cells that are targeted by spike mRNA-LNPs in heart tissue, we performed immunohistology on these regions (SCP-Nano-identified regions were isolated from the whole mouse bodies for further molecular exploration; for details, see the Methods section). We found that the spike protein did not co-localize with cardiomyocytes (troponin T; Supplementary Fig. 14a), immune cells (CD45; Supplementary Fig. 14a) or arteries (alpha-smooth muscle actin (αSMA); Fig. 4g and Supplementary Fig. 14b) in the heart. Instead, we found the spike protein primarily within the endothelial cells of heart capillaries (podocalyxin; Fig. 4g and Supplementary Fig. 14b).

To investigate possible molecular changes induced by mRNA expression in off-target tissues, we performed mass-spectrometry-based proteomics on SCP-Nano-identified heart regions^51^. Samples were collected from the right, middle and left heart regions from three mice per group, with eight or more samples each from PBS (control), no-cargo LNP, LNP with EGFP mRNA and LNP with spike-mRNA-injected animals. We identified 4,161 proteins, with 2,828 used for downstream analysis (Methods). Principal component analysis (PCA) demonstrated clear separation between the groups with and without mRNA expression (no-cargo LNP/PBS versus spike LNP/EGFP LNP) (Fig. 4h). Pathway analysis using the Reactome database (Methods) revealed, among others, changes related to cell metabolism and signaling and immune system in mRNA-expressing groups (versus combined no-cargo LNP + PBS groups) (Fig. 4i, Supplementary Fig. 14c and Supplementary Data 1).

We then examined proteomic changes induced by LNPs alone (no-cargo LNP versus PBS). We identified 375 differentially expressed proteins (DEPs) (240 upregulated and 135 downregulated in no-cargo LNP compared to PBS) (Fig. 4j and Supplementary Data 2). These alterations were associated with metabolic processes, including ribosome activity, translation and RNA metabolism (Reactome database analysis; Methods), with markers such as Rpl11, Rpl15, Eif4b, Rps6, Rps2 and Eif2b3 differentially regulated.

Next, we analyzed proteomic changes specifically triggered by spike mRNA expression and found 578 upregulated and 201 downregulated proteins in the LNP spike mRNA compared to no-cargo LNP samples (Fig. 4k and Supplementary Data 3). We observed notable changes in the expression of proteins related to metabolism, and RNA and protein expression, beyond what was seen in the no-cargo LNP samples (Fig. 4k). We also observed an increased immune response signal at the level of individual DEPs (Fig. 4k). Notably, we found changes in proteins related to vasculature formation and maintenance (individual differentially expressed markers: Cd34 and several members of the collagen family; vascular function score in Fig. 4l and Supplementary Table 2). EGFP mRNA expression also caused notable proteome changes in the heart (581 upregulated and 424 downregulated proteins) compared to no-cargo LNPs, primarily related to metabolic and cellular responses (Supplementary Fig. 14d and Supplementary Data 4). This suggests that not only spike mRNA but also delivery and expression of any mRNA should be carefully assessed for targeted drug delivery via LNPs. To assess whether off-targeting to the heart and the associated proteomic changes are specific to MC3-based LNPs or represent a more general phenomenon of the current generation of LNPs, we also assessed the biodistribution of LNPs using the ionizable lipid SM-102 (as used in the Moderna SARS-CoV-2 vaccine) and the Lung SORT LNPs^38,52,53^. Similar to the MC3-LNPs, we found a small but distinct number of both LNPs in the heart (Supplementary Fig. 15a). We again isolated eight or more samples for each of the LNPs (no-cargo LNPs, LNPs with EGFP mRNA and SARS-CoV-2 spike protein mRNA) and the PBS control and analyzed the proteome using mass spectrometry (Supplementary Fig. 15b–d). In the PCA plot, the LNP samples again separated from the PBS control, with the empty LNPs being closest to the control (Supplementary Fig. 15b). Similar to our observations with MC3-based LNPs, proteins associated with the vasculature were among the most dysregulated genes, including several involved in maintaining vascular structure, which were strongly downregulated (individual proteins in Supplementary Fig. 15d).

The observed LNP accumulation and proteome changes in heart tissue suggest a potential mechanism by which LNP-based mRNA vaccines could contribute to the reported cardiac complications^42–46^. Nevertheless, further investigations are warranted as the exact LNP formulations and potentially the spike mRNAs used here may differ from those in the approved mRNA vaccines. Thus, the potential causative mechanisms that we identified need to be explored further in future work.

To investigate potential common mechanisms of heart targeting among the different formulations, we next investigated the protein corona for each of the LNP types (MC3, Lung SORT, SM-102 and ALC-0315). The importance of the proteins binding to the LNP surface for targeting specific cell types was shown for the SORT series of LNP formulations^54–58^. Using mass spectrometry proteomics, we observed the presence of proteins, such as vitronectin, which can attach to endothelial cells and, thereby, facilitate the LNP targeting to the heart cells (Supplementary Fig. 15e)^59,60^. Filtering for proteins (1) that were strongly enriched in all LNP coronas; (2) whose levels on LNPs showed a statistically significant and positive correlation with the number of LNP^+^ cells in the heart (*R* > 0.5); and (3) that had known binding partners expressed in the heart (StringDB or manual curation from UniProt), we also identified ficolin-1 (Fcn1), a known binder of elastin^61^, which is strongly expressed in the heart^62^, as an additional potential mediator of LNP accumulation in the heart (Supplementary Fig. 15e).

### Visualization of DNA origami targeting different cell types

To demonstrate the broad applicability of SCP-Nano, we next investigated DNA origami nanoparticles, a modality that is currently being developed for future clinical applications. The programmable self-assembly of DNA origami^2,4,12,13^ enables the design of nanoparticles for diverse applications^3,13–15^. DNA origami offers biocompatibility, precise control over shape and size, responsiveness to environmental cues and the ability to functionalize the structure at defined locations^63–65^. This versatility has led to innovative concepts for vaccines^16^, antivirals^17,18^ and stimuli-responsive therapeutics^19,20^.

**Fig. 5:**
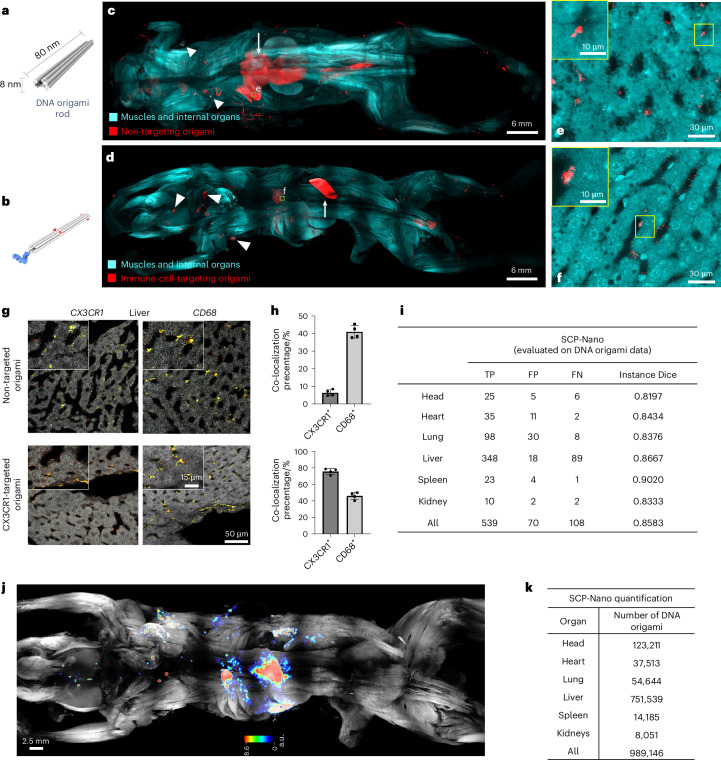
SCP-Nano reveals targeted delivery of DNA origami. **a**–**f**, Cell-level resolution biodistribution of non-targeted and CX3CR1 antibody immune-cell-targeted (illustrated on the left) origami throughout the entire mouse body (**c**,**d**) and in detailed views of the liver (**e**,**f**; illustrating single-cell resolution) 20 min after intravenous injection of 50.725 mg kg^−1^ DNA origami. **g**, Intra-tissue distribution of the immune cell and non-targeting origami (red) and co-staining with target cell marker CX3CR1 (left, yellow) and general immune cell marker CD68 (right, yellow) in confocal images of individual liver slices. **h**, Quantification of the co-localization of untargeted and CX3CR1-targeted origamis with CX3CR1^+^ and CD68^+^ cells (*n* = 3, mean ± s.d.). **i**, The prediction accuracy of DNA origami detection by SCP-Nano algorithm in different organs. **j**, Density map of CX3CR1 immune-cell-targeted origami distribution throughout the entire mouse body. **k**, SCP-Nano-based quantification of the biodistribution of CX3CR1 immune-cell-targeted origami in different organs. Source data

We generated 80-nm-long, 8-nm-diameter DNA nanorods as our base structure (Fig. 5a and Supplementary Fig. 16). This unconjugated DNA origami induced minimal immune response (Supplementary Fig. 17) and exhibited suitable circulation time for targeted delivery (Supplementary Fig. 17g). Dynamic light scattering (DLS) and fluorescence correlation spectroscopy (FCS) analyses showed that origami forms stable, monodisperse nanoparticles that are covered by a protein corona when incubated in serum (Supplementary Fig. 18). We conjugated the nanorods with CX3CR1 antibodies to target immune cells (Fig. 5b) and intravenously injected these structures at a dosage of 50.725 mg kg^−1^. Mice were euthanized, cleared and imaged 20 min after injection, as origami structures typically have a half-life in blood of several minutes^17^. As with LNPs, SCP-Nano enabled single-cell resolution imaging of various DNA origami constructs throughout entire mouse bodies (Fig. 5c–f and Supplementary Video 4).

We validated specific targeting of the antibody-conjugated DNA origami using histology after whole mouse imaging. We co-stained the liver tissues with CD68 (lysosomal/late endosomal marker labeling Kupffer cells) or CX3CR1 (mononuclear phagocyte marker) antibodies (Fig. 5g,h). Unconjugated DNA origami primarily co-localized with CD68, suggesting passive liver clearance, whereas anti-CX3CR1 DNA origami successfully targeted CX3CR1^+^ immune cells (Fig. 5g,h).

Next, we assessed the performance of our SCP-Nano deep learning pipeline on a DNA origami dataset. A test dataset of 13 3D patches (head, heart, lung, liver, spleen and kidney) was manually annotated and used to evaluate the LNP-trained algorithms. SCP-Nano achieved an average F1 score of 0.8583 (Fig. 5i), demonstrating its adaptability to detect and quantify other nanocarriers without extensive retraining. We quantified origami biodistribution by summing the intensity contrast of each segmented instance over the background. This analysis confirmed the liver as the primary target organ for unconjugated DNA origami (Fig. 5j,k).

The successful visualization and confirmation of cell-specific targeting with antibody-conjugated DNA origami underscore the value of SCP-Nano in precision nanomedicine. This capability is crucial for optimizing the efficacy and safety of future DNA origami-based treatments.

### Visualization and quantification of AAV distribution

AAVs hold promise as gene therapy nanocarriers^66^. However, optimizing their efficacy and safety demands a precise, cell-level understanding of their targeting. We applied SCP-Nano to investigate the targeting profiles of two AAV variants: (1) Retro-AAV (an AAV2 variant), engineered for retrograde transport in neurons^67^, and (2) PHP.eB-AAV (an AAV9 variant), designed to cross the blood–brain barrier for central nervous system transduction^68,69^. EGFP-encoding AAVs were injected (Retro-AAV: intramuscularly; PHP.eB-AAV: intravenously)^69–72^, and mice were perfused 2 weeks later for whole body analysis with SCP-Nano.

**Fig. 6:**
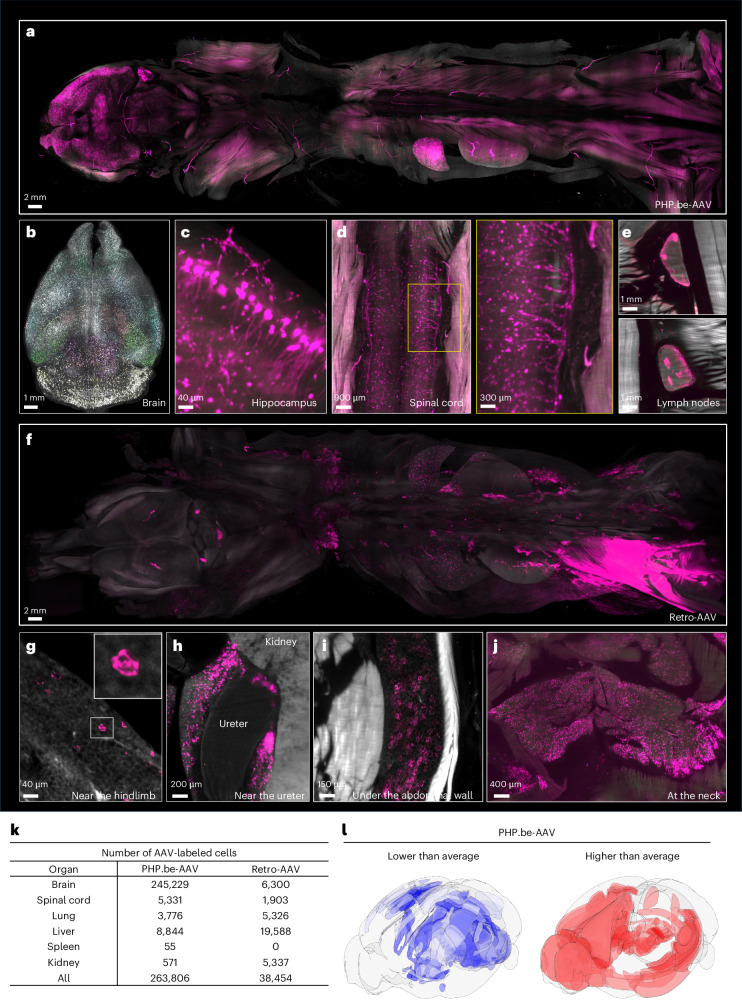
SCP-Nano quantifies targeting specificity of two types of AVVs. **a**–**e**, Biodistribution of EGFP expressed after gene delivery with PHPeB-AAV throughout the body 2 weeks after injection. Whole body view (**a**) and subregions showing PHPeB distribution in various areas, such as the brain (**b**, with density maps of cell bodies labeled by PHPeB), hippocampus (**c**), spinal cord (**d**) and the inguinal and lumbar lymph nodes (**e**). **f**–**j**, Distribution of EGFP after gene delivery with Retro-AAVs throughout the body 2 weeks after injection: whole body view (**f**) and subregions, including adipose tissue near the hindlimb (**g**), close to the ureter (**h**), beneath the abdominal wall (**i**) and at the neck (**j**). **k**, SCP-Nano quantification of the number of cells labeled by PHPeB-AAV and Retro-AAV-derived EGFP with high accuracy. **l**, Intra-brain differences in PHP.eB-AAV-based gene delivery. Blue indicates lower-than-average and red indicates higher-than-average EFP^+^ cell density. Color scales represent log_2_-transformed cell density relative to whole brain average, plotted using DELiVR^31^ on the Allen Brain Atlas reference Atlas with BrainRender. Color coding is per Allen Brain Atlas CCF3 regions.

Consistent with previous reports^69^, PHP.eB-AAV-EGFP primarily targeted the brain and spinal cord (Fig. 6a–d, Supplementary Fig. 19 and Supplementary Video 5), transducing various neuronal populations, as evidenced by the diverse sizes of targeted cells (Fig. 6c). Notably, we also observed strong signal in the inguinal and lumbar lymph nodes, a finding not previously reported (Fig. 6e).

Retro-AAV, as expected, demonstrated neuron targeting (Supplementary Fig. 20) with the labeling of axon-like extensions in various tissues^67,73^. However, we also identified a marked Retro-AAV-EGFP targeting of adipose tissue throughout the body (Fig. 6f–j and Supplementary Video 6).

To provide further insights into the mechanisms of Retro-AAV targeting of adipose tissue, we first identified the target cell type of the virus. Staining histological slices from the brown fat of Retro-AAV-injected mice for perilipin-1, a canonical lipid droplet marker, showed that most of the Retro-AAV signal was contained within adipocytes (Supplementary Fig. 21a). Furthermore, we confirmed the ability of Retro-AAV to target adipocytes in an in vitro culture of mouse adipocytes (Supplementary Fig. 21b,c).

To identify the cellular receptor in adipocytes, we inhibited previously reported receptors and co-receptors of AAV2—the parent AAV subtype of Retro-AAV—including AAVR, membrane-associated heparan sulfate proteoglycan (HSPG), αVβ5 integrin and fibroblast growth factor receptor 1 (FGFR1)^74–78^, by pre-incubation of in vitro differentiated adipocytes with polyclonal antibodies (Supplementary Fig. 21d). Inhibition of AAVR caused an 85.7% reduction in viral infection in vitro, making it a likely candidate for the primary cell entry receptor for Retro-AAV in adipocytes. Inhibition of the other proteins caused a substantially lower, but still notable, reduction in viral uptake, suggesting that they also play a role in the infection process, as previously suggested^73,79^.

To facilitate quantitative analyses, we annotated a small dataset (16 patches) and retrained our pipeline, achieving an average instance F1 score of 0.8019 (Supplementary Table 3). SCP-Nano analysis revealed approximately 40-fold higher brain cell targeting by PHP.eB-AAV compared to Retro-AAV (Fig. 6k). We further found a marked heterogeneity in the targeting of different brain regions^31^ by PHP.eB-AAV (Fig. 6l).

These results highlight the ability of SCP-Nano to reveal the cellular targets of AAVs throughout the whole mouse body. This approach is crucial for optimizing AAV design and maximizing delivery to desired cells while minimizing off-target effects.

## Discussion

SCP-Nano uses an advanced deep learning pipeline to quantify nanocarrier targeting at the single-cell level in whole mouse body imaging data. Detecting tens of millions of targeted cells requires sophisticated algorithms that segment cells based on multiple parameters, such as shape, size and brightness, even in densely populated regions. By training these algorithms on high-quality 3D data, we achieve highly accurate cell identification, as validated by F1 scores derived from ground truth comparisons. This allows SCP-Nano to perform biodistribution analysis at extremely low dose ranges (~0.0005 mg kg^−1^) and reliably detect low-intensity off-target sites in whole body imaging with an approximately 1–2-µm lateral and 6-µm axial resolution.

Alternative high-resolution biodistribution assessments rely on organ/tissue dissection followed by microscopic examination of selected sections^15,17,18^, leading to sampling biases and lack of complete whole body spatial information of drug targeting. SCP-Nano’s whole body analysis overcomes these limitations, delivering a comprehensive and unbiased view of nanocarrier distribution, including information of intra-organ delivery heterogenicity and cell type tropism (Fig. 3 and Supplementary Fig. 14). We show here that SCP-Nano can be combined with molecular methods, such as mass-spectrometry-based proteomics and immunofluorescence, and we expect that it is also compatible with other methods, such as spatial transcriptomics and proteomics imaging.

We successfully applied SCP-Nano to a wide range of clinically relevant and emerging drug delivery modalities, specifically LNPs, liposomes, polyplexes, AAVs and DNA origami, and we demonstrated that our AI-based quantification pipeline substantially outperforms previously published methods and model architectures (Fig. 2c). Retraining our deep learning quantification models for new nanocarriers required only modest new data annotation. We have made all algorithms and retraining protocols publicly available to facilitate widespread adoption.

The ability of SCP-Nano to reveal even minor off-targeting and to assess their molecular consequences by combining imaging with spatial proteomics analysis has direct implications for clinical translation. Our finding of changes in the expression of immune and vascular proteins in heart tissue after LNP spike mRNA delivery aligns with reports of myocarditis and pericarditis in a subset of individuals who received mRNA vaccines^21,44–46,80,81^. However, further research is needed to determine if similar effects occur in human subjects and to establish whether these molecular changes observed in mice are causally linked to reported clinical symptoms. We also used laboratory-produced LNP and mRNA spike formulations, which differ from approved Good Manufacturing Practice (GMP) facility-produced LNP-mRNA vaccines (Supplementary Fig. 22 and Supplementary Data 3 and 5). Regardless, these results highlight the need to investigate the biodistribution and off-target effects of LNP-based therapeutics with cell-level sensitivity across entire animal bodies. This approach enhances targeting precision and supports toxicity risk assessment by uncovering off-target activity and its implications. Notably, SCP-Nano identified off-targeting even at the lowest doses (Fig. 4d), aiding in risk stratification and informing the selection of alternative vaccine formulations or therapies where necessary.

SCP-Nano demonstrated exceptional sensitivity in visualizing cellular targets of AAVs, a critical factor in optimizing AAV-based therapeutics, particularly for gene therapy. Notably, we identified whole body Retro-AAV targeting of adipose tissue after intramuscular injection. This study reveals adipose tissue as a key target for Retro-AAV-mediated gene delivery, offering alternatives for treating obesity-related health complications.

Although SCP-Nano offers considerable advantages, it is essential to acknowledge further limitations. The clearing, imaging and deep learning analysis can be complex, but we have mitigated this by providing detailed tutorials and making our code and protocols open source (Methods and ‘Code availability’). Additionally, SCP-Nano cannot provide the dynamic and longitudinal information that live animal methods, such as PET or bioluminescence imaging, offer. Thus, as SCP-Nano is an endpoint analysis in fixed animals, it might be challenging to measure typical pharmacological parameters directly. However, combining live animal imaging methods with SCP-Nano can provide complementary information for the most comprehensive characterization of nanocarriers in pre-clinical models. Finally, nanocarriers or therapeutics that cannot be readily labeled with fluorescent dyes may require alternative labeling approaches.

In conclusion, SCP-Nano enables a quantitative, comprehensive and unbiased evaluation of nanomedicine distribution and effects at the cellular level across the entire mouse body. It holds promise for application to other model organisms and human samples, paving the way for the accelerated development of safe and precise nanocarrier-based therapeutics.

## Methods

### Materials

DLin-MC3-DMA (555308) was purchased from MedKoo Biosciences. DSPC (850365P), cholesterol (C8667) and DMG-PEG-2000 (880151P) were obtained from Sigma-Aldrich. Pur-A-Lyzer Midi Dialysis Kits (PURD35030, WMCO, 3.5 kDa) were purchased from Sigma-Aldrich. In vitro translation (IVT)-generated EGFP mRNA, spike protein mRNA and Alexa Fluor 647–labeled and Alexa Fluor 750–labeled EGFP mRNA, as well as FLuc mRNA, were purchased from RiboPro. Alexa Fluor 568–conjugated anti-GFP signal-enhancing nanobodies (gb2AF568-50) and Alexa Fluor 647–conjugated anti-GFP signal-enhancing nanobodies (gb2AF647) were purchased from ChromoTek. Propidium iodide was obtained from Sigma-Aldrich (P4864). VivoGlo luciferin was obtained from Promega. We used the following AAV constructs: Retro-AAV: (retro AAV-CAG-GFP, Addgene, 37825-AAVrg; titer ≥ 7 × 10^12^ vg ml^−1^; dilution 1:10 with 1× PBS) and PHP.eB-AAV: (PHP.eB-CAG-GFP, Addgene, 37825-PHPeB; titer ≥ 7 × 10^13^ vg ml^−1^; dilution 1:10 in 1× PBS).

### Animals

Mixed-gender C57BL/6 mice were obtained from Charles River Laboratories. The animals were housed under a 12-h light/dark cycle and had random access to food and water. The temperature was maintained at 18–23 °C, and humidity was at 40–60%. The animal experiments were conducted according to institutional guidelines of the Klinikum der Universität München/Ludwig Maximilian University of Munich and after approval of the Ethical Review Board of the Government of Upper Bavaria (Regierung von Oberbayern, Munich, Germany) and under European Directive 2010/63/EU for animal research. All experiments were performed in triplicates.

### LNP formulation

MC3-based LNPs were prepared using the ethanol dilution method. All lipids with specified molar ratios (Dlin-MC3-DMA/DSPC/cholesterol/PEG-DMG at a 50:10:38.5:1.5 molar ratio) were dissolved in ethanol absolute (<99.8%), and mRNA was dissolved in 10 mM citrate buffer (pH 4.0). Those two solutions were mixed at an aqueous-to-ethanol volume ratio of 3:1 to make a final weight ratio of 1:10 (mRNA/total lipids) and then incubated for 10 min at room temperature. The final LNP formulations were dialyzed (Pur-A-Lyzer Midi Dialysis Kits, molecular weight cutoff (MWCO) 3.5 kDa) against PBS for 2 h to remove the ethanol and neutralize the pH. For Alexa Fluor 647–labeled EGFP mRNA LNPs, 20% non-labeled UTP was replaced by Alexa Fluor 647–labeled UTP^82,83^. The hydrodynamic diameter and zeta potential of the LNPs were measured by DLS using disposable cuvettes in a Malvern Zetasizer. The encapsulation efficacy was examined using a Quanti-iT RiboGreen RNA Assay Kit (Thermo Fisher Scientific, Q33140)^84^.

For other LNP formulations:

- Lung SORT (in molar ratios): cholesterol (19.3), DSPC (5), PEG-DMG (0.8), MC3 (25), DOTAP (50) (total lipids/mRNA = 40/1, wt/wt)
- SM-102 LNP formulations (in molar ratios): cholesterol (38.5), DSPC (10), PEG-DMG (1.5), SM-102 (50) (total lipids/mRNA = 10/1, wt/wt)
- ALC-0315 LNP formulations (in molar ratios): cholesterol (38.5), DSPC (10), PEG-DMG (1.5), ALC-0315 (50) (total lipids/mRNA = 10/1, wt/wt)

### DNA origami production

The biotechnologically produced nanorod was prepared as described previously^85^. In brief, the staple strands for the nanoobject are arranged as a pseudogene, interleaved by self-excising DNAzyme cassettes. The pseudogene is cloned into a phagemid backbone, a scaffold strand for the nanoobject. The fully cloned phagemid was introduced into *Escherichia coli* JM109 cells by transformation. Using helper phage rescue, the phage particles containing the phagemid single-stranded DNA are produced in a stirred-tank bioreactor, followed by phage and DNA purification to yield pure circular ssDNA. As a prerequisite for the in vivo experiments, the ssDNA was endotoxin purified as described by Hahn et al.^86^, using Triton X-114. By adding zinc cations, the self-excising DNAzyme is activated and yields scaffold and staple strands for the nanorod. After a further DNA purification step with ethanol, the nanorod was assembled in a buffer containing 5 mM Tris, 5 mM NaCl, 1 mM EDTA and 10 mM MgCl_2_ at a DNA concentration of 50 nM (15 min at 65 °C and then 60–45 °C at 1 h per °C). To assemble fluorescence-labeled DNA nanoobjects for the optimized DISCO clearing protocol, we purchased chemically synthesized single-stranded staple strands with Atto 550 or Atto 647N modification from Integrated DNA Technologies or Biomers. The folding reactions were set up as 100-µl or 2-ml reaction solutions with UV crosslinking for 5 min^87^.

### Preparation of IgG–ssDNA conjugates

Oligonucleotides modified with 3′ thiol modification were purchased high-performance liquid chromatography (HPLC) purified and dried from Biomers. The oligos were dissolved in PBS (pH 7.2) with 5 mM TCEP and incubated for 1 h at room temperature. After three rounds of filter purification (10k Amicon Ultra 0–5-ml centrifugal filter), 10 nmol of the reduced thiol oligo was mixed with 10 equivalents of Sulfo-SMCC (sulfosuccinimidyl 4-(N-maleimidomethyl) cyclohexane 1-carboxylate; Thermo Fisher Scientific, dissolved in ddH_2_O) for 15 min. After three rounds of filter purification (10k Amicon Ultra 0–5-ml centrifugal filter), including buffer change to PBS (pH 8), 100 µg of antibody in PBS (pH 8) was added. The reaction was incubated for 4 h at 4 °C at least. The conjugate was subsequently purified by ion-exchange chromatography (Dynamic Biosensors, proFIRE) using an NaCl gradient of 150–1,000 mM in PBS (pH 7.2). The purity of the oligo–antibody conjugates was analyzed by SDS-PAGE and agarose gel.

### Assembly of antibody functionalized DNA nanorod

The assembled nanorod was purified with two rounds of PEG precipitation to remove excess staple strands, as described by Stahl et al.^88^, with slightly higher PEG 8000 (8.25% wt/vol) and NaCl (275 mM) concentrations. The nanorod was equipped with up to four handle ssDNA strands (part of the original structure as assembled above) with complementary sequences to the IgG–ssDNA conjugate. The hybridization of the IgG–ssDNA conjugate to the nanorod was done in a buffer containing 5 mM Tris, 5 mM NaCl, 1 mM EDTA and 10 mM MgCl_2_ at pH 8 overnight at 30 °C (Supplementary Fig. 16b). To concentrate the fully functionalized nanorod, we used another round of PEG purification, followed by dissolving the nanorod in Tris buffer (5 mM Tris, 5 mM NaCl, 1 mM EDTA and 5 mM MgCl_2_) at a high concentration of 4 µM. We added PEG-polylysine (methoxy-poly(ethylene glycol)-block-poly(l-lysine hydrochloride, 10 lysine repeating units, 5-kDa molecular weight (MW) PEG, Alamanda polymers)) at a nitrogen-to-phosphate (N:P) ratio of 1:1 to stabilize the nanorod against low-salt conditions and nuclease activity in vivo. For the in vivo experiments, the nanorod was diluted to 0.5 µM or 2 µM using sterile PBS to reduce the magnesium concentration to 2 mM or less. Finally, the endotoxin concentration of the fully equipped nanorod was determined using Endosafe nexgen-PTS (Charles River Laboratories) to fulfill the FDA requirement of less than 36 EU ml^−1^ for a 100-µl intravenous injection per day.

### Different nanocarriers administered to mice and dose calculations

LNPs were administered via different routes, including intranasal, intramuscular, intravenous, oral gavage and intradermal. Intranasal delivery was performed on lightly anaesthetized mice by passively presenting the sample droplet by droplet with a pipette to the mouse and waiting until each droplet entered the nose during normal breathing. Intramuscular delivery was administered into the muscles of the hindlimb. Intravenous injections were done via the tail vein. Oral gavage administration was performed with an animal feeding needle on awake mice. Intradermal injection was administered to the back of anaesthetized mice onto a small, shaved area. For all injections, 30-gauge needles were used.

The injection doses varied between 0.0005 mg kg^−1^ and 0.5 mg kg^−1^ as described above. When translating human vaccine doses to animal models, it is crucial to account for differences in body surface area rather than just body weight (allometric scaling). According to the guidelines, to estimate the equivalent dose for a mouse, one should divide the human dose by a factor of 12.3. For example, if the human dose is 30 µg (approximate dose used in mRNA vaccines for humans), the corresponding mouse dose would be approximately 2.4 µg (30 µg divided by 12.3)^50^. This conversion ensures that the administered dose elicits a similar pharmacodynamic response in mice^48,49^.

The animals were euthanized at 6 h (biodistribution) and 72 h (expression) after administration. Liposomes based on the FDA-approved drug Doxil deliver Atto 647 dye. The liposome formulation consists of hydrogenated soya phosphatidylcholine, cholesterol and PEG-modified phosphatidylethanolamine in a 55:40:5 molar ratio, with Atto 647 (AD 647, ATTO-TEC) as the cargo. These liposomes were produced using the thin film hydration method followed by size extrusion with a 200-µm filter to achieve uniformity^89^. After production, the liposomes were concentrated using an Amicon 30-kDa filter. Branched PEI delivers single-stranded Alexa Fluor 647–labeled DNA (Alexa 647-A*G*A*CGTGTGCTCTTCCGATCTTCTGCCCTCCAG ATCGGAAGAGCGTCGT*G*T*G). The particles were formulated using branched PEI with an MW of 25 kDa, combined with ssDNA labeled with 647 dye, at an N:P ratio of 15. These particles were produced through a self-assembly process, described in ref. ^90^. Liposomes and polyplexes were injected intramuscularly at a concentration of 0.1 mg kg^−1^. Different DNA origamis (untargeted origami and Cx3cr1 antibody-targeted origami) were injected intravenously at a dose of 50.725 mg kg^−1^, with various injection timepoints ranging from 20 min to 20 h. Retro-AAV and PHP.eB-AAV were injected intramuscularly and intravenously, respectively (10^13^ GC ml^−1^ in 25 μl)^67–69^. After 2 weeks, the mice were perfused and fixed.

### Protein corona preparation

Blood from wild-type Balb/c mice was collected retro-orbitally to obtain either plasma (in microcentrifuge tubes coated with EDTA, collected on ice, spun down for 15 min at 2,000 r.p.m. at 4 °C to retrieve plasma (supernatant)) or serum (in non-coated microcentrifuge tubes, blood left to clot at room temperature for 30 min, spun down for 15 min at 2,000 r.p.m. at 4 °C to retrieve serum (supernatant)). LNPs (50 µl each of the final preparations as described above, encapsulating 1 µg of unlabeled mRNA per formulation) were incubated with mouse plasm or serum for 15 min at 37 °C at a 31:1 volumetric ratio. A 0.7-M sucrose solution was prepared by dissolving solid sucrose in MilliQ water. The LNP/plasma mixture was loaded onto a 300-µl sucrose cushion centrifuged at 15,300*g* and 4 °C for 1 h. The supernatant was removed, and the pellet was washed with 1× PBS. Next, the pellet was centrifuged at 15,300*g* and 4 °C for 5 min, and the supernatant was removed. Washing was performed twice more. The protein-covered LNPs were kept dry at −20 °C upon mass spectrometry analysis.

### Perfusion and tissue preparation

Mice were deeply anesthetized using a combination of ketamine and xylazine (concentration, 2 ml per 1 kg intramuscularly). Afterwards, the chest cavity of mice was opened for intracardial perfusion with heparinized PBS (10–25 U ml^−1^ heparin dissolved in 0.01 M PBS) using 100–125-mmHg pressure for 5–10 min at room temperature until the blood was washed out. (The color of the liver changes from red to light yellow). Next, the mice were perfused with 4% paraformaldehyde (PFA) to fix the entire mouse body for 5 min. Finally, the skin was carefully removed, and the bodies were fixed in 4% PFA overnight at 4 °C and transferred to PBS for later immunostaining or directly processed for clearing as described below.

### Optimized DISCO technique to visualize dye-labeled LNP-mRNA

As we injected LNP with Alexa Fluor 647–labeled mRNA into mice, we developed a modified DISCO technique to render the mice transparent while retaining the fluorescent signal within the mouse body. A simplified transcardial circulatory system was established using a peristaltic pump (ISMATEC, REGLO Digital MS-4/8 ISM 834, reference tubing, SC0266). One reference tubing was connected by two connectors (Omnilab, 5434482) end to end and extended by additional PVC tubing (Omnilab, 5437920). The head part from a 1-ml syringe (Braun, 9166017V) was cut and inserted into the outflow PVC tubing as a connector for the perfusion needle (Leica, 39471024). Next, a PBS-perfused and PFA-fixed mouse body was placed into a 250-ml glass chamber (Omnilab, 5163279). The inflow tubing of the transcardial circulatory system was fixed below the surface of PBS in the glass chamber using adhesive tape, and the air bubbles were completely removed from the circulation tubing. After fixation, the mice were intracardially perfused with the decolorization solution (1:4 dilution of CUBIC reagent, consisting of 25 wt% N,N,N,N′-tetrakis (2-hydroxypropyl) ethylenediamine (from Sigma-Aldrich) and 15 wt% Triton X-100) for 12 h. After this, the mouse bodies were washed with PBS for 12 h, repeated three times, and then perfused with a decalcification solution (10 wt/vol% EDTA, pH 8.00, from Carl Roth) for 2 d. After another three transcardially administered PBS washes lasting 12 h each, the mice were transferred to passive clearing (no transcardial pumping) under a hood. We proceeded to incubate the mice in 70 vol% THF, 80 vol% THF and 2 × 100 vol% THF. This was followed by a 20-min treatment with DCM and then BABB until the tissues were fully transparent.

### Optimized DISCO technique for whole body immunostaining

The protein encoded by the cargo mRNA was expressed after injecting LNP-mRNA into the whole mouse body. We stained the expressed protein using signal-enhancing nanobodies to confirm the mRNA functional integrity. Decolorization and decalcification were performed as above. Then, nanobodies were dissolved in a permeabilization solution containing 0.5% Triton X-100, 1.5% goat serum (Gibco, 16210072), 0.5 mM methyl-beta-cyclodextrin (Sigma-Aldrich, 332615) and 0.2% trans-1-acetyl-4-hydroxy-L-proline (Sigma-Aldrich), and the solution was actively pumped transcardially for 6 d. After the immunostaining step, the mouse bodies passively cleared at room temperature with gentle shaking under a fume hood by incubation in 70 vol% THF, 80 vol% THF, 100 vol% THF and again 100 vol% THF for 12 h each. Then, the mouse bodies were treated with DCM for 20 min and immersed BABB until the tissue was rendered completely transparent.

The antibodies, nanobodies and dye used for mouse body immunostaining were rat anti-CD45 (BD Biosciences, 14-0451-82), SARS-CoV-2 spike antibody (GeneTex, GTX135356), Alexa Fluor 568–conjugated anti-GFP signal-enhancing nanobodies (ChromoTek, gb2AF568-50), Alexa Fluor 647–conjugated anti-GFP signal-enhancing nanobodies (ChromoTek, gb2AF647) and propidium iodide (Sigma-Aldrich, P4864).

### Pre-adipocyte differentiation and receptor blocking

#### Brown pre-adipocyte differentiation

Immortalized brown pre-adipocytes from wild-type C57Bl/6 mice^91^ were counted and seeded on chamber slides (Nunc Lab-Tek II chambered cover glass, Z734853). The fully confluent pre-adipocytes were induced with induction medium cocktail containing IBMX (0.5 mM), insulin (100 nM), indomethacin (5 µM), dexamethasome (5 µm) and rosiglitazone (1 µM) in DMEM 10% FBS for 2 d, and, afterwards, the medium was changed every 2 d to differentiation medium (DMEM, 10% FBS) containing 100 nM insulin, T3 and rosiglitazone.

#### Receptor blocking and immunostaining

On day 7 of differentiation, cells were either treated with PBS (control) or infected with AAV. In the blocking experiments, the cells were pre-treated with heparin (500 µg ml^−1^; Merck, H4784-250MG), anti-FGFR1 (20 µg ml^−1^; Invitrogen, PA5-86349), anti-integrin αVβ5 (20 µg ml^−1^; Bioss, BS-1356R) or anti-AAVR (20 µg ml^−1^; Proteintech, 21016-1-AP) antibodies for 1 h before Retro-AAV application (Addgene, 37825-AAVrg). On day 9, cells were washed with PBS and fixed in 4% PFA (pH 7.4) for 10 min at room temperature and blocked and permeabilized using 3% BSA and 0.3% Tween 20. Cells were then incubated with anti-perilipin-1 antibody (1:500; Cell Signaling Technology, 3470) overnight at 4 °C, followed by three times wash with PBS, and then incubated with GFP-Booster Alexa Fluor 568 (1:1,000; Thermo Fisher Scientific, 17363333) and goat anti-rabbit IgG Alexa Fluor 647 (1:500; Thermo Fisher Scientific, A-21244). After washing three times, slides were mounted using Dako antifade mounting medium and imaged on a Leica TCS SP8 microscope.

### Rehydration and immunostaining of cleared tissue

To confirm that the LNP entered specific cells in mouse hearts, we used rat anti-CD45 (BD Biosciences, 14-0451-82; diluted 1:500), Alexa Fluor 568 goat anti-rat IgG (H+L) antibody (Thermo Fisher Scientific, A-11077; diluted 1:500), mouse anti-troponin T (Abcam, ab8295; diluted 1:500), Alexa Fluor 568 goat anti-mouse IgG (H+L) antibody (Thermo Fisher Scientific, A-11004; diluted 1:500), mouse anti-podocalyxin (R&D Systems, MAB1556; diluted 1:500), rabbit anti-αSMA (Abcam, ab5694; diluted 1:500) and Alexa Fluor 568 goat anti-rabbit IgG antibody (Thermo Fisher Scientific, A-11036; diluted 1:500). The cleared mouse heart was rehydrated with the following steps at room temperature with gentle shaking: 100% THF, 90% THF, 70% THF, 50% THF and 0.01 M PBS. Afterwards, the heart tissue was dissected, cut into 10-µm sections and incubated in 0.01 M PBS containing 0.2% gelatin, 0.5% Triton X-100 and 5% goat serum for 1 d at 37 °C. The sections were then incubated with the primary antibodies diluted in the same solution overnight, washed twice in PBS, incubated with secondary antibodies for 4 h and washed in PBS three times.

### Confocal imaging

The heart slices, stained to differentiate cell types, were imaged using an inverted laser scanning confocal microscopy system equipped with a ×40 oil immersion lens (Zeiss, EC Plan-Neofluar ×40/1.30 oil DIC M27) and a ×25 water immersion long working distance (WD) objective lens (Leica, numerical aperture (NA) 0.95, WD = 2.5 mm), employing a z-step size of 0.3 μm.

### Light sheet imaging

We used the Miltenyi Biotec UltraMicroscope Blaze light sheet imaging system. For the full-scale mouse body, we used ×1.1 and ×4 magnification objectives (LaVision BioTec MI PLAN ×1.1/0.1 NA (WD = 17 mm) and Olympus XLFLUOR ×4 corrected/0.28 NA (WD = 10 mm), respectively). High-magnification tile scans were acquired using 25% overlap, and the light sheet width was reduced to obtain maximum illumination in the field of view. The z-step size was 6 μm, and the exposure time was 100 ms. Data collection was performed using ImSpector (Miltenyi Biotec, version 7.3.2).

### Intravital imaging

For multiphoton imaging to determine non-targeting DNA origami half-life in blood, we used an upright Zeiss LSM710 confocal microscope equipped with a Ti:Sa laser (Coherent, Chameleon Vision II) and two external photomultiplier detectors for red and green fluorescence. Eight-week-old C56BL/6N mice obtained from Charles River Laboratories were anesthetized intraperitoneally with a combination of medetomidine (0.5 mg kg^−1^), fentanyl (0.05 mg kg^−1^) and midazolam (5 mg kg^−1^) (MMF). The body temperature was monitored and maintained throughout the experiment using a rectal probe connected to a feedback-controlled heating pad. A catheter was placed in the femoral artery to administer fluorescent dye or nanoparticles. A rectangular 4 × 4-mm cranial window was drilled over the right frontoparietal cortex under continuous cooling with saline, as described^92^. The mouse was injected with FITC-dextran at 3 μl g^−1^ to identify the brain vessels and obtain a baseline image. Afterwards, the animal was positioned on the multiphoton microscope adapted for intravital imaging of small animals. Non-targeting DNA origami, conjugated with Atto 550, was injected at a concentration of 1 μM and a volume of 120 μl. Scanning was performed with time series at an 80-μm depth, using 10% laser power at 800 nm, a GAASP detector with an LP < 570-nm filter and a master gain of 600 for the FITC channel. For the nanoparticles channel, an LP > 570-nm filter with a master gain of 600 was used. In total, the imaging time did not exceed 70 min. Fluorescence analysis was conducted using FIJI software.

### Bioluminescence imaging

Different doses of LNP FLuc mRNA (0.5, 0.05, 0.005 and 0.0005 mg/kg^−1^; lipid-to-mRNA ratio, 5:1) were injected intravenously into mice. Six hours after the injection of LNP-Luc mRNA, an aqueous solution of L-luciferin (250 μl, 1.6 mg; BD Biosciences) was administered intraperitoneally. The bioluminescence signal was measured 10 min after injection and was then quantified using Living Image Software version 4.2 (Caliper Life Sciences).

### Protein corona measurement of DNA origami

DNA origami was dissolved in HEPES buffer or serum-containing media with graded concentrations (70%, 90% and 99%). The folded capillary cell containing the samples was then measured three times, with six sub-runs each, at a temperature of 25 °C, which was measured by fluorescence correlation spectroscopy.

### Viral injection procedure

C57BL/6J mice (all male, aged 6–8 weeks) from Charles River Laboratories were used for AAV injections. The virus was delivered intravenously through the tail vein and intramuscularly in the biceps femoris using a 31-gauge insulin syringe (BD Biosciences, 328438). AAV-PHP.eB-CAG-GFP (Addgene, 37825-PHPeB; titer ≥ 1 × 10^13^ vg ml^−1^; diluted 1:10 with 1× PBS) was used for intravenous injections, whereas AAV-retro-CAG-GFP (Addgene, 37825-AAVrg; titer ≥ 7 × 10^12^ vg ml^−1^; diluted 1:10 with 1× PBS) was used for intramuscular injections. Both intravenous and intramuscular injections were administered at a volume of 30 µl.

Before both injections, mice were anesthetized with isoflurane (5% for induction and 1% for maintenance in oxygen, 0.5 L min^−1^) and placed on a heating pad to maintain a body temperature of 37 °C. After injection, mice received 7.5 mg kg^−1^ carprofen subcutaneously.

### Deep learning for LNP nanocarrier detection

#### Data annotation

Annotation was performed in VR using SyGlass Annotation software (version 1.7.2). In total, nanoparticle-targeted cells were manually labeled in 31 patches of varying sizes from organs of interest throughout the body, namely from the head, heart, lungs, liver, spleen and kidneys. To determine the optimal architecture and hyperparameters, we split the VR-annotated data into two subsets: a training/validation subset and a test set. The training and validation subset comprises 21 patches containing 13,927 annotated nanoparticle instances. The test subset has 10 patches (at least one per organ of interest) with 6,424 annotated particles. This split was manually selected to be able to evaluate the trained methods on a test set that is representative of all annotated organ types.

As a pre-processing step before training, every patch was normalized to a 0–1 range by the minimum and maximum values of the patch.

#### Segmentation model development

To achieve the best segmentation performance for nanocarriers, we tested several state-of-the-art segmentation networks on our annotated dataset, including VNet^32^, U-Net++^33^, Attention U-Net^34^, UNETR^35^, SwinUNETR^36^, nnFormer^36^ and 3D U-Net^37^. The best-performing model was a 3D U-Net with six encoding layers, five decoding layers and leaky ReLU activation function, trained following the parameters and training protocol described by Isensee et at.^93^. We named the best-performing model SCP-Nano. The model was trained following a five-fold cross-validation scheme with a patch size of 128 × 128 × 128 voxels subsampled from the original patches during training time. The batch size during training was 2. The applied loss function was an average combination of Dice and cross-entropy loss. The stochastic gradient descent (SGD) optimizer was used for training at a learning rate of 0.0001. The model was trained for 1,000 epochs, and the checkpoint with the lowest loss value on the validation subset was kept as the best model for every fold. All best models trained in the five-fold cross validation scheme were ensembled for downstream evaluation and inference. We performed manual organ annotations using VR.

#### Inference and analysis

A single whole body mouse LNP scan can be up to 30,000 × 10,500 × 2,000 voxels. As the whole scan is too large for the typical RAM and VRAM of even dedicated servers, we cut up the whole body images into patches up to 500 × 500 × 500 voxels. These patches were fed into the trained segmentation model to get corresponding nanoparticle segmentations. The nanoparticle segmentation maps of the whole body scan were obtained by stitching the patch segmentations.

For quantitative analysis, we have VR to annotate six target organs in every whole body scan, including the brain, heart, lung, liver, spleen and kidneys. By having defined volumes of interest for each organ, we can overlap this information with the previously obtained nanoparticle segmentation to obtain organ-specific quantitative data. For this, we adopted the connected component analysis method from the cc3d library (https://github.com/seung-lab/connected-components-3d) to label and measure every segmented nanoparticle dot in each organ. For LNP quantitative analysis, we took into account local brightness levels, as this gives information about quantity of LNPs taken up by the cells. Specifically, the relative intensity contrast of every segmented LNP dot compared to the background intensity was calculated. The background intensity was estimated per organ by the average intensity of voxels inside the organ while excluding voxels belonging to segmented dots. Then, for every segmented LNP dot, we summed all voxels’ relative intensity contrast values inside. Finally, relative contrast values of all segmented dots were summed up to reflect the amount of LNP in a specific area or organ.

Next, we created a cellular density map based on nanoparticle segmentation to visualize nanoparticle distribution in different organs. A sliding window strategy was employed to compute segmented nanoparticles’ local relative intensity contrast values. A window size of 16 × 16 × 4 voxels was deployed in our experiments. Every voxel in this cube was then assigned with the sum of relative contrast values. Finally, using a 3D Gaussian filter, we obtained a smooth and full-resolution density map.

The SCP-Nano pipeline code is publicly available at https://github.com/erturklab/SCP-Nano.

### Sample preparation for mass spectrometry analysis

We divided the study into four groups: spike LNP, EGFP LNP, no-cargo LNP and PBS. Observing the LNP distribution in the heart, we found substantial LNP distribution in the middle wall between the left and right ventricles, whereas the other areas showed less LNP presence. Therefore, we used 20-gauge needles to manually extract three tissue samples from each heart (left, right and middle parts), totaling 45 samples.

Samples were prepared for mass spectrometry injections as described previously^51^. In brief, the tissues were washed with PBS and resuspended in SDS-lysis buffer (6% sodium dodecyl sulfate, 500 mM Tris-HCl, pH 8.5). This was followed by heating at 95 °C for 45 min at 1,000 r.p.m. in a thermomixer. The samples were then subjected to ultrasonication using a Bioruptor Pico sonication device operated at high frequency for 30 s on and 30 s off for 30 cycles. After ultrasonication, the samples were again heated at 95 °C for 45 min at 1,000 r.p.m. in a thermomixer. This was followed by protein precipitation in ice-cold acetone (80% vol/vol) overnight at −80 °C, followed by centrifugation for 15 min at 4 °C. For reduction and alkylation, the proteins were resuspended in SDC buffer and heated at 95 °C for 10 min at 1,000 r.p.m. Trypsin and LysC digestion were carried out at an enzyme/substrate ratio of 1:50, and the samples were incubated at 37 °C overnight at 1,000 r.p.m. in a thermomixer. Next, peptides were acidified using 1% trifluoroacetic acid (TFA) in 99% isopropanol in a 1:1 vol/vol ratio. The peptides were subjected to in-house-built StageTips consisting of two layers of styrene-divinylbenzene reversed-phase sulfonate (SDB-RPS; 3M Empore) membranes. Peptides were loaded on the activated (100% acetonitrile (ACN), 1% TFA in 30% methanol, 0.2% TFA, respectively) StageTips, run through the SDB-RPS membranes and washed by ethyl acetate (EtOAc) including 1% TFA, isopropanol including 1% TFA and 0.2% TFA, respectively. Peptide elution was carried out in 60 μl of 1.25% ammonia and 80% ACN (acetonitrile) and dried for 45 min at 45 °C in a SpeedVac (Eppendorf, Concentrator plus). The dried peptides were reconstituted in 8 μl of 2% ACN/0.1% TFA, and peptide concentration was estimated using Pierce Quantitative Colorimetric Peptide Assay.

#### LNP corona samples preparation

Nanoparticle samples were resuspended in 25 µl of 4% SDS-Tris-HCl buffer (comprising 4% SDS and 10 mM TCEP, pH 8.5) in PCR strips. The LNP samples were heated at 95 °C for 5 min, followed by sonication for 15 min at 4 °C. Subsequently, 25 µl of 2% SDC buffer (also containing 10 mM TCEP and 40 mM CAA) was added, and the mixture was heated again at 95 °C for 10 min. A 3-µl aliquot of a 1:1 mix of SpeedBeads was introduced, and proteins were precipitated using ethanol to a final concentration of 75% under stirring for 5 min. The beads were captured on a magnetic 96-well rack and washed twice with 100 µl of 80% ethanol. Residual ethanol was evaporated in a SpeedVac. The samples were then digested with 0.4 µg of Trypsin-LysC in 50 µl of 50 mM TEAB with 0.02% LMNG at 37 °C overnight.

#### Evotip PURE clean-up of LNP corona samples

The Evotip PURE protocol was modified to ensure highly reproducible and standardized offline C18 clean-up in a 96-well format. Initially, Evotip PURE tips were rinsed with 20 µl of Buffer B (comprising 80% ACN, water and 0.1% formic acid) and spun down at 800*g* for 60 s. The Evotips were conditioned with 10 µl of isopropanol, followed by an impulse spin at 100*g*, a 1-min incubation and an additional 4 min at 100*g* to empty the Evotips. The PURE Evotips were equilibrated in 20 µl of Buffer A (0.1% formic acid) and impulse spun at 800*g* for storage until the acidified samples were ready to load. Samples were acidified in 0.4% TFA, and the Evotip PURE was emptied by centrifuging at 800*g* for 1 min. The acidified samples were loaded onto the PURE Evotips and spun at 800*g* for 1 min. The samples were washed twice with 20 µl of Buffer A and spun down at 800*g* for 1 min. Elutions were collected in PCR strips by eluting with 20 µl of 45% Buffer B (containing 45% ACN, water and 0.1% TFA) at 450*g*. The peptides were dried in a SpeedVac and resuspended in 0.1% TFA supplemented with 0.015% DDM for mass spectrometry analyses.

### Liquid chromatography with tandem mass spectrometry

The mass spectrometry data were generated in data-independent acquisition (DIA) modes. The liquid chromatography with tandem mass spectrometry (LC−MS/MS) analysis was carried out using an EASY nanoLC 1200 (Thermo Fisher Scientific) coupled with a trapped ion mobility spectrometry quadrupole time-of-flight single-cell proteomics mass spectrometer (timsTOF SCP; Bruker Daltonik) via a CaptiveSpray nano-electrospray ion source. Peptides (100 ng) were loaded onto a 15-cm Aurora Elite UHPLC column with CaptiveSpray insert (75-μm ID, 1.7-μm C18) at 60 °C and separated using a 60-min gradient (5–20% Buffer B in 30 min, 20–29% Buffer B in 9 min, 29–45% in 6 min, 45–95% in 5 min, wash with 95% Buffer B for 5 min, 95–5% Buffer B in 5 min) at a flow rate of 300 nl min^−1^. Buffers A and B were water with 0.1 vol% formic acid and 80:20:0.1 vol% ACN:water:formic acid, respectively. Mass spectrometry data were acquired in single-shot library-free DIA mode, and the timsTOF SCP was operated in DIA/parallel accumulation serial fragmentation (PASEF) using the high-sensitivity detection-low sample amount mode. The ion accumulation and ramp time were set to 100 ms each to achieve nearly 100% duty cycle. The collision energy was ramped linearly as a function of the mobility from 59 eV at 1/K0 = 1.6 Vs cm^−2^ to 20 eV at 1/K0 = 0.6 Vs cm^−2^. The isolation windows were defined as 24 × 25 Th from *m*/*z* 400 to 1,000.

LNP corona samples were analyzed using modified chromatography. In brief, 50 ng of peptides per injection was loaded on a 5.5-cm High Throughput μPAC Neo HPLC Column (Thermo Fisher Scientific) and analyzed using an 80-min active gradient method at a flow rate of 250 nl min^−1^.

### Proteomics data processing

diaPASEF raw files were searched against the mouse UniProt database using DIA-NN^94^. A peptide length range of seven amino acids was considered for the search, including N-terminal acetylation. Methionine oxidation was set as variable modifications and cysteine carbamidomethylation as fixed modification. Enzyme specificity was set to Trypsin/P with two missed cleavages. The FASTA digest for the library-free search was enabled to predict library generation. The false discovery rate was set to 1% at precursor and global protein levels. The match-between-runs (MBR) feature was enabled, and the quantification mode was set to ‘Robust LC (high precision)’. The Protein Group column in DIA-NN’s report was used to identify the protein group, and PG.MaxLFQ was used to calculate the differential expression.

### Proteomics downstream data analysis

#### Proteomics analysis of MC3 in the heart tissue

1. Proteomics data analysis
  Scanpy (version 1.10.2) and anndata (version 0.10.8) packages in Python 3.10 were used to implement the downstream analysis pipeline to analyze the LC–MS samples. Nine independent samples were analyzed from each group of three animals, except for PBS, where we had eight independent samples.
2. Quality control
  All proteins expressed in less than half of the samples in each group were filtered out.
3. Data processing
  The data were log transformed and normalized per sample. The missing values were input using KNNImputer (n_neighbors = 5) from the sklearn package (version 1.5.1). To correct the batch effect across the different runs, the data further underwent batch correction using ComBat from scib tools (https://github.com/theislab/scib) (version 1.1.3).
4. Dendrograms
  With Scanpy’s dendrogram function, hierarchical linkage clustering was calculated on a Pearson correlation matrix over groups for 50 averaged principal components.
5. Differential expression tests

To identify differentially regulated proteins across two groups (for example, spike LNP versus no-cargo LNP), Scanpy’s method that ranks gene groups using *t*-tests was used. The maximal *P* value and minimal log fold change were used to identify DEPs. The thresholds are *P* < 0.05 and |log fold change| > 0.5. These DEPs were further used to plot volcano plots. Using the Reactome database and GProfiler, we performed enrichment analysis on genes with DEPs. Visualization was focused on the broadest categories of biological pathways.

The vascular-related genes were manually curated using related pathways from a publicly available database (Reactome). The vascular-related gene sets were tested individually using a normalized mean expression score for the different groups.

#### Proteomics analysis of SM-102 and Lung SORT formulations in the heart tissue

1. Proteomics data analysis
  Mouse samples were analyzed using Scanpy (version 1.10.2) and anndata (version 0.10.8) in Python 3.10. The study included two different formulations: SM-102 and Lung SORT. Samples were categorized into three groups: no-cargo LNP, EGFP and spike protein. Each group comprised samples from three animals, resulting in nine samples per group for each formulation. Additionally, nine control samples consisting of PBS were analyzed from three animals.
2. Quality control
  All proteins expressed in less than half of the samples in each group were filtered out, resulting in 2,191 proteins used for downstream analyses.
3. Data processing
  The data were log transformed and normalized per sample. The missing values were input using KNNImputer (n_neighbors = 5) from the sklearn package (version 1.5.1).
4. Differential expression tests

To identify differentially regulated proteins across two groups (for example, SM-102 spike versus PBS), Scanpy’s method that ranks gene groups using *t*-tests was used. The maximal *P* value and minimal log fold change were used to identify DEPs. The thresholds are *P* < 0.05 and |log fold change| > 0.5. These DEPs were further used to plot volcano plots.

The normalized intensity of selected DEPs was plotted for each formulation and group. Additionally, a Student’s *t*-test was conducted to assess the statistical significance of differences compared to the control group (PBS).

#### Protein corona of LNP formulations

1. Proteomics data analysis
  Mouse samples were analyzed using Scanpy (version 1.10.2) and anndata (version 0.10.8) in Python 3.10. The study included plasma from four different LNP formulations: MC3, SM-102, BioNTech and Lung SORT. Each group comprised samples from three animals. Additionally, control samples consisting of PBS were analyzed from three animals.
2. Quality control
  All proteins expressed in less than half of the samples in each group were filtered out.
3. Data processing
  The data were log transformed and normalized per sample. The missing values were input using KNNImputer (n_neighbors = 5) from the sklearn package (version 1.5.1).
4. Differential expression tests

To identify differentially regulated proteins across two groups (for example, MC3 versus PBS), Scanpy’s method that ranks gene groups using *t*-tests was used. The maximal *P* value and minimal log fold change were used to identify DEPs. The thresholds are *P* < 0.05 and |log fold change| > 0.5. These DEPs were further used to plot volcano plots. The DEPs of each of the formulations with comparison to the control PBS were determined. To investigate the potential protein corona, we identified common differentially binding proteins through pairwise comparisons between each formulation and the PBS control. Subsequently, the normalized intensities of common differentially binding proteins were plotted, and a Student’s *t*-test was performed to determine the statistical significance of differences relative to the control group (PBS). Potential binders for these proteins were determined using the StringDB Python package and manual curation from UniProt.

### Statistics and reproducibility

All experiments were independently repeated at least three times with similar results. Relevant statistical tests are described in the figure legends or in the relevant sections above. Representative data, including micrographs, are presented where applicable.

### Reporting summary

Further information on research design is available in the Nature Portfolio Reporting Summary linked to this article.

## Online content

Any methods, additional references, Nature Portfolio reporting summaries, source data, extended data, supplementary information, acknowledgements, peer review information; details of author contributions and competing interests; and statements of data and code availability are available at 10.1038/s41587-024-02528-1.

## Supplementary information

Supplementary Information Supplementary Figs. 1–22 and Supplementary Tables 1–3.

Reporting Summary

Supplementary Video 1 Distribution of lipid nanoparticles (in magenta) in the lung after a 0.0005-mg kg^−1^ intranasal injection after 6 h.

Supplementary Video 2 Distribution of EGFP protein expression (in green) in the heart 72 h after intramuscular injection of 0.0005 mg kg^−1^ LNP EGFP mRNA.

Supplementary Video 3 Distribution of LNP spike mRNA (in magenta) in the heart 6 h after intramuscular injection of a dose of 0.0005 mg kg^−1^.

Supplementary Video 4 Distribution of DNA origami (in red) at the cell level throughout the entire mouse body, especially in the spleen, liver and lung.

Supplementary Video 5 Distribution of GFP expressed from PHP.eB-AAV-delivered DNA 2 weeks after intravenous injection in different brain regions. Scale bar, 1 mm. Color coding is per Allen Brain Atlas CCF3 regions.

Supplementary Video 6 3D visualization of the distribution of GFP expressed from RetroAAV-delivered DNA throughout the entire mouse body at cell-level resolution, with GFP in cyan and the background in gray.

Supplementary Data 1 Statistical source data.

Supplementary Data 2 Statistical source data.

Supplementary Data 3 Statistical source data.

Supplementary Data 4 Statistical source data.

Supplementary Data 5 Statistical source data.

## Source data

Source Data Fig. 3 Statistical source data.

Source Data Fig. 5 Statistical source data.

## Data Availability

All data that support the findings of this study are available from the corresponding author upon reasonable request. The proteomics data were uploaded to the PRIDE partner repository with the dataset identifier PXD056871 (ref. ^95^), accessible at http://proteomecentral.proteomexchange.org. Whole body imaging data are available upon reasonable request. Source data are provided with this paper.

## Code availability

The SCP-Nano pipeline code is available on GitHub at https://github.com/erturklab/SCP-Nano (ref. ^96^).

## References

[1] Yin, H. et al. Therapeutic genome editing by combined viral and non-viral delivery of CRISPR system components in vivo. *Nat. Biotechnol.* **34**, 328–333 (2016). 26829318 10.1038/nbt.3471 PMC5423356

[2] Paunovska, K., Loughrey, D. & Dahlman, J. E. Drug delivery systems for RNA therapeutics. *Nat. Rev. Genet.* **23**, 265–280 (2022). 34983972 10.1038/s41576-021-00439-4 PMC8724758

[3] Mulligan, M. J. et al. Phase I/II study of COVID-19 RNA vaccine BNT162b1 in adults. *Nature* **586**, 589–593 (2020). 32785213 10.1038/s41586-020-2639-4

[4] Wang, C. et al. Ultrasound-responsive low-dose doxorubicin liposomes trigger mitochondrial DNA release and activate cGAS-STING-mediated antitumour immunity. *Nat. Commun.* **14**, 3877 (2023). 37391428 10.1038/s41467-023-39607-x PMC10313815

[5] Casper, J. et al. Polyethylenimine (PEI) in gene therapy: current status and clinical applications. *J. Control. Release* **362**, 667–691 (2023). 37666302 10.1016/j.jconrel.2023.09.001

[6] Zhao, Z., Anselmo, A. C. & Mitragotri, S. Viral vector-based gene therapies in the clinic. *Bioeng. Transl. Med.* **7**, e10258 (2022). 35079633 10.1002/btm2.10258 PMC8780015

[7] Zeng, Y. C. et al. Fine tuning of CpG spatial distribution with DNA origami for improved cancer vaccination. *Nat. Nanotechnol.* **19**, 1055–1065 (2024). 38491184 10.1038/s41565-024-01615-3

[8] Wagenbauer, K. F. et al. Programmable multispecific DNA-origami-based T-cell engagers. *Nat. Nanotechnol.* **18**, 1319–1326 (2023). 37591933 10.1038/s41565-023-01471-7 PMC10656288

[9] Wamhoff, E.-C. et al. Enhancing antibody responses by multivalent antigen display on thymus-independent DNA origami scaffolds. *Nat. Commun.* **15**, 795 (2024). 38291019 10.1038/s41467-024-44869-0 PMC10828404

[10] Wang, Y. et al. A DNA robotic switch with regulated autonomous display of cytotoxic ligand nanopatterns. *Nat. Nanotechnol.* **19**, 1366–1374 (2024). 38951595 10.1038/s41565-024-01676-4 PMC11405282

[11] Sigl, C. et al. Programmable icosahedral shell system for virus trapping. *Nat. Mater.* **20**, 1281–1289 (2021). 34127822 10.1038/s41563-021-01020-4 PMC7611604

[12] Dey, S. et al. DNA origami. *Nat. Rev. Methods Primers* **1**, 13 (2021).

[13] Hadjidemetriou, M. & Kostarelos, K. Evolution of the nanoparticle corona. *Nat. Nanotechnol.* **12**, 288–290 (2017). 28383044 10.1038/nnano.2017.61

[14] Schwenck, J. et al. Advances in PET imaging of cancer. *Nat. Rev. Cancer* **23**, 474–490 (2023). 37258875 10.1038/s41568-023-00576-4

[15] Zhang, Q. et al. DNA origami as an in vivo drug delivery vehicle for cancer therapy. *ACS Nano* **8**, 6633–6643 (2014). 24963790 10.1021/nn502058j

[16] Biancacci, I. et al. Optical imaging of the whole-body to cellular biodistribution of clinical-stage PEG-b-pHPMA-based core-crosslinked polymeric micelles. *J. Control. Release* **328**, 805–816 (2020). 33010332 10.1016/j.jconrel.2020.09.046 PMC7611891

[17] Ponnuswamy, N. et al. Oligolysine-based coating protects DNA nanostructures from low-salt denaturation and nuclease degradation. *Nat. Commun.* **8**, 15654 (2017). 28561045 10.1038/ncomms15654 PMC5460023

[18] Perrault, S. D. & Shih, W. M. Virus-inspired membrane encapsulation of DNA nanostructures to achieve in vivo stability. *ACS Nano* **8**, 5132–5140 (2014). 24694301 10.1021/nn5011914 PMC4046785

[19] Patone, M. et al. Risks of myocarditis, pericarditis, and cardiac arrhythmias associated with COVID-19 vaccination or SARS-CoV-2 infection. *Nat. Med.* **28**, 410–422 (2022). 34907393 10.1038/s41591-021-01630-0 PMC8863574

[20] Rosenblum, H. G. et al. Safety of mRNA vaccines administered during the initial 6 months of the US COVID-19 vaccination programme: an observational study of reports to the Vaccine Adverse Event Reporting System and v-safe. *Lancet Infect. Dis.* **22**, 802–812 (2022). 35271805 10.1016/S1473-3099(22)00054-8 PMC8901181

[21] Bozkurt, B., Kamat, I. & Hotez, P. J. Myocarditis with COVID-19 mRNA vaccines. *Circulation* **144**, 471–484 (2021). 10.1161/CIRCULATIONAHA.121.056135 PMC8340726 34281357

[22] Montgomery, J. et al. Myocarditis following immunization with mRNA COVID-19 vaccines in members of the US military. *JAMA Cardiol.* **6**, 1202–1206 (2021). 34185045 10.1001/jamacardio.2021.2833 PMC8243257

[23] Akinc, A. et al. The Onpattro story and the clinical translation of nanomedicines containing nucleic acid-based drugs. *Nat. Nanotechnol.* **14**, 1084–1087 (2019). 31802031 10.1038/s41565-019-0591-y

[24] Ertürk, A. et al. Three-dimensional imaging of solvent-cleared organs using 3DISCO. *Nat. Protoc.* **7**, 1983–1995 (2012). 23060243 10.1038/nprot.2012.119

[25] Cai, R. et al. Panoptic imaging of transparent mice reveals whole-body neuronal projections and skull–meninges connections. *Nat. Neurosci.* **22**, 317–327 (2019). 30598527 10.1038/s41593-018-0301-3 PMC6494982

[26] Retelletti Brogi, S., Derrien, M. & Hur, J. In-depth assessment of the effect of sodium azide on the optical properties of dissolved organic matter. *J. Fluoresc.* **29**, 877–885 (2019). 31218596 10.1007/s10895-019-02398-w

[27] Susaki, E. A. et al. Versatile whole-organ/body staining and imaging based on electrolyte-gel properties of biological tissues. *Nat. Commun.* **11**, 1982 (2020). 32341345 10.1038/s41467-020-15906-5 PMC7184626

[28] Mitchell, M. J. et al. Engineering precision nanoparticles for drug delivery. *Nat. Rev. Drug Discov.* **20**, 101–124 (2021). 33277608 10.1038/s41573-020-0090-8 PMC7717100

[29] Al-Maskari, R., Paetzold, J. C., Horvath, I. & Erturk, A. On the pitfalls of deep image segmentation for lightsheet microscopy. In *Medical Imaging with Deep Learning. MIDL 2022 Short Papers* https://openreview.net/pdf?id=3Krfu84W-Wx (2022).

[30] Pan, C. et al. Deep learning reveals cancer metastasis and therapeutic antibody targeting in the entire body. *Cell* **179**, 1661–1676 (2019). 31835038 10.1016/j.cell.2019.11.013 PMC7591821

[31] Kaltenecker, D. et al. Virtual reality empowered deep learning analysis of brain activity. *Nat. Methods* **21**, 1306–1315 (2024). 10.1038/s41592-024-02245-2 PMC11239522 38649742

[32] Milletari, F., Navab, N. & Ahmadi, S.-A. V-Net: fully convolutional neural networks for volumetric medical image segmentation. In *Proc. of the 2016 Fourth International Conference on 3D Vision (3DV)*10.1109/3DV.2016.79 (IEEE, 2016).

[33] Zhou, Z., Rahman Siddiquee, M. M., Tajbakhsh, N. & Liang, J. UNet++: a nested U-Net architecture for medical image segmentation. In *Deep Learning in Medical Image Analysis and Multimodal Learning for Clinical Decision Support* (eds Stoyanov, D. et al.) 10.1007/978-3-030-00889-5_1 (Springer, 2018). 10.1007/978-3-030-00889-5_1 PMC7329239 32613207

[34] Oktay, O. et al. Attention U-Net: learning where to look for the pancreas. Preprint at https://arxiv.org/abs/1804.03999 (2018).

[35] Hatamizadeh, A. et al. UNETR: transformers for 3D medical image segmentation In *Proc. of the IEEE/CVF Winter Conference on Applications of Computer Vision (WACV)*10.1109/WACV51458.2022.00181 (IEEE, 2022).

[36] Hatamizadeh, A. et al. Swin UNETR: Swin transformers for semantic segmentation of brain tumors in MRI images. In *Brainlesion: Glioma, Multiple Sclerosis, Stroke and Traumatic Brain Injuries* (eds Crimi, A. & Bakas, S.) 10.1007/978-3-031-08999-2_22 (Springer, 2022).

[37] Çiçek, Ö., Abdulkadir, A., Lienkamp, S. S., Brox, T. & Ronneberger, O. 3D U-Net: learning dense volumetric segmentation from sparse annotation. In *Medical Image Computing and Computer-Assisted Intervention–MICCAI 2016* (eds Ourselin, S., Joskowicz, L., Sabuncu, M., Unal, G. & Wells, W.) 10.1007/978-3-319-46723-8_49 (Springer, 2016).

[38] Hou, X., Zaks, T., Langer, R. & Dong, Y. Lipid nanoparticles for mRNA delivery. *Nat. Rev. Mater.* **6**, 1078–1094 (2021). 34394960 10.1038/s41578-021-00358-0 PMC8353930

[39] Bernard, M.-C. et al. The impact of nucleoside base modification in mRNA vaccine is influenced by the chemistry of its lipid nanoparticle delivery system. *Mol. Ther. Nucleic Acids* **32**, 794–806 (2023). 37346973 10.1016/j.omtn.2023.05.004 PMC10280092

[40] Suzuki, Y. et al. Design and lyophilization of lipid nanoparticles for mRNA vaccine and its robust immune response in mice and nonhuman primates. *Mol. Ther. Nucleic Acids* **30**, 226–240 (2022). 36187052 10.1016/j.omtn.2022.09.017 PMC9508692

[41] Shepherd, S. J. et al. Throughput-scalable manufacturing of SARS-CoV-2 mRNA lipid nanoparticle vaccines. *Proc. Natl Acad. Sci. USA* **120**, e2303567120 (2023). 10.1073/pnas.2303567120 PMC10438381 37556502

[42] Tsilingiris, D., Vallianou, N. G., Karampela, I., Liu, J. & Dalamaga, M. Potential implications of lipid nanoparticles in the pathogenesis of myocarditis associated with the use of mRNA vaccines against SARS-CoV-2. *Metab. Open* **13**, 100159 (2022). 10.1016/j.metop.2021.100159 PMC8677426 34938983

[43] Stowe, J., Miller, E., Andrews, N. & Whitaker, H. J. Risk of myocarditis and pericarditis after a COVID-19 mRNA vaccine booster and after COVID-19 in those with and without prior SARS-CoV-2 infection: a self-controlled case series analysis in England. *PLoS Med.* **20**, e1004245 (2023). 37285378 10.1371/journal.pmed.1004245 PMC10286992

[44] Das, B. B., Moskowitz, W. B., Taylor, M. B. & Palmer, A. Myocarditis and pericarditis following mRNA COVID-19 vaccination: what do we know so far? *Children (Basel)* **8**, 607 (2021). 10.3390/children8070607 PMC8305058 34356586

[45] Barda, N. et al. Safety of the BNT162b2 mRNA Covid-19 vaccine in a nationwide setting. *N. Engl. J. Med.* **385**, 1078–1090 (2021). 10.1056/NEJMoa2110475 PMC8427535 34432976

[46] Alami, A. et al. Risk of myocarditis and pericarditis in mRNA COVID-19-vaccinated and unvaccinated populations: a systematic review and meta-analysis. *BMJ Open* **13**, e065687 (2023). 10.1136/bmjopen-2022-065687 PMC10314577 37339840

[47] Kong, J. et al. Pulmonary fat embolism: a potentially new fatal complication of SARS-CoV-2 infection. A case report. *BMC Infect. Dis.* **23**, 576 (2023). 37667198 10.1186/s12879-023-08559-4 PMC10478277

[48] Ji, R.-R. et al. BNT162b2 vaccine encoding the SARS-CoV-2 P2 S protects transgenic hACE2 mice against COVID-19. *Vaccines (Basel)* **9**, 324 (2021). 10.3390/vaccines9040324 PMC8066210 33915773

[49] Vogel, A. B. et al. BNT162b vaccines protect rhesus macaques from SARS-CoV-2. *Nature* **592**, 283–289 (2021). 33524990 10.1038/s41586-021-03275-y

[50] Nair, A. B. & Jacob, S. A simple practice guide for dose conversion between animals and human. *J. Basic Clin. Pharm.* **7**, 27–31 (2016). 27057123 10.4103/0976-0105.177703 PMC4804402

[51] Bhatia, H. S. et al. Spatial proteomics in three-dimensional intact specimens. *Cell* **185**, 5040–5058 (2022). 36563667 10.1016/j.cell.2022.11.021

[52] Hassett, K. J. et al. Optimization of lipid nanoparticles for intramuscular administration of mRNA vaccines. *Mol. Ther. Nucleic Acids* **15**, 1–11 (2019). 30785039 10.1016/j.omtn.2019.01.013 PMC6383180

[53] Cheng, Q. et al. Selective organ targeting (SORT) nanoparticles for tissue-specific mRNA delivery and CRISPR–Cas gene editing. *Nat. Nanotechnol.* **15**, 313–320 (2020). 32251383 10.1038/s41565-020-0669-6 PMC7735425

[54] Walkey, C. D. et al. Protein corona fingerprinting predicts the cellular interaction of gold and silver nanoparticles. *ACS Nano* **8**, 2439–2455 (2014). 24517450 10.1021/nn406018q

[55] Zhang, Y., Wu, J. L. Y., Lazarovits, J. & Chan, W. C. W. An analysis of the binding function and structural organization of the protein corona. *J. Am. Chem. Soc.* **142**, 8827–8836 (2020). 32293877 10.1021/jacs.0c01853

[56] Dilliard, S. A., Cheng, Q. & Siegwart, D. J. On the mechanism of tissue-specific mRNA delivery by selective organ targeting nanoparticles. *Proc. Natl Acad. Sci. USA* **118**, e2109256118 (2021). 10.1073/pnas.2109256118 PMC8719871 34933999

[57] Ngo, W. et al. Identifying cell receptors for the nanoparticle protein corona using genome screens. *Nat. Chem. Biol.* **18**, 1023–1031 (2022). 35953550 10.1038/s41589-022-01093-5

[58] Dilliard, S. A. et al. The interplay of quaternary ammonium lipid structure and protein corona on lung-specific mRNA delivery by selective organ targeting (SORT) nanoparticles. *J. Control. Release* **361**, 361–372 (2023). 37536547 10.1016/j.jconrel.2023.07.058 PMC10826900

[59] Li, R. et al. Vitronectin increases vascular permeability by promoting VE-cadherin internalization at cell junctions. *PLoS ONE* **7**, e37195 (2012). 22606350 10.1371/journal.pone.0037195 PMC3350505

[60] Ayloo, S. et al. Pericyte-to-endothelial cell signaling via vitronectin-integrin regulates blood–CNS barrier. *Neuron* **110**, 1641–1655 (2022). 35294899 10.1016/j.neuron.2022.02.017 PMC9119930

[61] Harumiya, S. et al. Characterization of ficolins as novel elastin-binding proteins and molecular cloning of human ficolin-1. *J. Biochem.* **120**, 745–751 (1996). 8947836 10.1093/oxfordjournals.jbchem.a021474

[62] Mo, X. G. et al. NCF2, MYO1F, S1PR4, and FCN1 as potential noninvasive diagnostic biomarkers in patients with obstructive coronary artery: a weighted gene co-expression network analysis. *J. Cell. Biochem.* **120**, 18219–18235 (2019). 31245869 10.1002/jcb.29128 PMC6771964

[63] Wiraja, C. et al. Framework nucleic acids as programmable carrier for transdermal drug delivery. *Nat. Commun.* **10**, 1147 (2019). 30850596 10.1038/s41467-019-09029-9 PMC6408537

[64] Wang, P. et al. Visualization of the cellular uptake and trafficking of DNA origami nanostructures in cancer cells. *J. Am. Chem. Soc.* **140**, 2478–2484 (2018). 29406750 10.1021/jacs.7b09024 PMC7261494

[65] Bastings, M. M. C. et al. Modulation of the cellular uptake of DNA origami through control over mass and shape. *Nano Lett.* **18**, 3557–3564 (2018). 29756442 10.1021/acs.nanolett.8b00660

[66] Kulkarni, J. A. et al. The current landscape of nucleic acid therapeutics. *Nat. Nanotechnol.* **16**, 630–643 (2021). 34059811 10.1038/s41565-021-00898-0

[67] Tervo, D. G. R. et al. A designer AAV variant permits efficient retrograde access to projection neurons. *Neuron* **92**, 372–382 (2016). 27720486 10.1016/j.neuron.2016.09.021 PMC5872824

[68] Deverman, B. E. et al. Cre-dependent selection yields AAV variants for widespread gene transfer to the adult brain. *Nat. Biotechnol.* **34**, 204–209 (2016). 26829320 10.1038/nbt.3440 PMC5088052

[69] Chan, K. Y. et al. Engineered AAVs for efficient noninvasive gene delivery to the central and peripheral nervous systems. *Nat. Neurosci.* **20**, 1172–1179 (2017). 28671695 10.1038/nn.4593 PMC5529245

[70] Chen, Z., Fan, G., Li, A., Yuan, J. & Xu, T. rAAV2-retro enables extensive and high-efficient transduction of lower motor neurons following intramuscular injection. *Mol. Ther. Methods Clin. Dev.* **17**, 21–33 (2020). 31890738 10.1016/j.omtm.2019.11.006 PMC6926343

[71] Tosolini, A. P. & Sleigh, J. N. Intramuscular delivery of gene therapy for targeting the nervous system. *Front. Mol. Neurosci.* **13**, 129 (2020). 32765219 10.3389/fnmol.2020.00129 PMC7379875

[72] Mathiesen, S. N., Lock, J. L., Schoderboeck, L., Abraham, W. C. & Hughes, S. M. CNS transduction benefits of AAV-PHP.eB over AAV9 are dependent on administration route and mouse strain. *Mol. Ther. Methods Clin. Dev.* **19**, 447–458 (2020). 33294493 10.1016/j.omtm.2020.10.011 PMC7683292

[73] Bates, R., Huang, W. & Cao, L. Adipose tissue: an emerging target for Adeno-associated viral vectors. *Mol. Ther. Methods Clin. Dev.* **19**, 236–249 (2020). 33102616 10.1016/j.omtm.2020.09.009 PMC7566077

[74] Pillay, S. et al. An essential receptor for adeno-associated virus infection. *Nature* **530**, 108–112 (2016). 26814968 10.1038/nature16465 PMC4962915

[75] Zhang, R. et al. Divergent engagements between adeno-associated viruses with their cellular receptor AAVR. *Nat. Commun.* **10**, 3760 (2019). 31434885 10.1038/s41467-019-11668-x PMC6704107

[76] Summerford, C. & Samulski, R. J. Membrane-associated heparan sulfate proteoglycan is a receptor for adeno-associated virus type 2 virions. *J. Virol.* **72**, 1438–1445 (1998). 9445046 10.1128/jvi.72.2.1438-1445.1998 PMC124624

[77] Summerford, C., Bartlett, J. S. & Samulski, R. J. αVβ5 integrin: a co-receptor for adeno-associated virus type 2 infection. *Nat. Med.* **5**, 78–82 (1999). 9883843 10.1038/4768

[78] Qing, K. et al. Human fibroblast growth factor receptor 1 is a co-receptor for infection by adeno-associated virus 2. *Nat. Med.* **5**, 71–77 (1999). 9883842 10.1038/4758

[79] Meyer, N. L. & Chapman, M. S. Adeno-associated virus (AAV) cell entry: structural insights. *Trends Microbiol.* **30**, 432–451 (2022). 34711462 10.1016/j.tim.2021.09.005 PMC11225776

[80] Heidecker, B. et al. Myocarditis following COVID-19 vaccine: incidence, presentation, diagnosis, pathophysiology, therapy, and outcomes put into perspective. A clinical consensus document supported by the Heart Failure Association of the European Society of Cardiology (ESC) and the ESC Working Group on Myocardial and Pericardial Diseases. *Eur. Heart Fail.* **24**, 2000–2018 (2022). 10.1002/ejhf.2669 PMC9538893 36065751

[81] Qaisar, I. & Sunmboye, K. A case of severe ANCA associated vasculitis after COVID-19 vaccination. *Ann. Rheum. Dis.*10.1136/annrheumdis-2022-eular.2748 (2022).

[82] Kranz, L. M. et al. Systemic RNA delivery to dendritic cells exploits antiviral defence for cancer immunotherapy. *Nature* **534**, 396–401 (2016). 27281205 10.1038/nature18300

[83] Kreiter, S. et al. Mutant MHC class II epitopes drive therapeutic immune responses to cancer. *Nature* **520**, 692–696 (2015). 25901682 10.1038/nature14426 PMC4838069

[84] Schober, G. B., Story, S. & Arya, D. P. A careful look at lipid nanoparticle characterization: analysis of benchmark formulations for encapsulation of RNA cargo size gradient. *Sci. Rep.* **14**, 2403 (2024). 38287070 10.1038/s41598-024-52685-1 PMC10824725

[85] Praetorius, F. et al. Biotechnological mass production of DNA origami. *Nature* **552**, 84–87 (2017). 29219963 10.1038/nature24650

[86] Hahn, J., Wickham, S. F. J., Shih, W. M. & Perrault, S. D. Addressing the instability of DNA nanostructures in tissue culture. *ACS Nano* **8**, 8765–8775 (2014). 25136758 10.1021/nn503513p PMC4174095

[87] Gerling, T., Kube, M., Kick, B. & Dietz, H. Sequence-programmable covalent bonding of designed DNA assemblies. *Sci. Adv.* **4**, eaau1157 (2018). 10.1126/sciadv.aau1157 PMC6097813 30128357

[88] Stahl, E., Martin, T. G., Praetorius, F. & Dietz, H. Facile and scalable preparation of pure and dense DNA origami solutions. *Angw. Chem. Int. Ed. Engl.* **53**, 12735–12740 (2014). 10.1002/anie.201405991 PMC4253120 25346175

[89] Syed, A. M. et al. Liposome imaging in optically cleared tissues. *Nano Lett.* **20**, 1362–1369 (2020). 31928014 10.1021/acs.nanolett.9b04853

[90] Dai, Z., Gjetting, T., Mattebjerg, M. A., Wu, C. & Andresen, T. L. Elucidating the interplay between DNA-condensing and free polycations in gene transfection through a mechanistic study of linear and branched PEI. *Biomaterials* **32**, 8626–8634 (2011). 21862120 10.1016/j.biomaterials.2011.07.044

[91] Pramme-Steinwachs, I., Jastroch, M. & Ussar, S. Extracellular calcium modulates brown adipocyte differentiation and identity. *Sci. Rep.* **7**, 8888 (2017). 28827782 10.1038/s41598-017-09025-3 PMC5567186

[92] Khalin, I. et al. Ultrabright fluorescent polymeric nanoparticles with a stealth pluronic shell for live tracking in the mouse brain. *ACS Nano* **14**, 9755–9770 (2020). 32680421 10.1021/acsnano.0c01505

[93] Isensee, F. et al. nnU-Net: self-adapting framework for U-Net-based medical image segmentation. Preprint at https://arxiv.org/abs/1809.10486 (2018).

[94] Demichev, V., Messner, C. B., Vernardis, S. I., Lilley, K. S. & Ralser, M. DIA-NN: neural networks and interference correction enable deep proteome coverage in high throughput. *Nat. Methods* **17**, 41–44 (2020). 31768060 10.1038/s41592-019-0638-x PMC6949130

[95] Luo, J. et al. Nanocarrier imaging at single-cell resolution across entire mouse bodies with deep learning. *PRIDE* https://www.ebi.ac.uk/pride/archive/projects/PXD056871 (2024). 10.1038/s41587-024-02528-1 PMC12700832 39809933

[96] Luo, J. et al. Nanocarrier imaging at single-cell resolution across entire mouse bodies with deep learning. *GitHub* https://github.com/erturklab/SCP-Nano (2024). 10.1038/s41587-024-02528-1 PMC12700832 39809933

