# Supplementary material for: Nanocarrier imaging at single-cell resolution across entire mouse bodies with deep learning
Source: Nat Biotechnol. 2025 Jan 14;43(12):2009–22. doi: 10.1038/s41587-024-02528-1 (PMC12700832; doi:10.1038/s41587-024-02528-1)
Supplement: Supplementary file 1 — Supplementary Figs. 1–22 and Supplementary Tables 1–3. [file 41587_2024_2528_MOESM1_ESM.pdf]

nature biotechnology

Supplementary information https://doi.org/10.1038/s41587-024-02528-1

Nanocarrier imaging at single-cell resolution across entire mouse bodies with deep learning

In the format provided by the authors and unedited

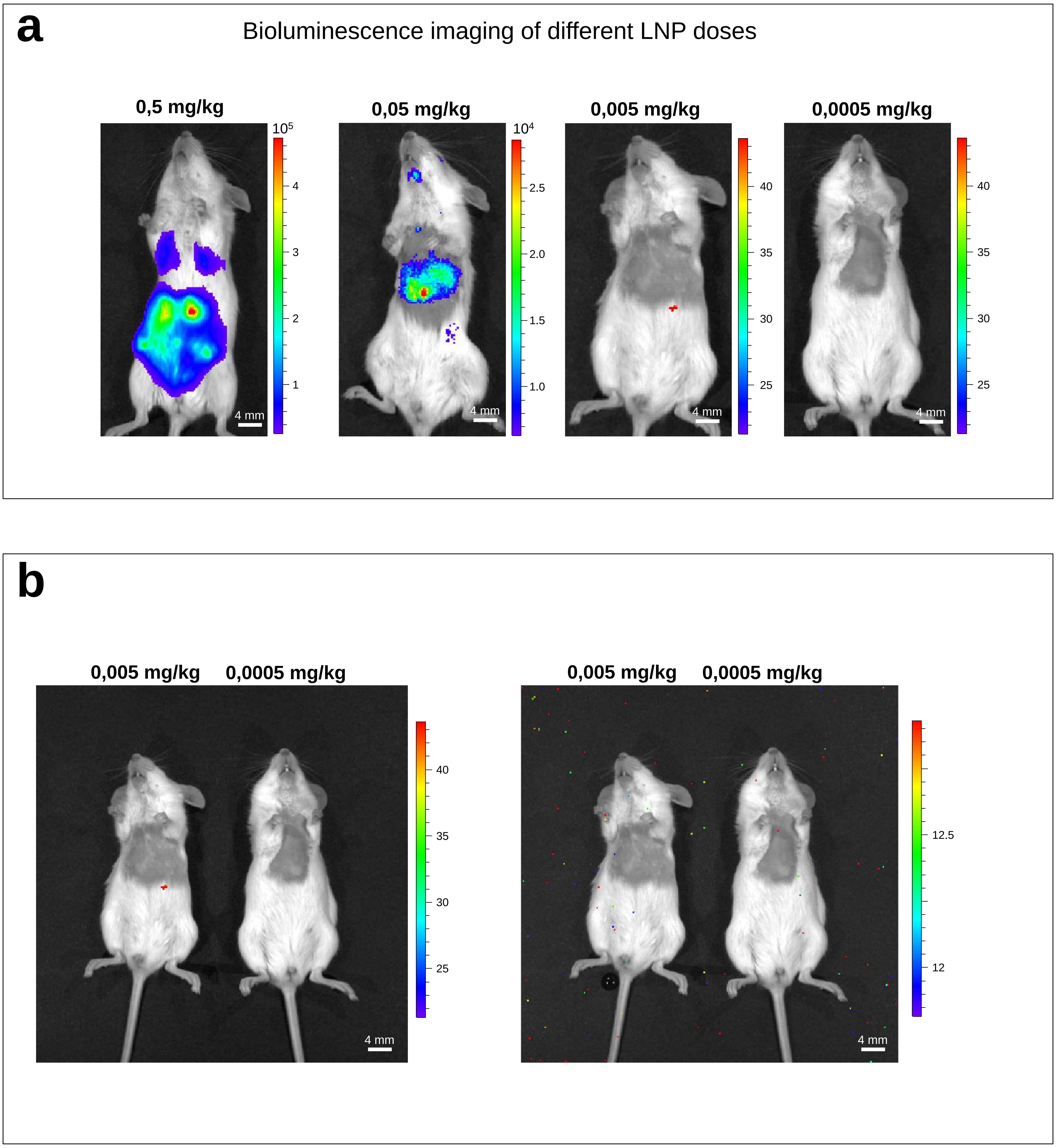

Supplementary Fig. 1: Bioluminescence at Different Doses.

a) Different doses (0.5, 0.05, 0.005, and 0.0005 mg/kg) of LNP-Luc mRNA were administered intravenously into mice. Bioluminescence was measured using the IVIS Lumina II Imaging System (Caliper Life Sciences).

b) The sensitivity of the IVIS Lumina II Imaging System is limited. Doses of 0.005 and 0.0005 mg/kg were analyzed using the same scale bar, ranging from 25-40 (left panel), which already reached the machine's limitation. Additionally, we used a scale bar ranging between 12-13 for these two doses, and we observed significant autofluorescence outside the mouse body (right panel).

Unoptimized DISCO	Refined DISCO
PBS washing	PBS washing
Decolorization 25% Cubic solution with urea (2 days)	Decolorization 25% Cubic solution without urea[1] (1 day)
PBS washing	PBS washing
Decalcification with 10% EDTA pH 8	Decalcification with 10% EDTA pH 8
PBS washing	PBS washing
Nanobody labeling in permeabilization solution with sodium azide (under active pumping)	Nanobody labeling in Permeabilization solution without sodium azide[2] (under active pumping) (This step is only for EGFP or spike protein labeling. Fluorescently labeled LNP-mRNA should skip this step.)
Nanobody labeling in permeabilization solution with sodium azide (under passive shaking)	none
Washing solution	none
PBS washing	PBS washing
50% THF, 70% THF, 90% THF, 100% THF, 100% THF	70% THF, 90% THF, 100% THF, 100% THF
DCM (3-6hrs)	DCM (20mins)[3]
BABB	BABB

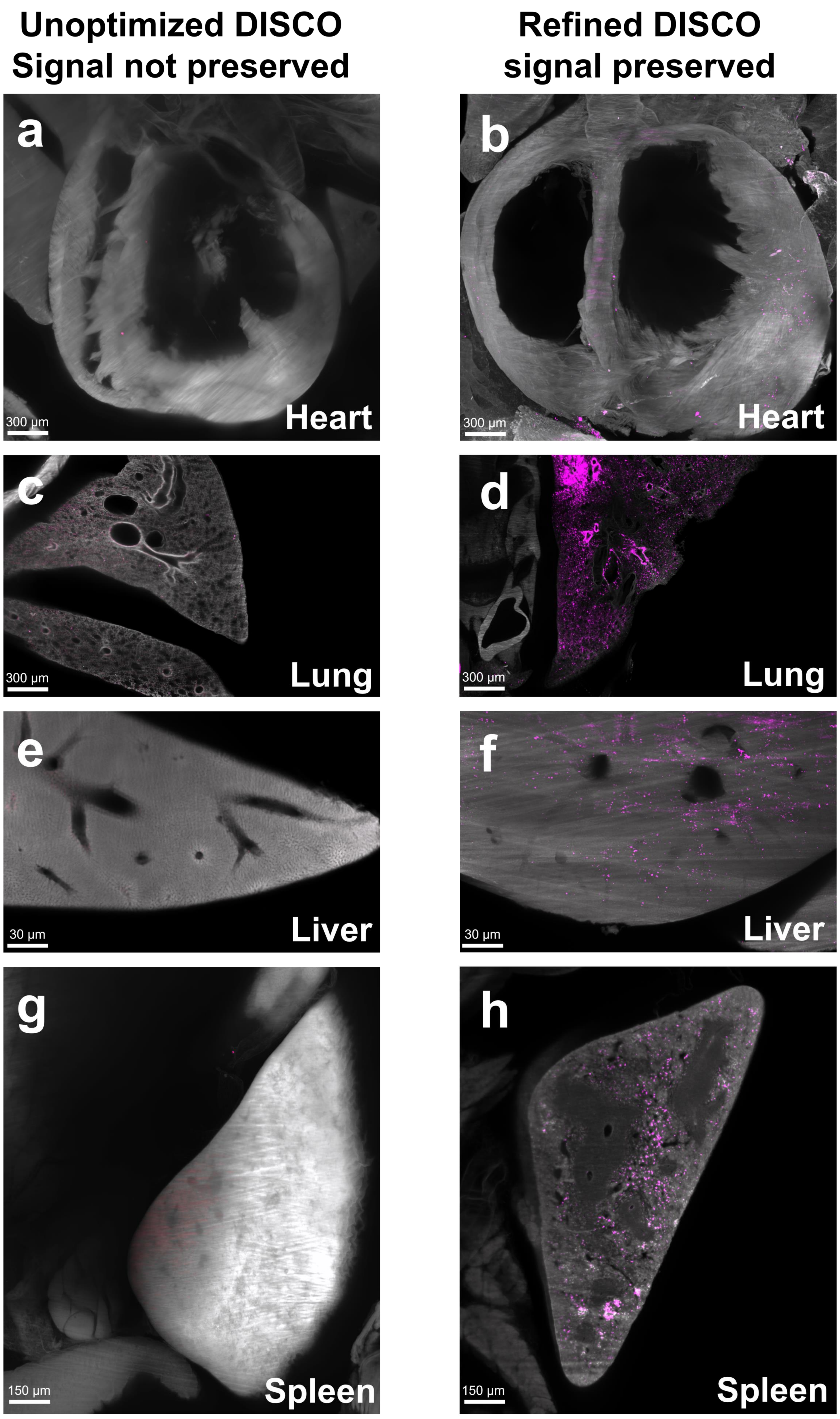

Supplementary Fig. 2: Optimized DISCO clearing preserves the signal.

The unoptimized DISCO and refined DISCO are shown in the table. [1]The CUBIC solution contains urea, which can quench fluorescent signals (https://www.nature.com/articles/s41467-020-15906-5). [2]Permeabilization solution contains sodium azide, which can also quench fluorescent signals (https://link.springer.com/article/10.1007/s10895-019-02398-w). [3]By reducing the DCM incubation time to 20 minutes, it is possible to achieve highly cleared mice (without blurriness) while also preserving the signals.

Compared to the previous version, the refined DISCO lacks urea and sodium azide, and requires less incubation time with DCM in the clearing process. Compared to unoptimized DISCO, the signals in the heart **(a)**, lung **(c)**, liver **(e)**, and spleen **(g)** are weaker. However, our refined DISCO clearing method can preserve the signal in the heart **(b)**, lung **(d)**, liver **(f)**, and spleen **(h)**.

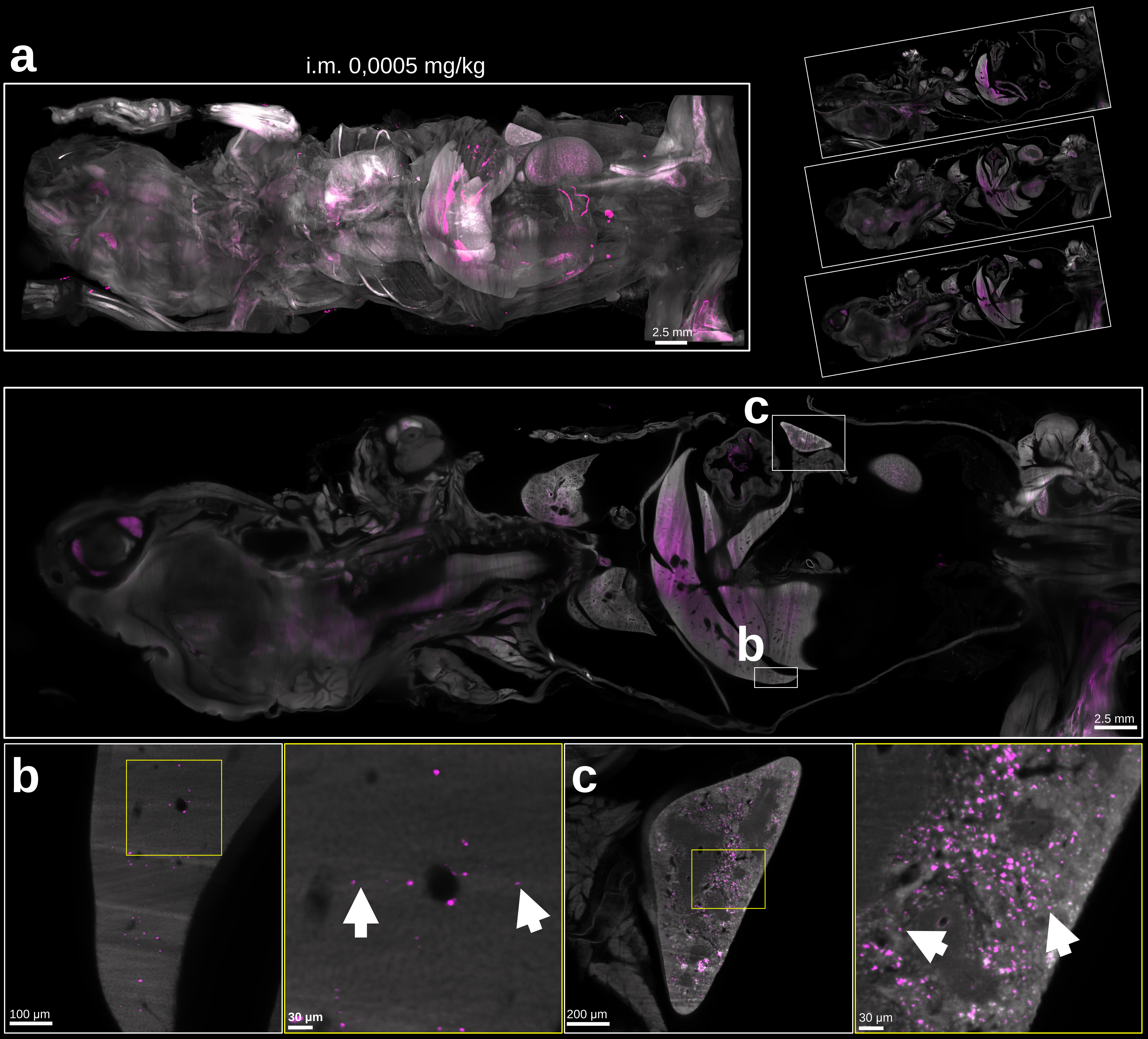

Supplementary Fig. 3: Single-cell resolution imaging throughout the entire mouse body.

0.0005 mg/kg LNP was administered i.m. into mice, followed by cell-level imaging throughout the entire mouse body **(a)**. Different slices were examined, and we selected the liver **(b)** and spleen **(c)** from a single slice to visualize the cell-level resolution of LNP distribution (white arrows).

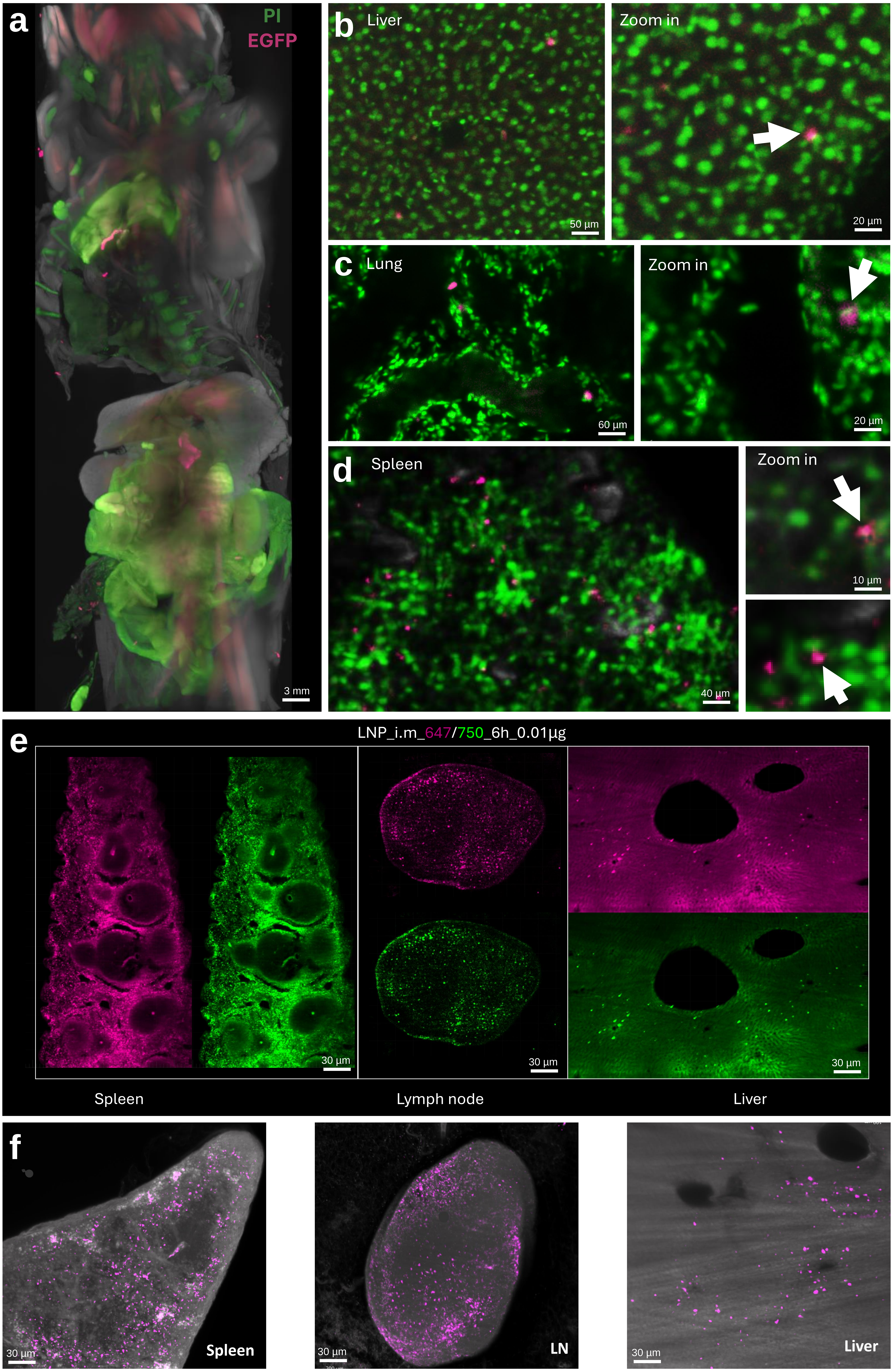

Supplementary Fig. 4: Double staining of Propidium Iodide (PI) and EGFP protein expressed from LNP-mRNA.

a) Whole-body projection; **(b-d)** slice views of different organs, including the liver **(b)**, lung **(c)**, and spleen **(d)**, respectively. The single-cell resolution capabilities of our technology are evident from the overlap of GFP-expressing cells with single PI+ cells, as well as their similar size (white arrows). **e)** LNPs conjugated with Alexa Fluor 750 or Alexa Fluor 647 were injected into the same mouse intramuscularly (i.m.). They exhibited similar distributions in various organs, such as the spleen, lymph nodes, and liver (imaged using light-sheet microscopy). **f)** The control (LNPs conjugated with Alexa Fluor 647 carrying EGFP mRNA) exhibited similar distributions in various organs. Alexa Fluor 647-labeled DMG-PEG was used instead of Alexa Fluor 647-labeled EGFP mRNA.

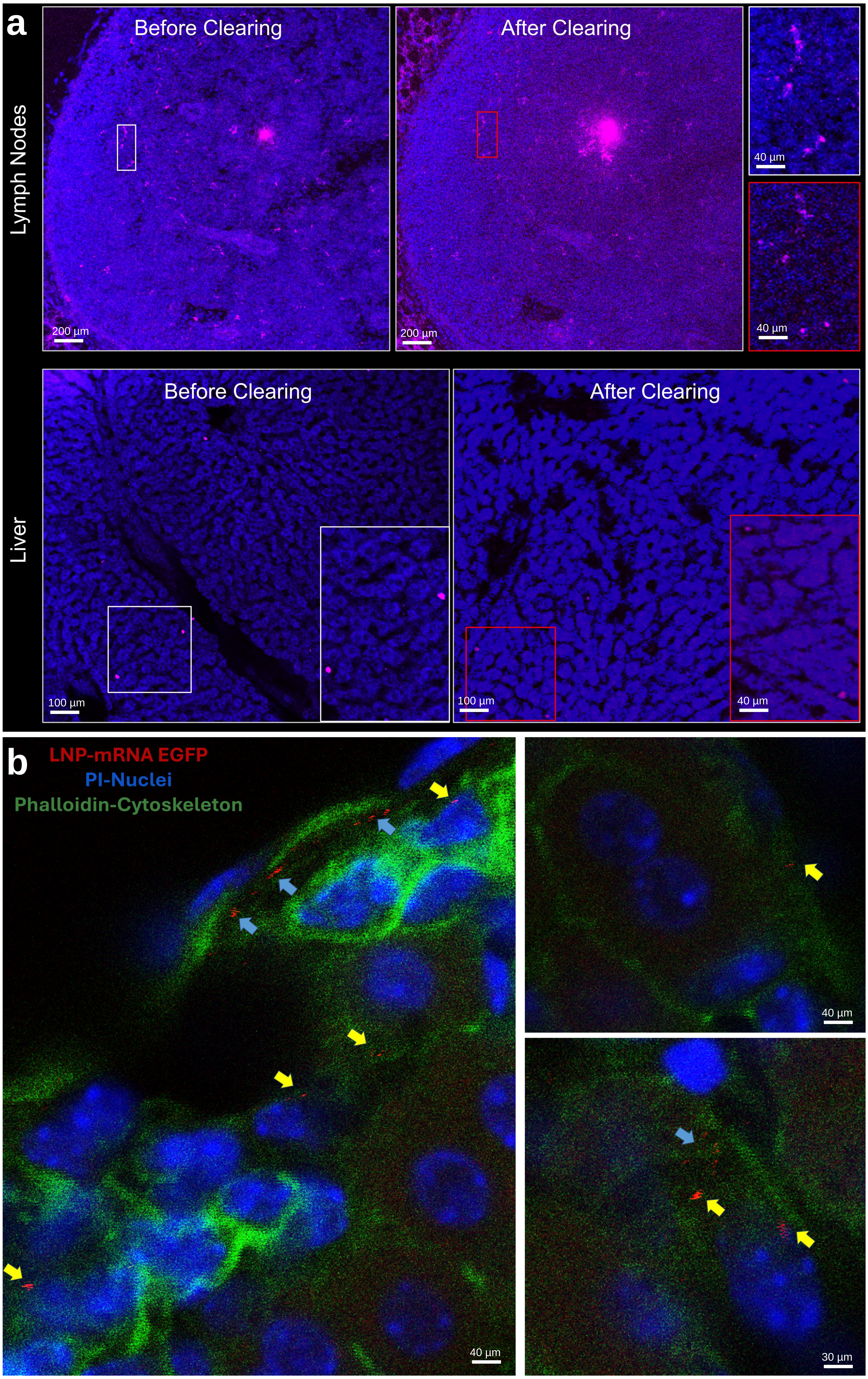

Supplementary Fig. 5: Validation of cleared mouse images via histology.

a) Mice were injected intramuscularly with 0.5 mg/kg LNP EGFP-mRNA. After 72 hours, the mice were perfused, and 500 µm tissue slices from organs such as the liver and lymph nodes were cut using a vibratome. These slices were imaged by confocal microscopy first, then underwent the optimized DISCO clearing process and were imaged again. The LNP-expressed EGFP protein signal was well preserved in the liver and lymph nodes. **b)** The intracellular and extracellular nature of the LNP signals can be obtained in regions of interest after whole-body clearing and imaging of identified regions. LNP-mRNA is labeled with Alexa Fluor 647, PI stains the nuclei, and Phalloidin stains the cytoskeleton in multiple areas of the liver. The yellow arrow indicates the LNPs inside the cells (cytoplasm), and the blue arrow indicates the LNPs outside the cells (extracellular matrix). Our technology does not wash out the nanoparticles located outside the cells; the nanoparticles remain in the extracellular matrix.

Polyplexes, based on branched polyethyleneimine (PEI)

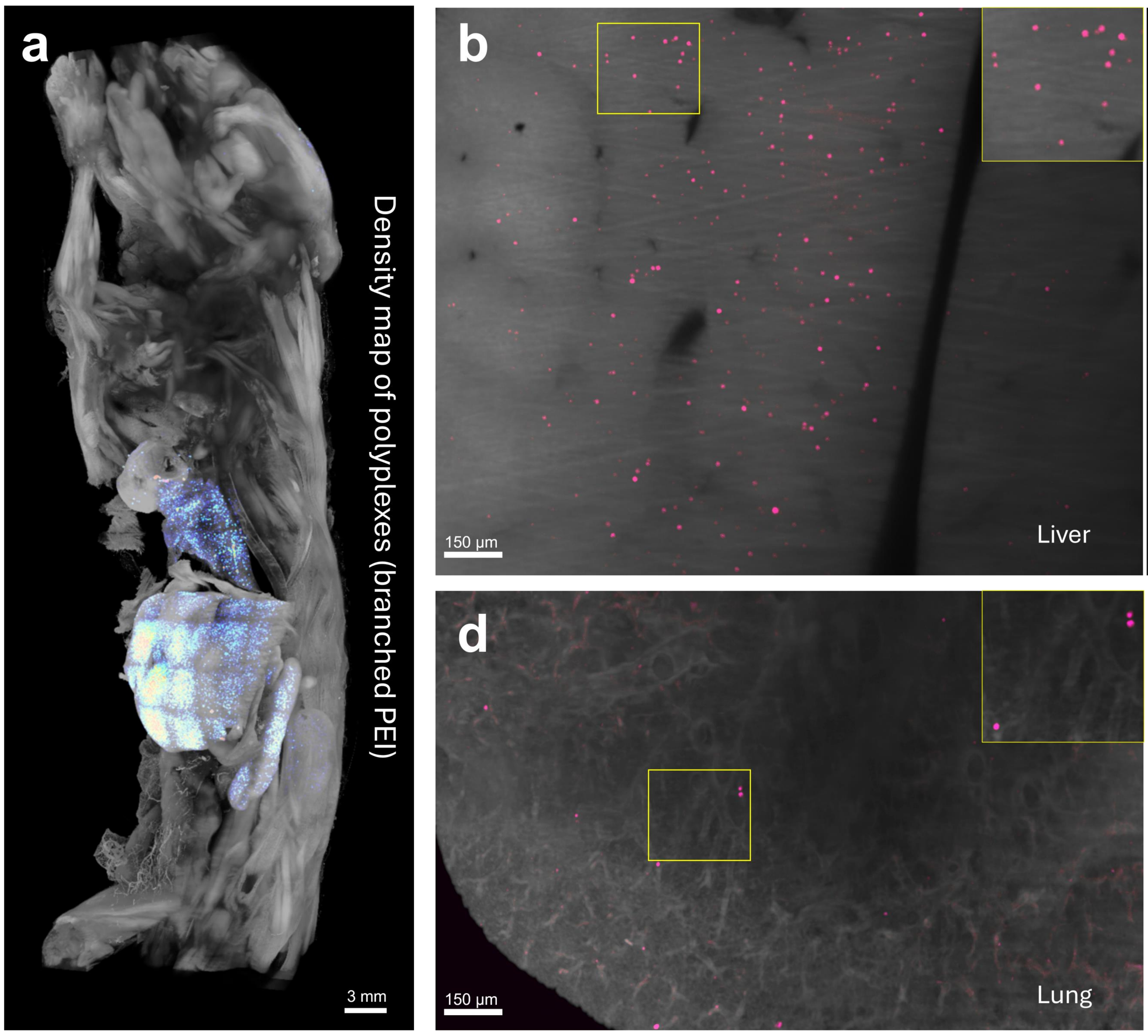

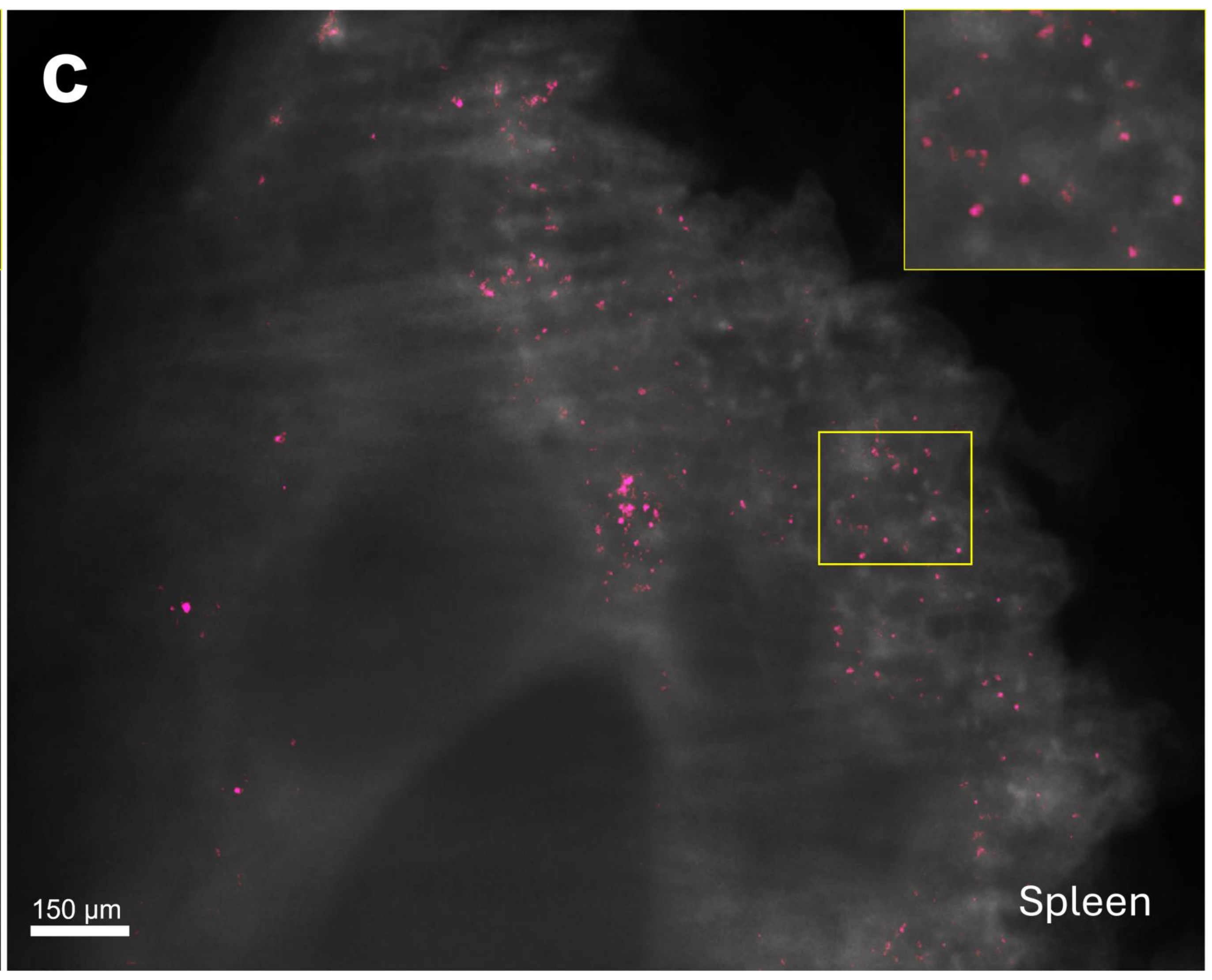

e

SCP-Nano Quantification

Organ	Number of Branched PEI
head	10,122
heart	125,800
lung	338,910
liver	3,725,414
spleen	429,821
kidneys	37,735
**all**	**4,667,802**

liposomes

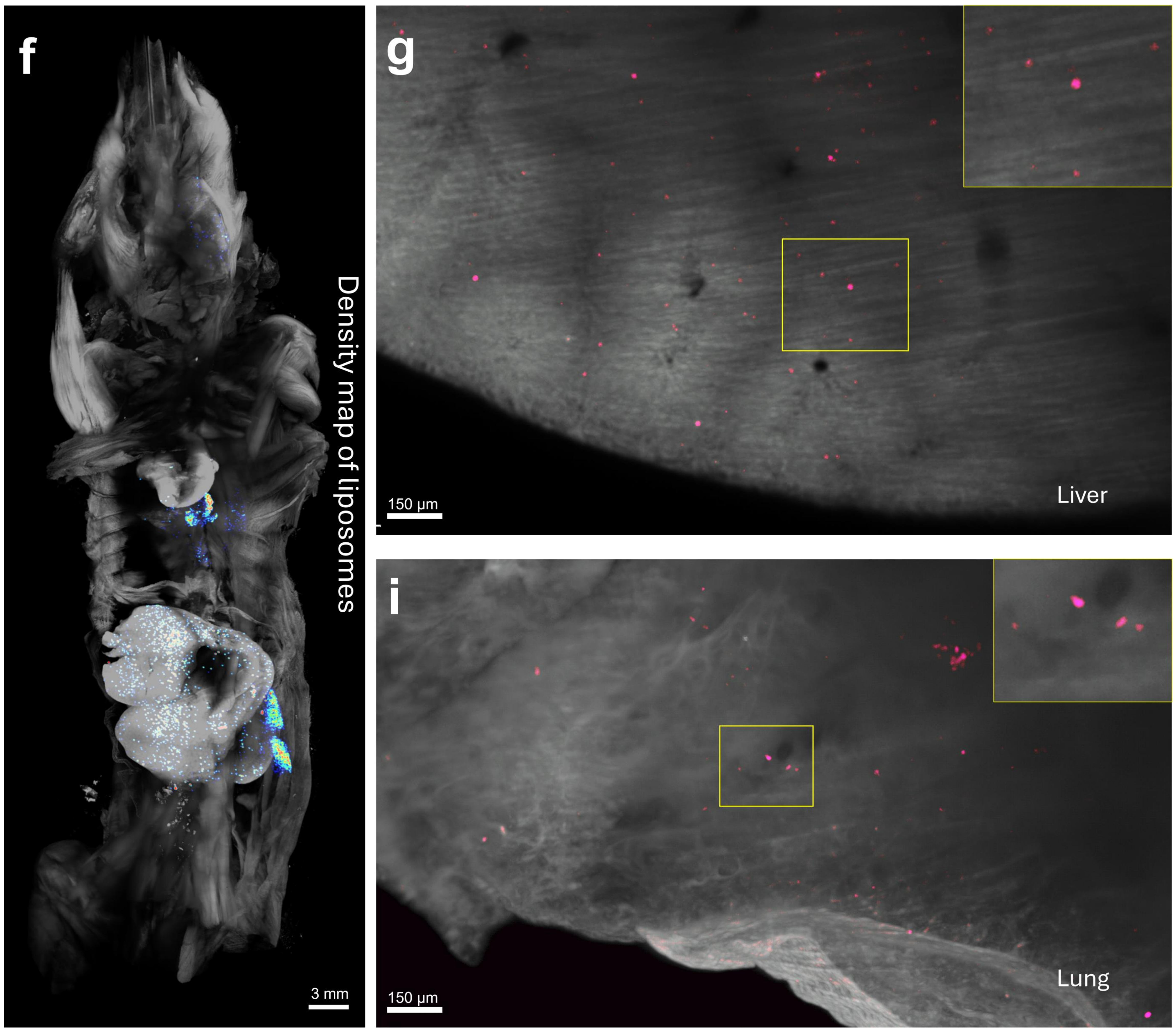

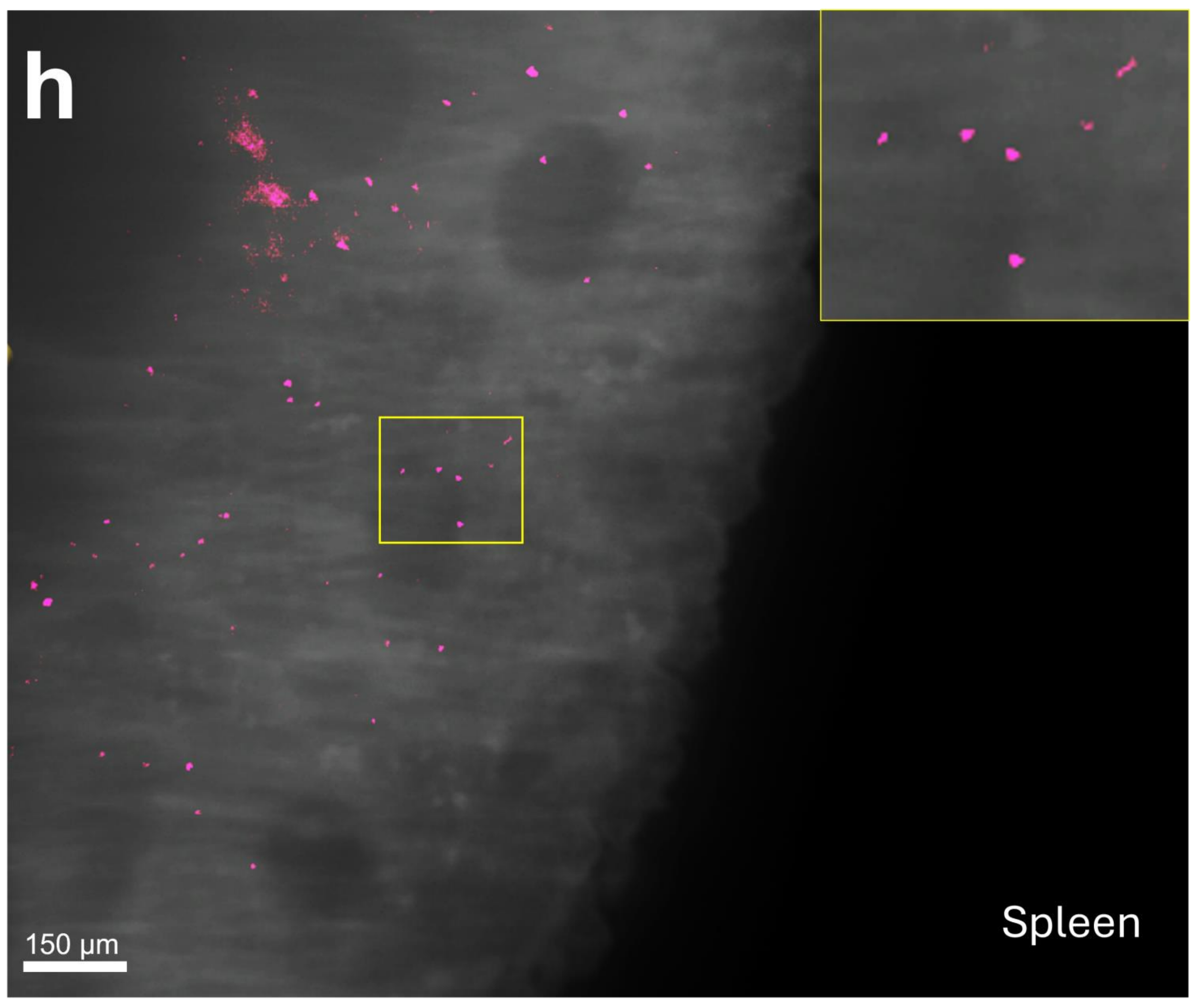

j

SCP-Nano Quantification

Organ	Number of Liposomes
head	13,725
heart	1,650
lung	75,676
liver	286,062
spleen	213,177
kidneys	14,640
**all**	**604,930**

Supplementary Fig. 6: SCP-Nano can visualize various nanoparticle delivery systems.

Branched polyethyleneimine (PEI) delivers single-stranded Alexa Fluor 647-labeled DNA. **a)** Cell-level density map of PEI distribution in the whole mouse body. **(b-d)** Slice views of each organ, such as the liver **(b)**, spleen **(c)**, and lung **(d)**, respectively. **e)** SCP-Nano quantifications for major organs showing PEI distribution. Liposomes based on the FDA-approved drug Doxil deliver Atto 647 dye. **f)** Cell-level density map of liposome distribution in the whole mouse body. **(g-i)** Slice views of each organ, including the liver **(g)**, spleen **(h)**, and lung **(i)**, respectively. **j)** SCP-Nano quantifications for major organs showing liposome distribution.

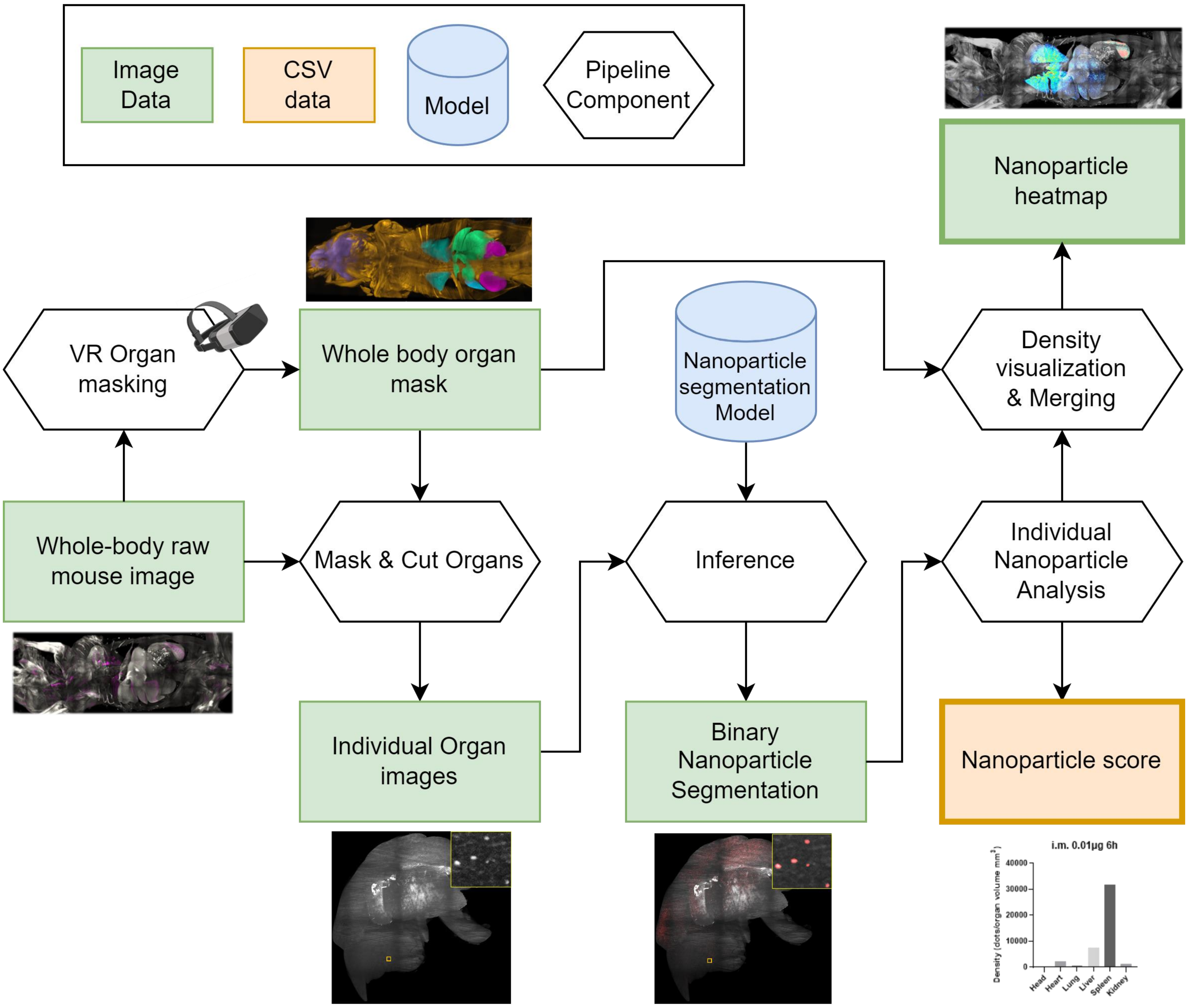

Supplementary Fig. 7: Detailed illustration of SCP-Nano pipeline

VR glasses were used for organ annotations and SCP-Nano algorithms to detect nanocarrier dots in patches. After achieving higher prediction accuracy, we applied SCP-Nano algorithms to detect whole-body nanocarrier distributions. Furthermore, we also generated a cell-level density map showing LNP distribution throughout the entire mouse body.

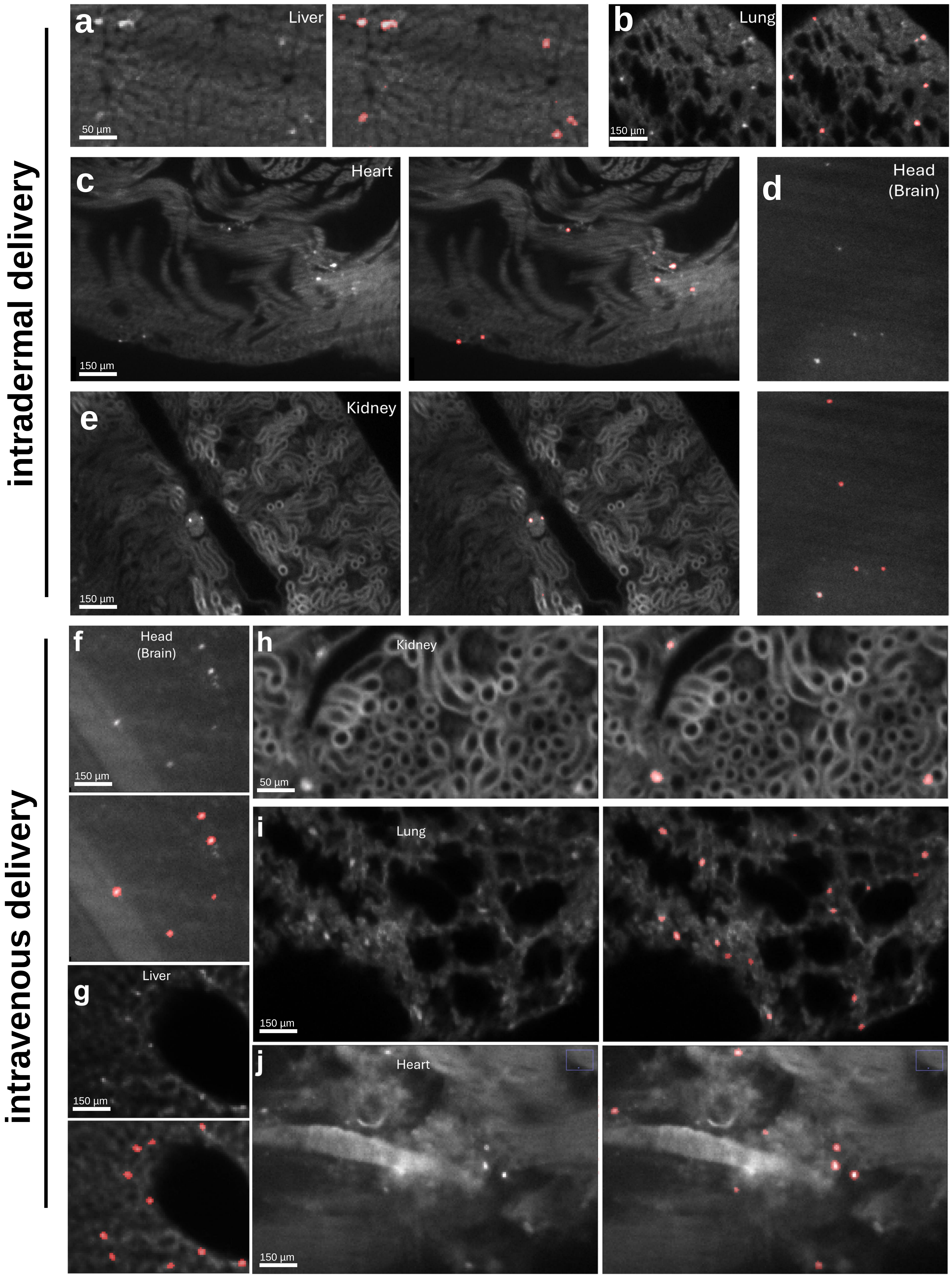

Supplementary Fig. 8: Single-cell resolution in both raw imaging data (gray dots) and segmentation data (red dots) generated by SCP-Nano.

(a-e) the distribution of intradermal injection of 0.0005 mg/kg LNP-EGFP mRNA. Slice views of different organs, including the liver **(a)**, lung **(b)**, heart **(c)**, head **(d)**, and kidney **(e)**, respectively. **(f-j)** the distribution of intravenous injection of 0.0005 mg/kg LNP-EGFP mRNA. Slice views of different organs, including the head **(f)**, liver **(g)**, kidney **(h)**, lung **(i)**, and heart **(j)**, respectively.

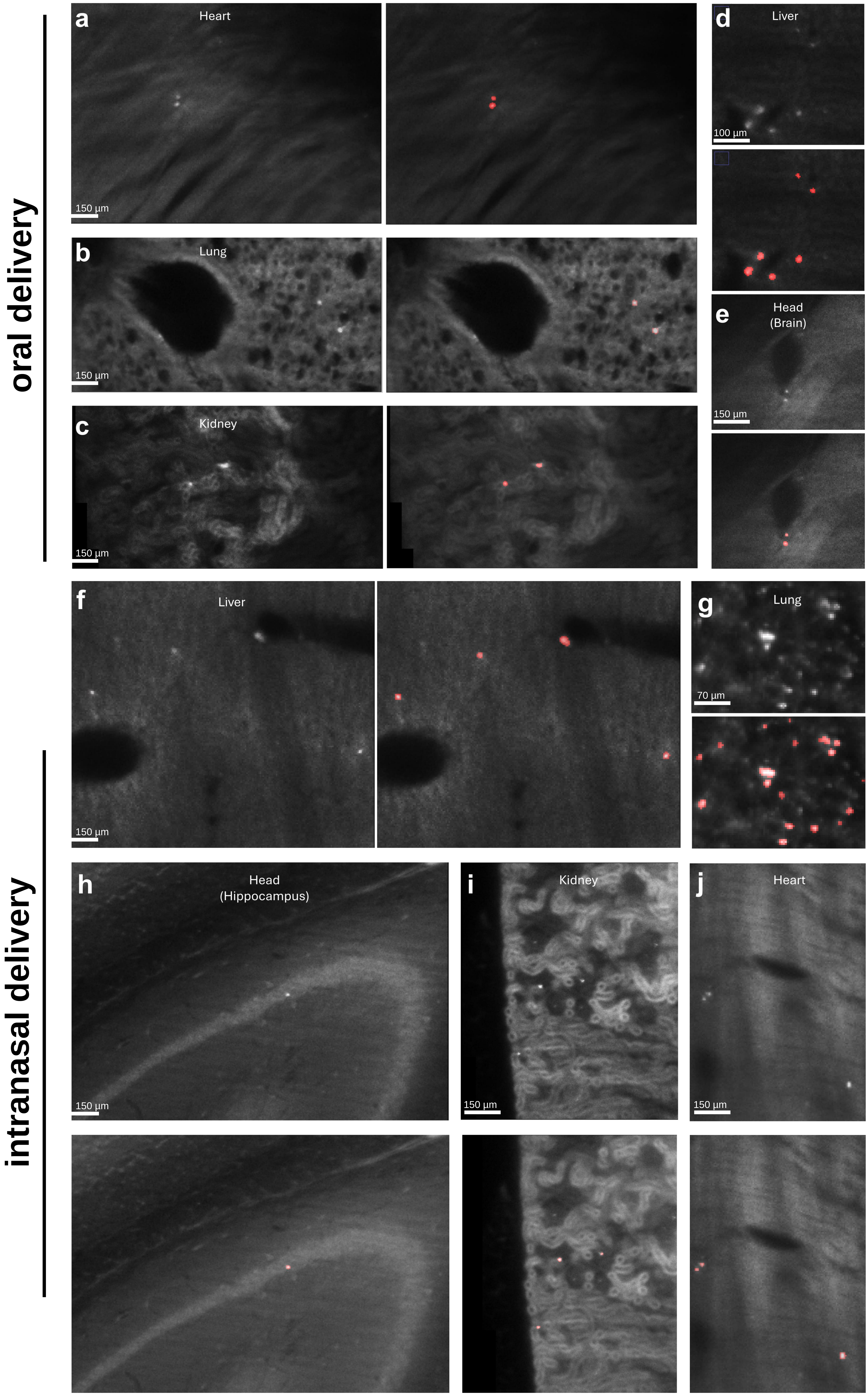

Supplementary Fig. 9: Single-cell resolution in both raw imaging data (gray dots) and segmentation data (red dots) generated by SCP-Nano.

(a-e) the distribution of oral injection of 0.0005 mg/kg LNP-EGFP mRNA. Slice views of different organs, including the liver **(a)**, lung **(b)**, head **(c)**, kidney **(d)**, and heart **(e)**. **(f-j)** the distribution of intranasal injection of 0.0005 mg/kg LNP-EGFP mRNA. Slice views of different organs, including the heart **(f)**, lung **(g)**, kidney **(h)**, liver **(i)**, and head **(j)**.

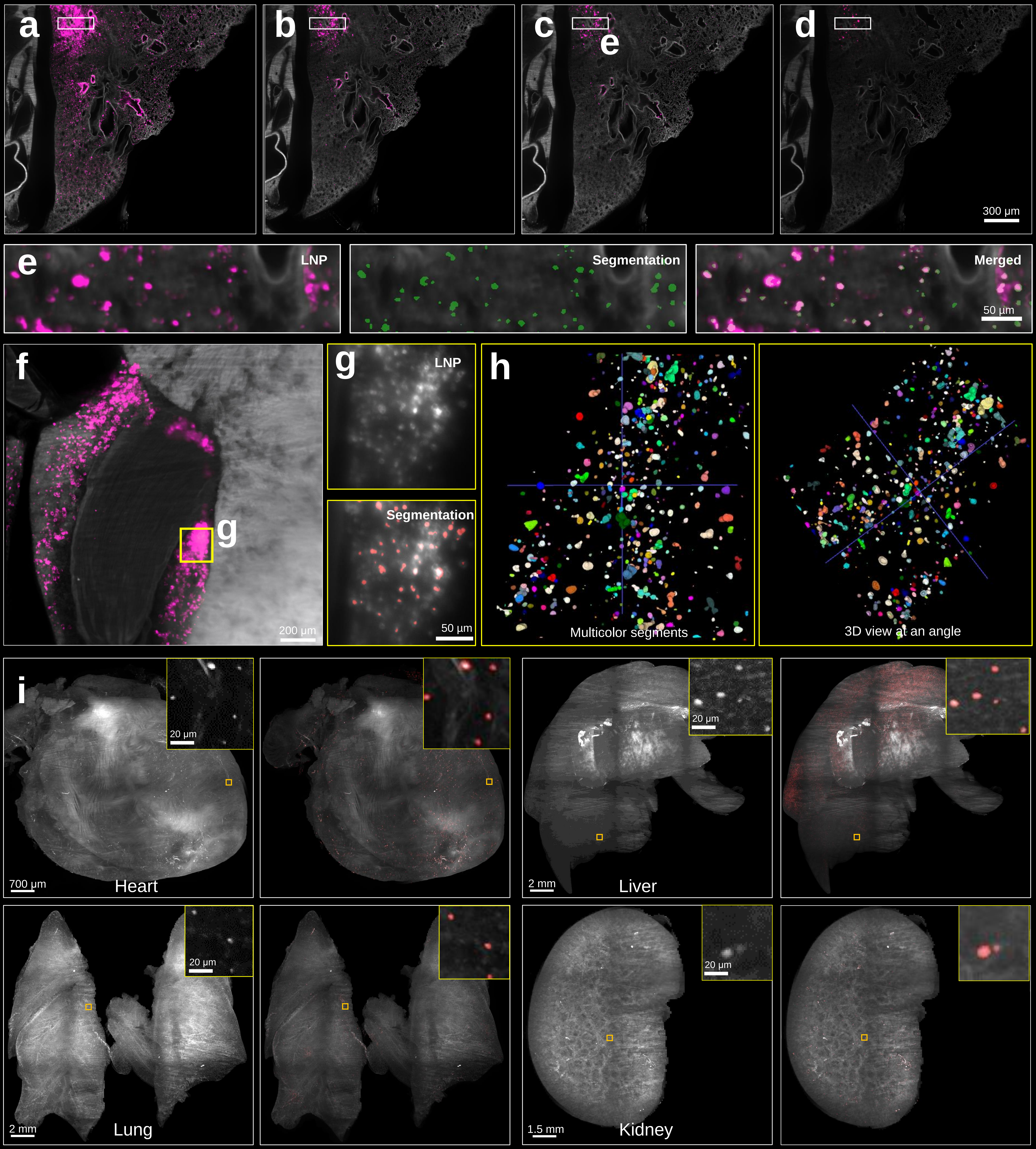

Supplementary Fig. 10: Single-cell resolution imaging in saturated “crowded” signal regions.
8-bit projection images inevitably present regions with saturated signal, in which single cells are not visible. To demonstrate the preservation of single-cell resolution, **a-d)** we show threshold changes to highlight the dense areas of LNP distribution in the lung for the intranasal injection route, where individual cells gradually become visible in the same images. **e)** However, the algorithms can faithfully identify signals as they do not rely solely on signal intensity at one threshold. LNP raw images and SCP-Nano segmentation images are well colocalized, demonstrating single-cell segmentation at this specific threshold. **f, g)** show threshold changes to highlight the dense areas of RetroAAV distribution near the ureter. **h)** SCP-Nano multicolour segmentation images demonstrate single-cell segmentation. **i)** Representative examples of LNP segmentation in different organs. Original image of LNP on the left, segmentation overlaid in red on the right.

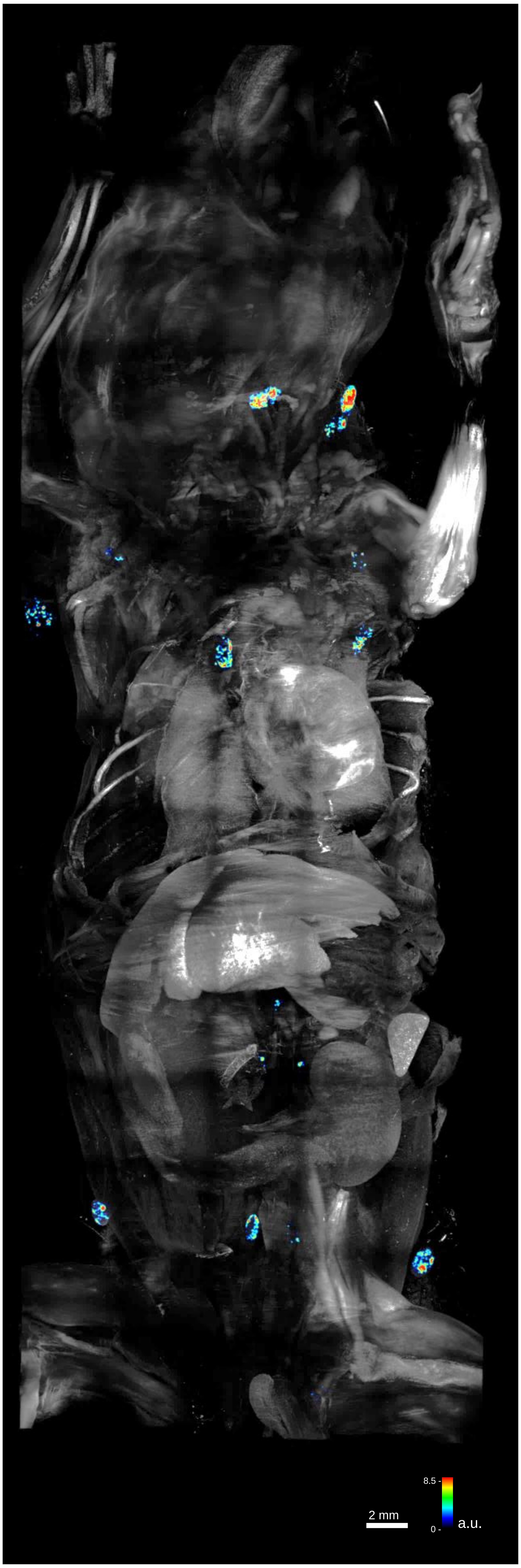

Supplementary Fig. 11: LNP distribution in lymph nodes throughout the mouse body.

SCP-Nano generates a cell-level resolution density map of LNP distribution in the whole-body lymph nodes.

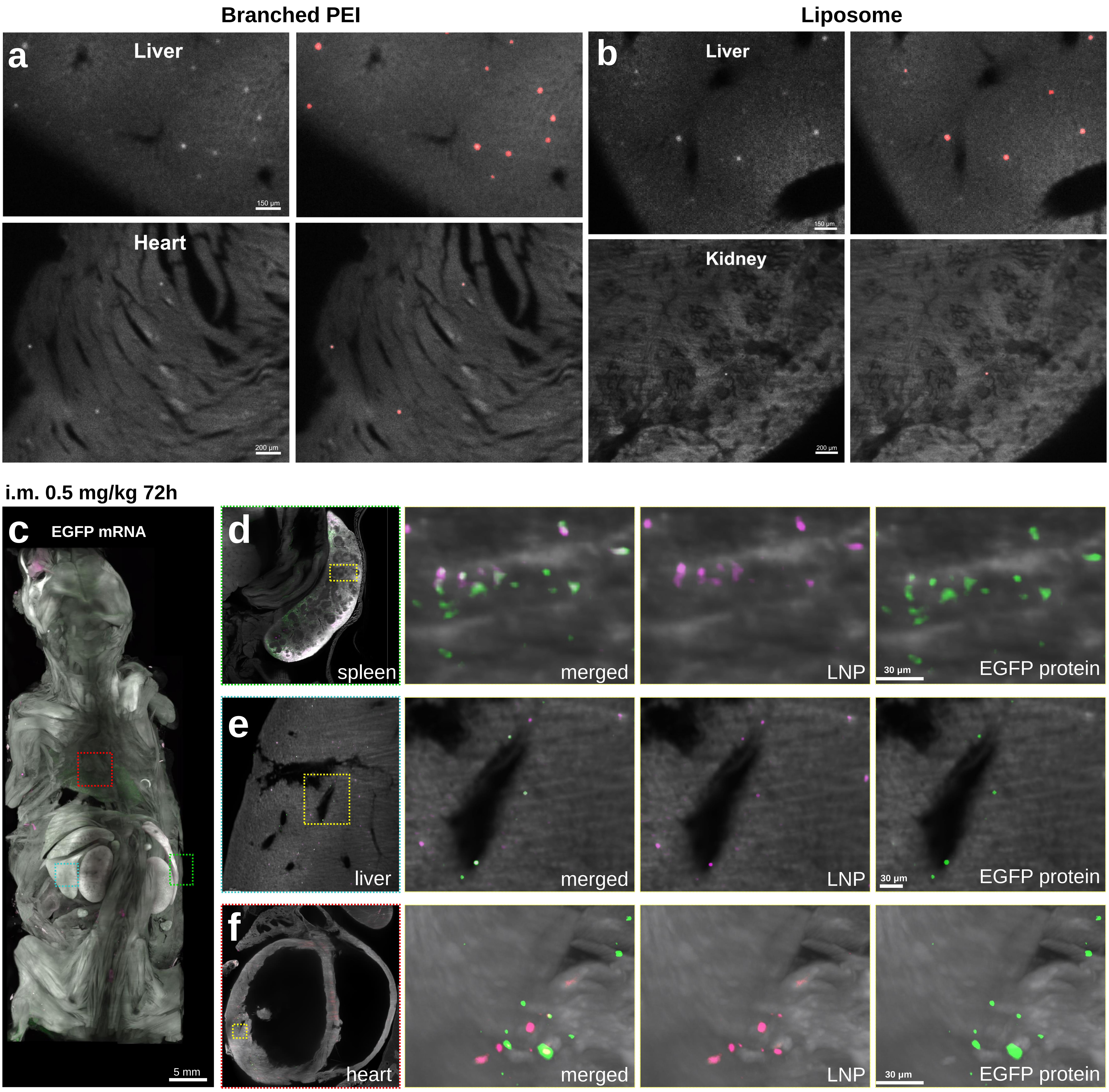

Supplementary Fig. 12: SCP-Nano segmentation of cells marked by fluorescently labeled mRNA and protein expression from LNP-delivered mRNA.

Segmentation by SCP-Nano for branched PEI and liposomes. Representative images of branched PEI distribution in the liver and heart **(a)**. Representative images of liposome distribution in the liver and kidney **(b)**. **c-f)** SCP-Nano reveals LNP-mRNA expressions. **c)** Whole-body distribution of LNP-EGFP mRNA and protein expression 72 hours post-injection using the dose of 0.5 mg/kg via the i.m. route. The whole-body projection view includes various organs and zooms into cell-level resolution, such as the spleen **(d)**, liver **(e)**, and heart **(f)**.

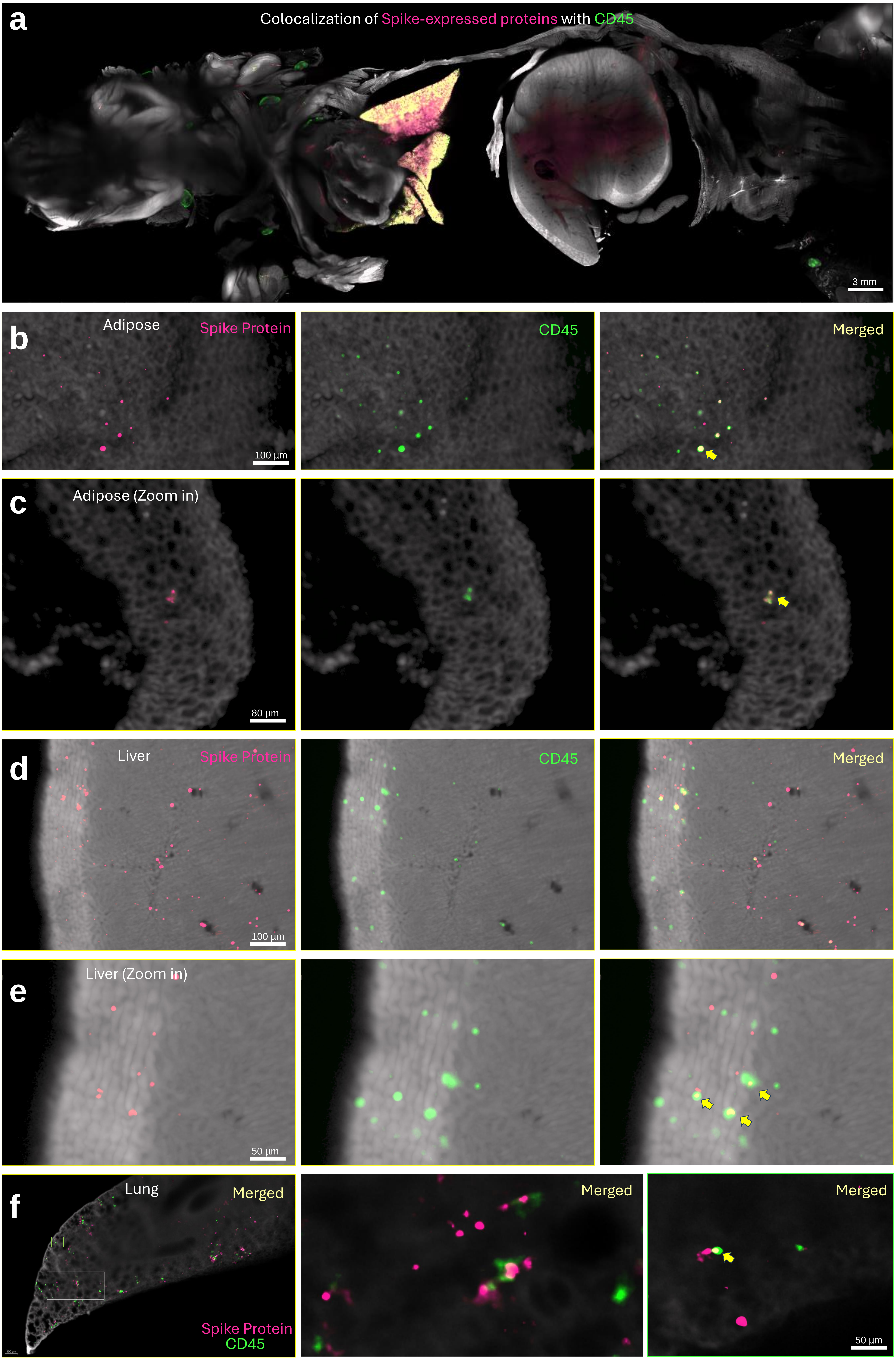

Supplementary Fig. 13: Double staining of Spike-expressed protein (magenta) and CD45-labeled immune cells (green) in the whole mouse body for cell type identification using wildDISCO method.

The yellow arrow indicates the colocalization of the LNP-mRNA-expressed spike protein in CD45+ immune cells, 72 hours post-injection. **a)** Whole-body projection; **(b-f)** slice views of different organs or tissues, including adipose tissue **(b,c)**, liver **(d,e)**, and lungs **(f)**.

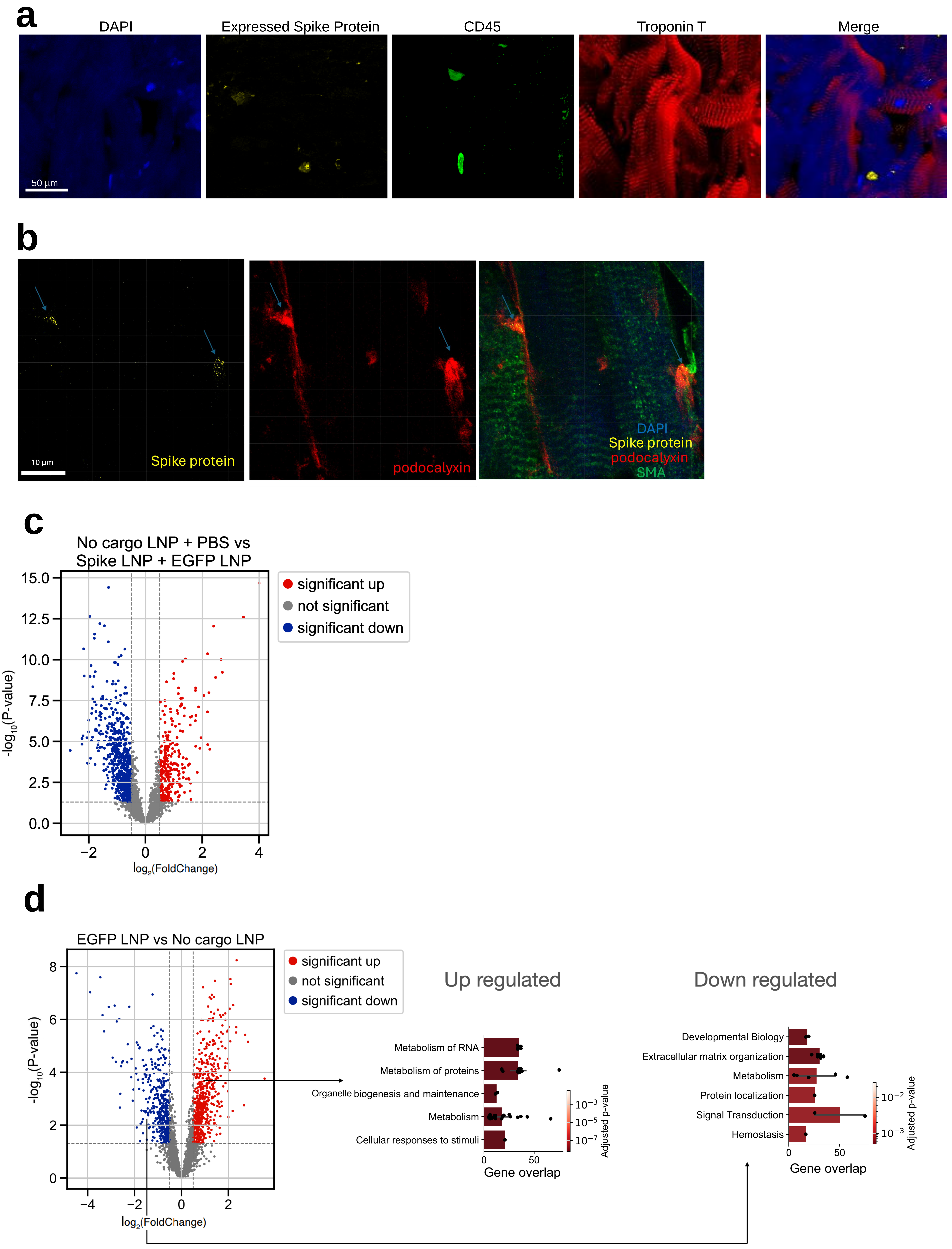

Supplementary Fig. 14: Co-staining of LNP-mRNA derived spike protein with different cell types in the heart and proteome differences between controls and mRNA carrying LNP injections.

Co-staining of LNP with cardiomyocytes using Troponin T and with immune cells using CD45 **(a)**. Co-staining of LNP with capillary endothelial cells using podocalyxin and with arteries using alpha-SMA **(b)**. Pathway analysis for No cargo LNP + PBS vs. Spike LNP + EGFP LNP **(c)** and EGFP LNP vs. no cargo LNP **(d)**. n=9, mean ± s.d.; (one-way analysis of variance).

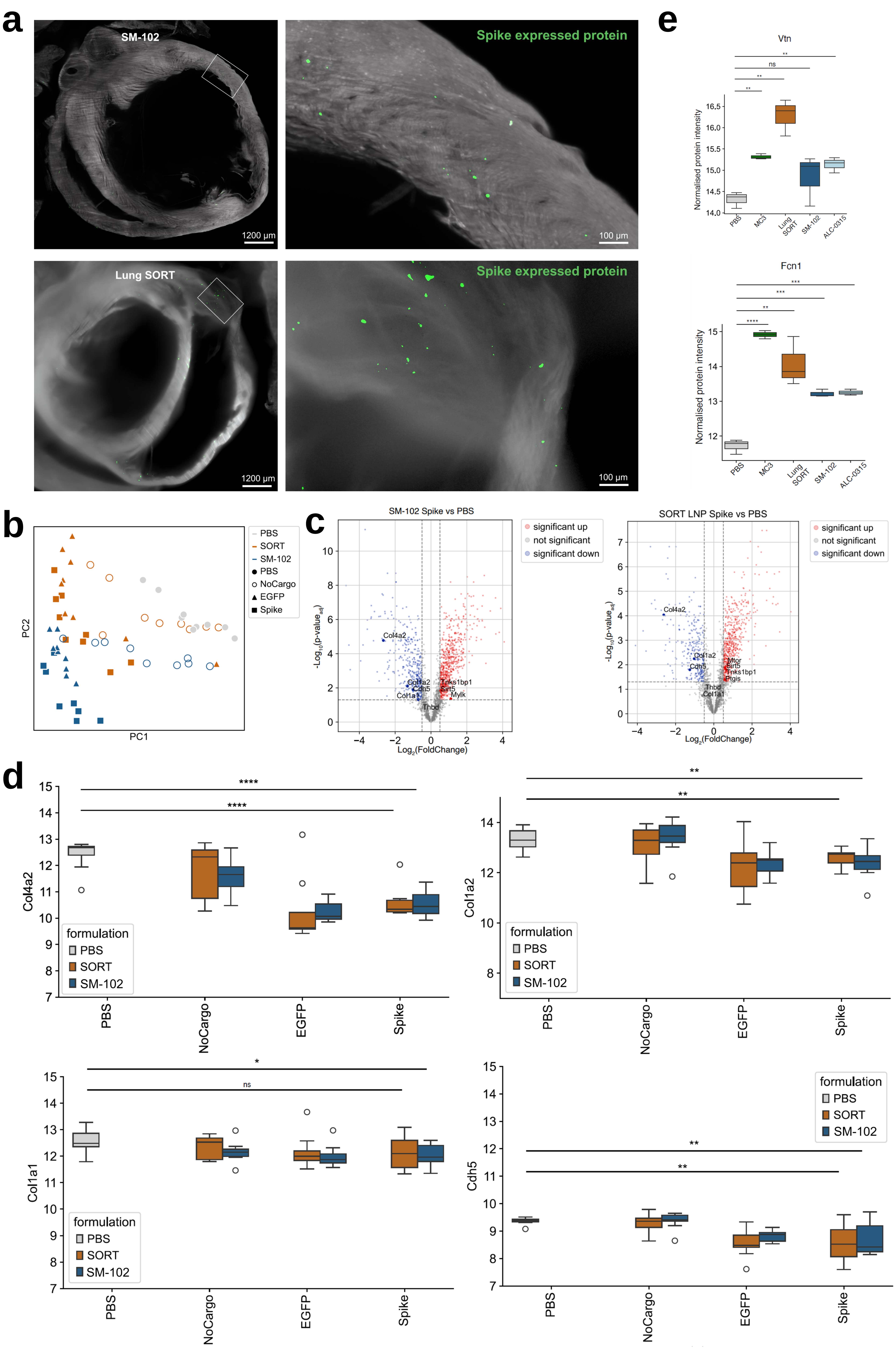

Supplementary Fig. 15: Heart off-targeting off SM-102 and Lung SORT formulations.

After 72 hours following intramuscular injection of SM-102 and Lung SORT formulations with spike mRNA, we sacrificed the mice, cleared the tissues, and obtained heart imaging to observe the distribution of Spike-expressed proteins. **a)** SM-102 formulation: Spike-expressed proteins (green) in the heart. Lung SORT formulation: Spike-expressed proteins (green) in the heart. **(b-d)** Proteomics analysis of SM-102 and SORT formulations in heart tissue. PCA plot **(b)** and volcano plots **(c)** show that the SM-102 and SORT groups are well separated compared to the PBS group. **(d)** Several vascular-based protein markers in Spike and EGFP groups, such as Col4a2, Col1a1, Col1a2, and Cdh5 (VE-Cadherin), are decreased compared to the PBS and the non-cargo groups. **e)** Incubation of particles with mouse plasma at 37°C, followed by (sucrose cushion-based) size separation to enrich nanoparticle-bound proteins. After analyzing the MS-based proteomics data, Vitronectin (Vtn) and Ficolin-1 (Fcn1) were found in the protein corona of all formulations. Compared to the PBS groups, the most known LNP formulations had higher amounts of Vitronectin and Ficolin-1. ns: $P > 0.05$, *: $P < 0.05$, **: $P < 0.01$, ***: $P < 0.001$, ****: $P < 0.0001$. n=9. (one-way analysis of variance).

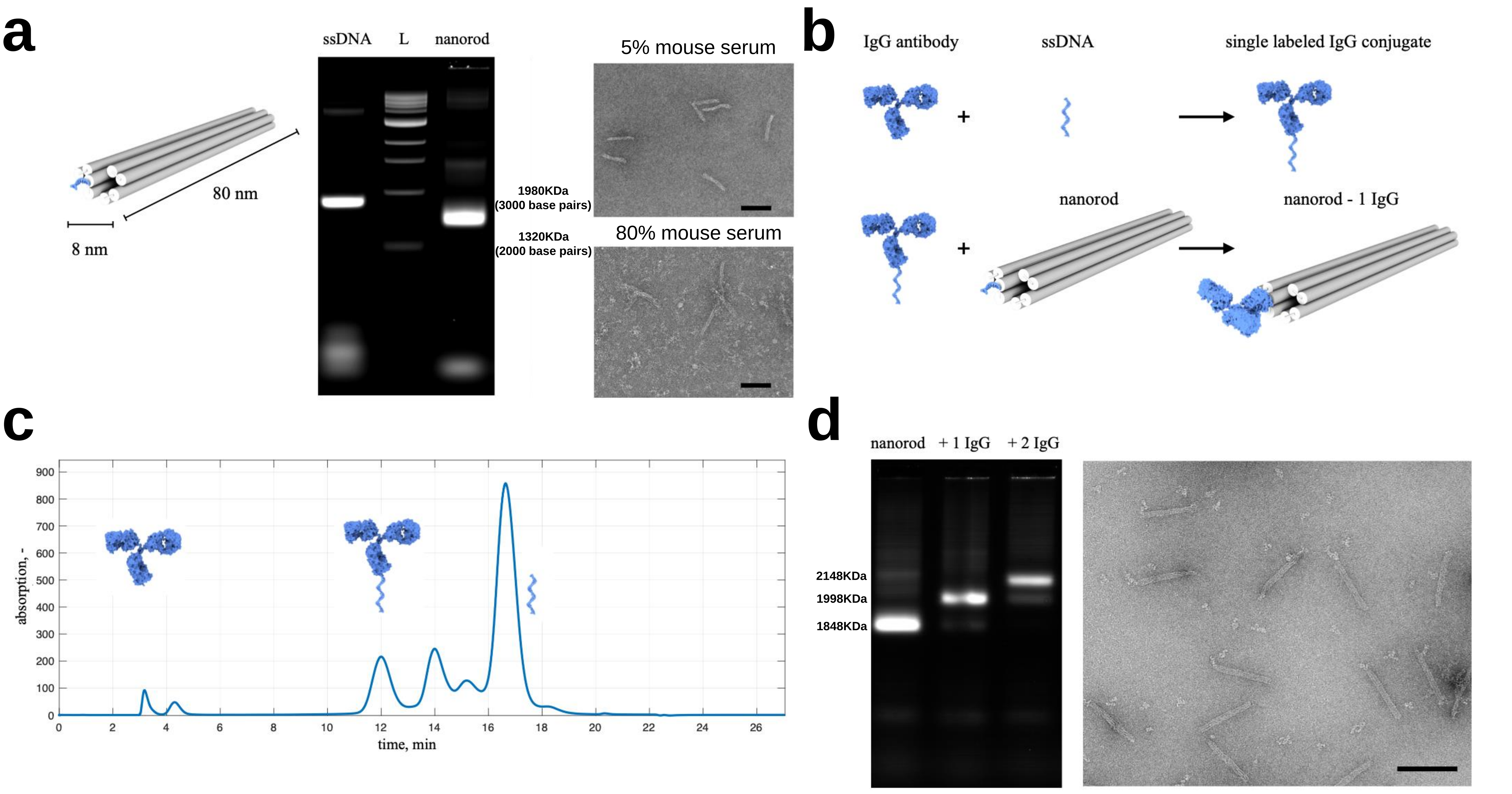

Supplementary Fig. 16: Antibody conjugation and production of DNA origami nanorod.

a) left: Schematic representation of DNA origami nanorod used in in vivo studies. middle: Laser scanned image of 2 % agarose gel with from left to right the cleaved phagemid ssDNA yielding scaffold and staple strands, a 1kb ladder as reference and the folded nanorod. right: negative stain transmission electron microscope (TEM) micrograph of the nano rod stabilized with PEG-polylysine and incubated in mouse serum (5% (top), 80% (bottom), at 37°C, 1h, 50 fold diluted for grid preparation) (scale bar 100 nm). **b)** Schematic representation of attaching single stranded DNA to IgG antibody and hybridization to nanorod using single stranded adapter protruding from a helix end. **c)** Purification of the coupling reaction of IgG antibody and single-stranded ssDNA via anion exchange chromatography. **d)** left: Agarose gel on which the nanorod with 0 to 2 protruding single stranded handle adapters, incubated with IgG modified with one ssDNA strand (1.5 x excess over binding site), was electrophoresed (po, pocket). right: TEM micrograph of nanorod with one IgG antibody attached (scale bar 100 nm).

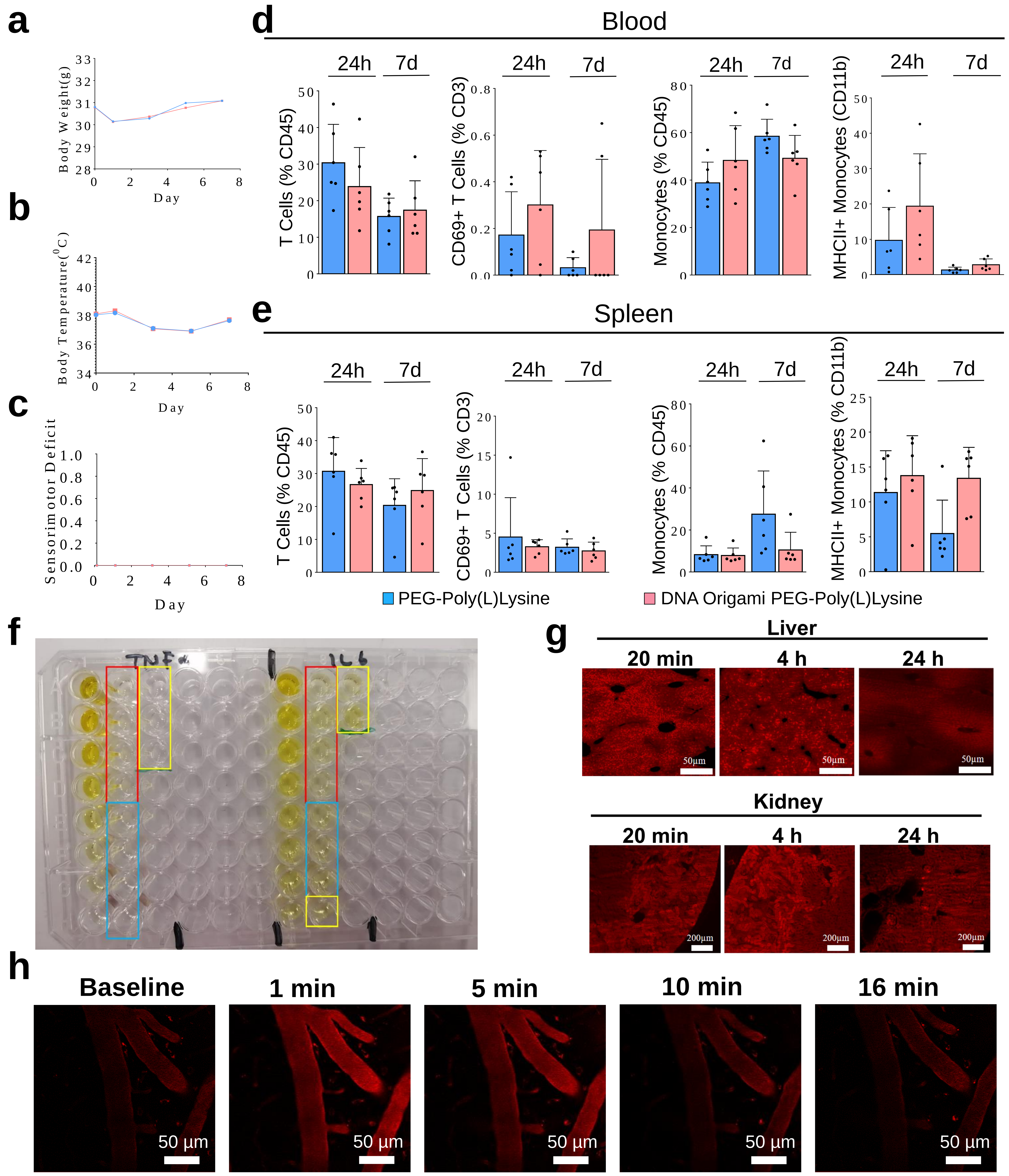

Supplementary Fig. 17: Immune parameters and in vivo clearance.

Effects of DNA origami and PEG-Poly(L)Lysine control, administered by gavage on **a)** body weight, **b)** body temperature and **c)** behavioral score (on day 0, 1, 3, 5 and 7). Comparison of monocytes and T cell populations and activation in **d)** blood and **e)** spleen after DNA origami administration (Data are presented as mean ± SD). n=3. **f)** To assess cytokine release following DNA origami injection, we collected blood serum samples 24 hours post-administration from animals injected with non-targeting DNA, immune-targeting DNA origami, and from control animals. The results show TNFα and IL-6 release in different groups: non-targeting DNA origami-injected animals (indicated by a red rectangle), immune-targeting DNA origami-injected animals (indicated by a blue rectangle), and the control group (indicated by a yellow rectangle). **g)** In vivo stability of DNA origami. Distribution of DNA origami in the liver and kidneys at 20 minutes, 4 hours, and 24 hours after femoral vein injection. **h)** Blood clearance of naked DNA origami by intravital 2-photon imaging of the pial vessels after intravenous injection of non-targeting DNA origami (red) for 16 minutes.

a

Untargeted origami	Hydrodiameters / nm	PDI	Zeta potential / mV
Hepes buffer	17.4 ± 0.52	0.12 ± 0.05	-4.1 ± 0.41
70% serum	23.5 ± 0.26	0.20 ± 0.04	-6.8 ± 0.81
90% serum	23.9 ± 0.35	0.16 ± 0.07	-7.1 ± 0.54
99% serum	24.0 ± 0.39	0.18 ± 0.06	-7.2 ± 0.49

b

Immune cell-targeted origami	Hydrodiameters / nm	PDI	Zeta potential / mV
Hepes buffer	21.5 ± 0.29	0.17 ± 0.03	-5.0 ± 0.30
70% serum	26.5 ± 0.52	0.14 ± 0.02	-7.6 ± 0.76
90% serum	26.9 ± 0.24	0.19 ± 0.05	-8.1 ± 0.69
99% serum	26.8 ± 0.38	0.16 ± 0.08	-8.1 ± 0.46

c

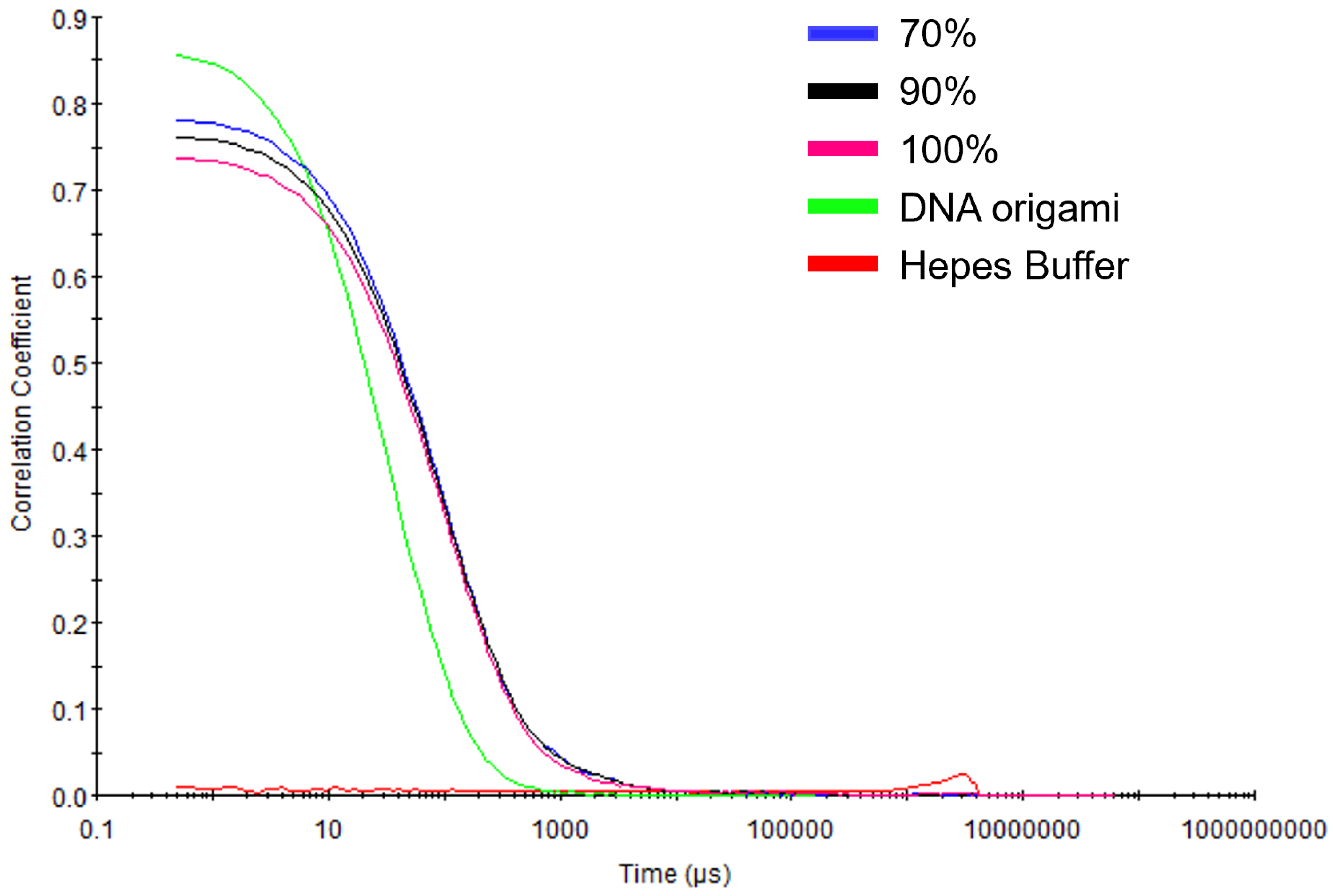

Supplementary Fig. 18: Protein corona of untargeted and targeted origamis.

a) Non-targeted origami was incubated with various serum concentrations (70%, 90%, and 99%) for 5 minutes and then measured using Dynamic Light Scattering (DLS). **b)** Immune cell-targeted origami (CX3CR1-targeted) was incubated with different serum concentrations for 5 minutes and analyzed using DLS. **c)** This shows the difference between origami and origami with a serum protein corona, as measured using Fluorescence Correlation Spectroscopy (FCS). Untargeted and immune cell-targeted origami are labeled with Atto594.

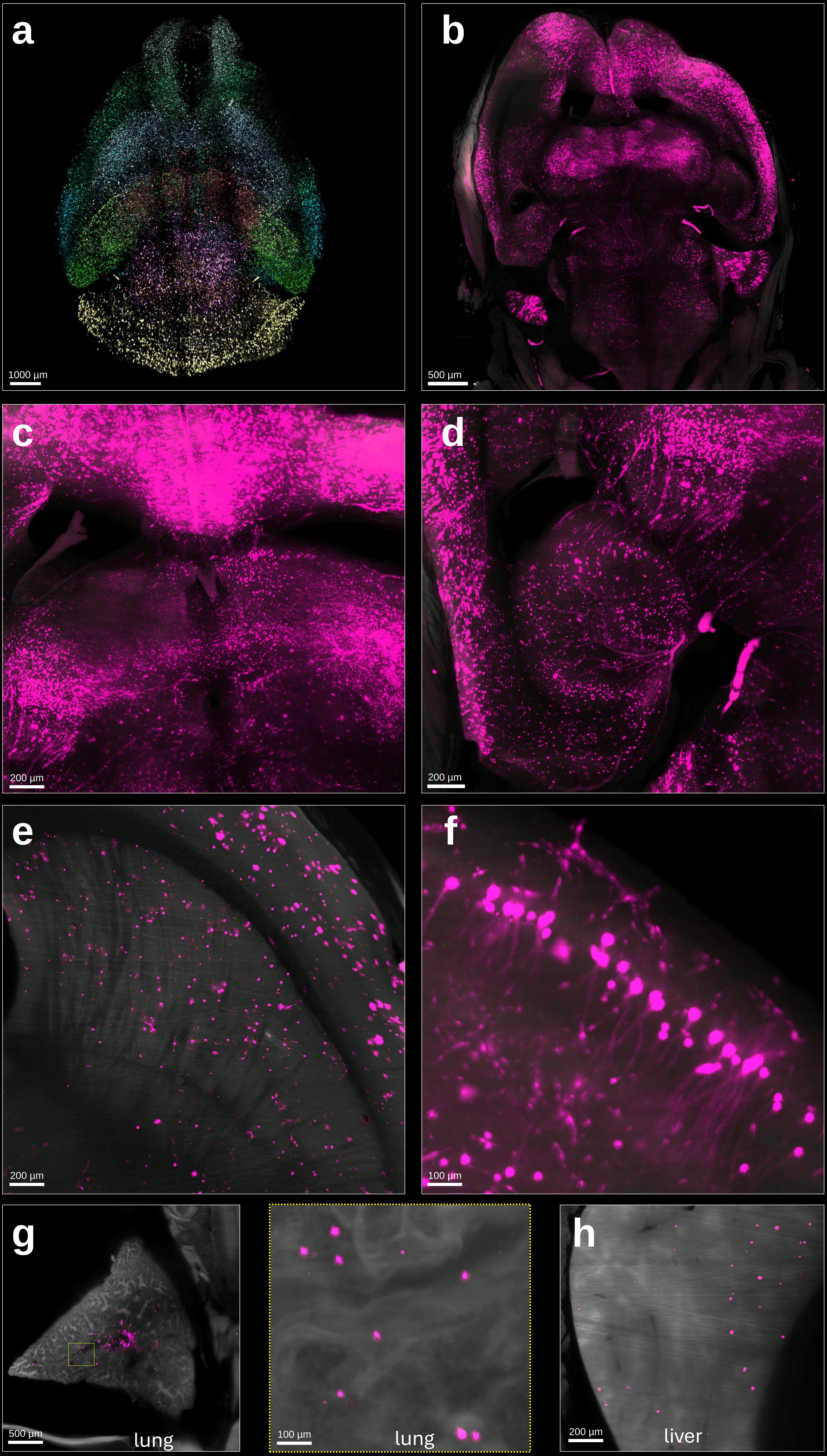

Supplementary Fig. 19: PHPeB AAV distribution in whole-body.

Targeted cells in the brain **(a, segmented, b: original),** sub-brain regions **(c-f)**, as well as in the lung **(g)** and liver **(h)**. Color-coding per Allen Brain Atlas CCF3 regions.

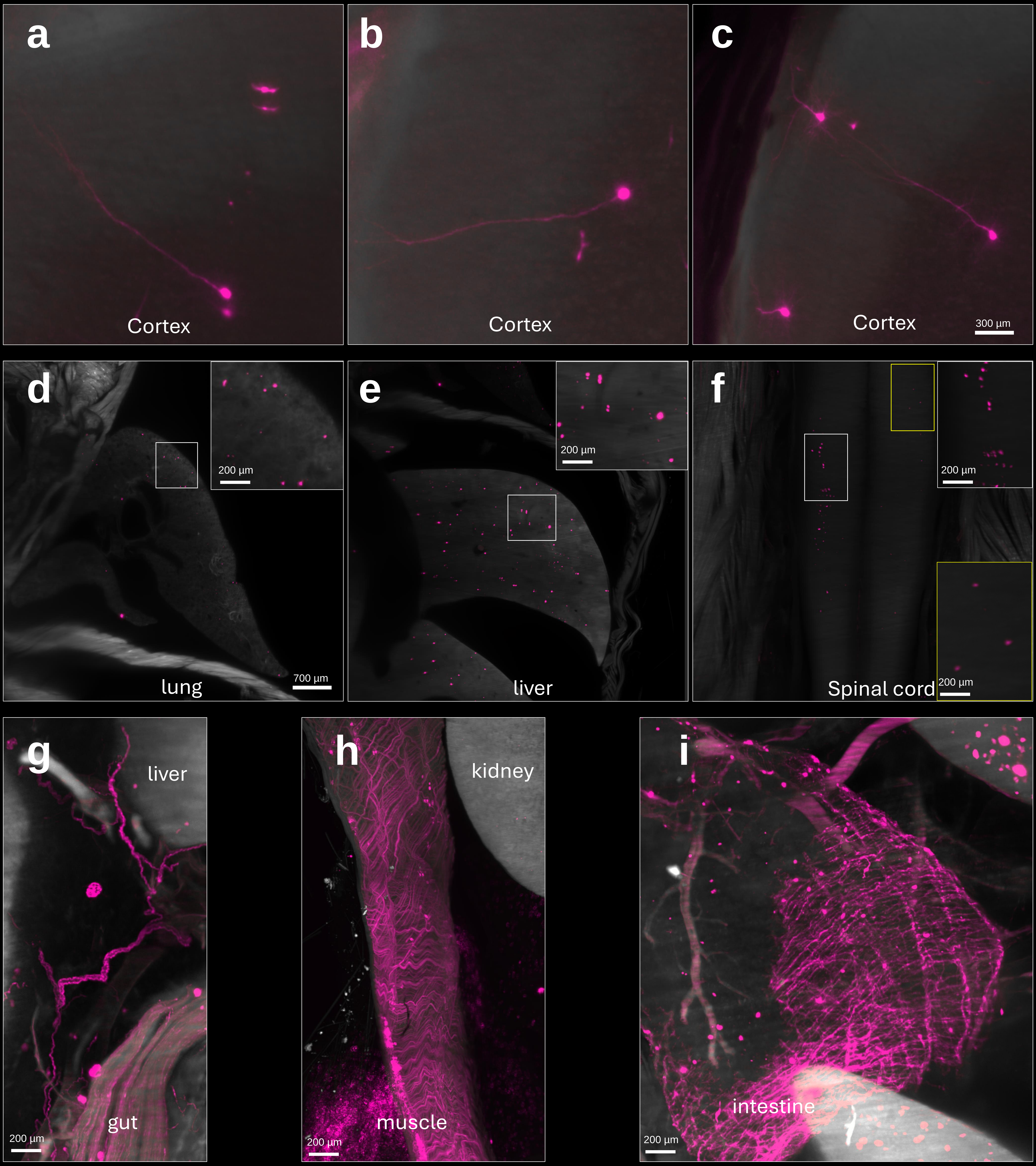

Supplementary Fig. 20: Retro AAV targeting.

The distribution in the cortex **(a-c)**, lung **(d)**, liver **(e)**, spinal cord **(f)**, area near the liver **(g)**, muscle **(h)**, and intestine **(i)**.

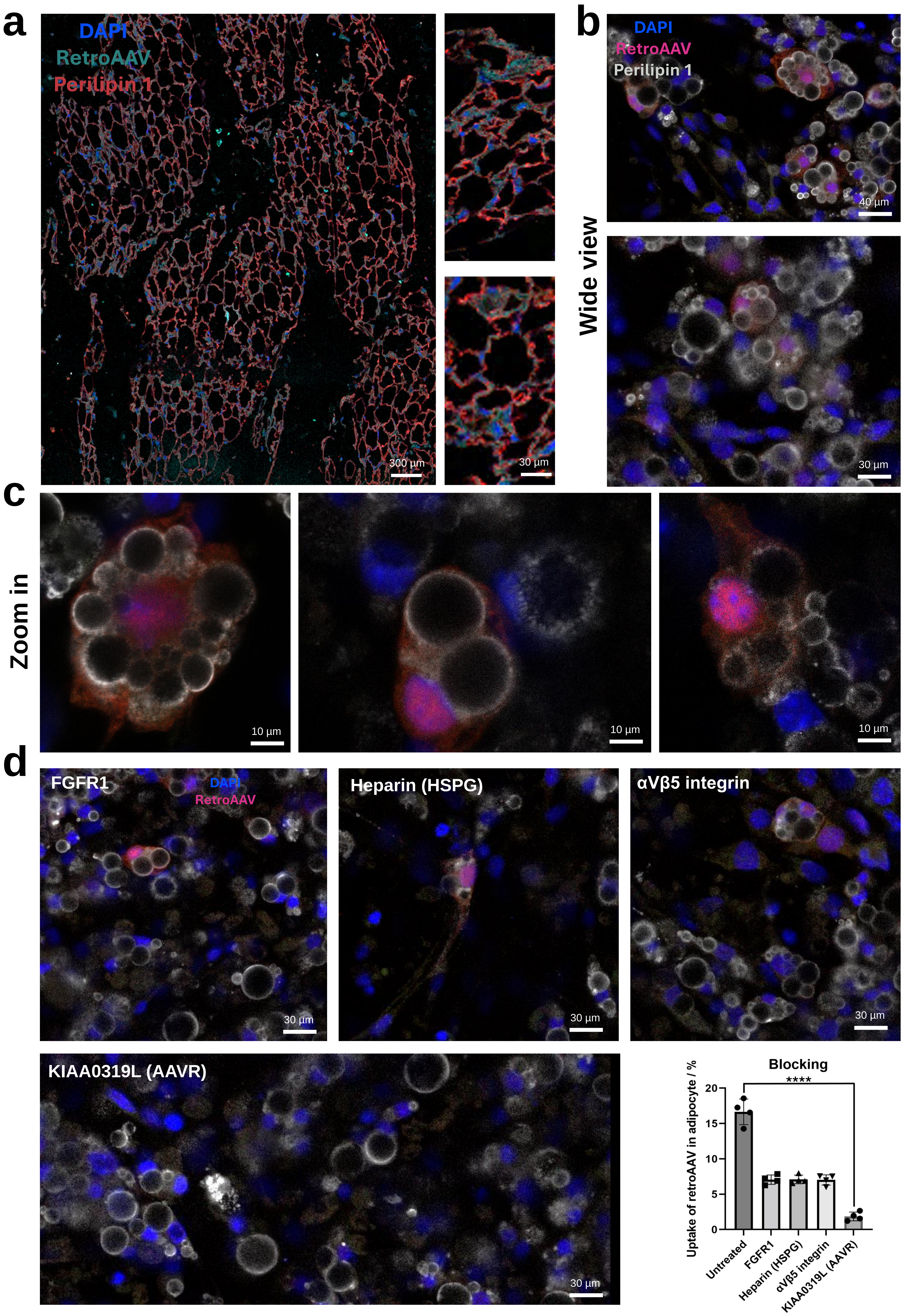

Supplementary Fig. 21: Mechanism of RetroAAV targeting adipose tissue via the AAVR receptor.

a) Histology confirms that RetroAAV targets adipocytes. After intramuscular (i.m.) injection of RetroAAV, the brown fat was extracted from cleared mice for histological analysis. Perilipin 1-labeled adipocytes are shown in red, RetroAAV-EGFP is shown in green, and DAPI-labeled nuclei are shown in blue. **(b-d)** *In vitro* demonstration of the targeting of RetroAAV to adipocytes. **b,c)** RetroAAV uptake by adipocytes. The upper panel shows a wide view, and the lower panel shows a zoomed-in view of RetroAAV within adipocytes. **d)** Different blocking agents for inhibition assays. An excess amount of FGFR1, heparin, αVβ5 integrin, and KIAA0319L was used to block RetroAAV entry into adipocytes. Perilipin 1-labeled adipocytes are shown in gray, RetroAAV-EGFP is shown in magenta, and DAPI-labeled nuclei are shown in blue. n = 4 different cultures per condition, mean ± s.d. ****: $P < 0.0001$. (one-way analysis of variance).

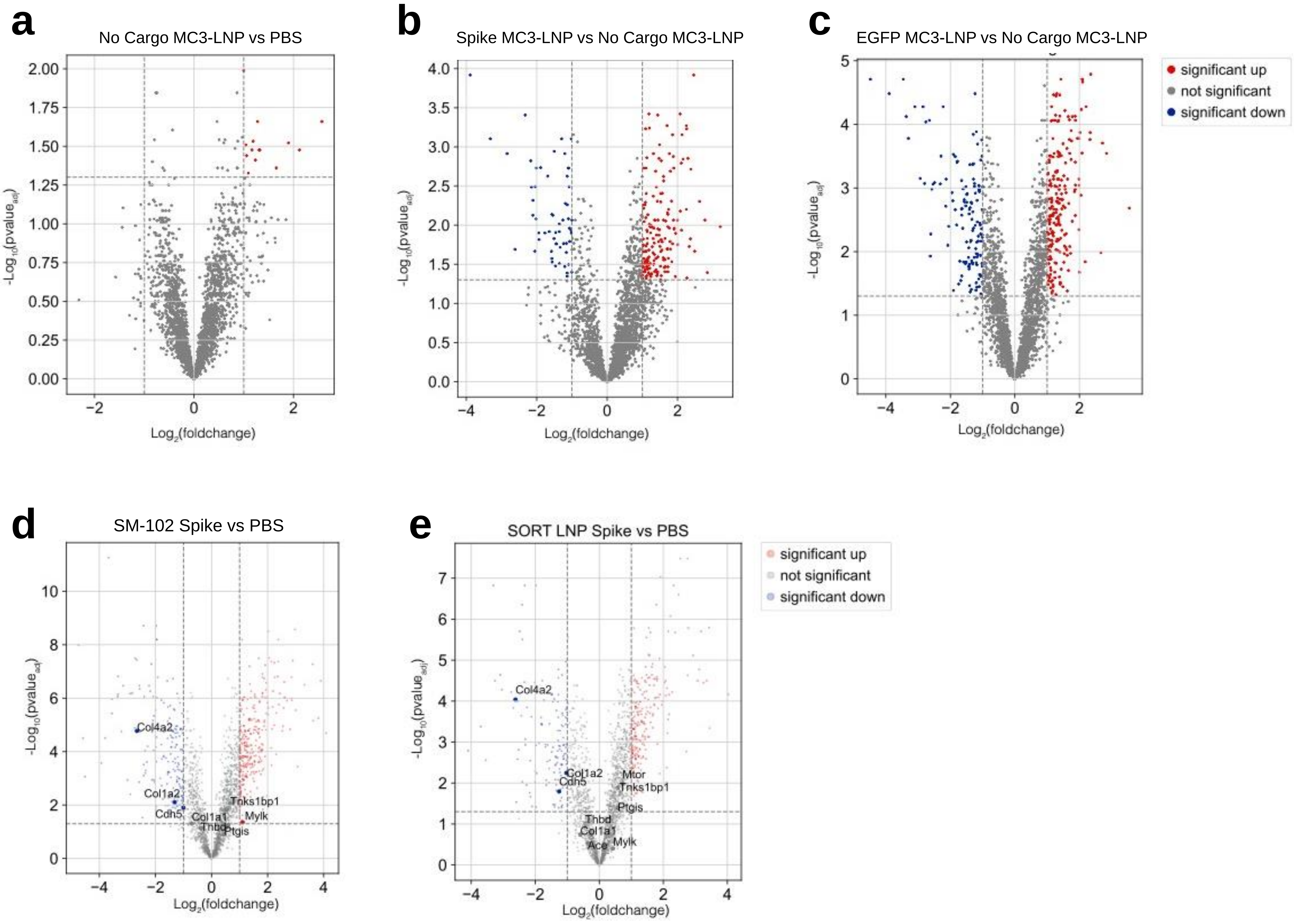

Supplementary Fig. 22: Proteomics analysis of diverse LNP formulations on mouse heart tissue.
No cargo MC3-LNP vs. PBS **(a)**, Spike MC3-LNP vs. No cargo MC3-LNP **(b)**, EGFP MC3-LNP vs. No cargo MC3-LNP **(c)**, SM-102 Spike vs. PBS **(d)**, and SORT Spike vs. PBS **(e)**. Log2(FC)=1, p-value<0.5.

Supplementary Table. 1: Prediction accuracy of lymph nodes using SCP-Nano

	**SCP-Nano**			
	TP	FP	FN	**Instance Dice**
lymph nodes	663	146	197	**0.7945**

Supplementary Table. 2: Protein markers for different directional analyses (inflammation and vascular changes)

Vascular Markers	Cd34, Vwf, Col6a3, Col15a1, Col4a1, Col3a1, Col4a2, Col1a1, Col18a1, Col1a2, Col6a2, Col6a1, Pcolce, Col11a2, Col14a1, Col6a6, Colgalt1, Col4a4

Supplementary Table. 3: Prediction accuracy of AAV traced neuron cell bodies using SCP-Nano

	**SCP-Nano (retrained on AAV traced neuron cell bodies data)**			
	TP	FP	FN	**Instance Dice**
**all**	342	132	37	**0.8019**

Supplementary Video Legends

Supplementary Video 1

Distribution of lipid nanoparticles (in magenta) in the lung following a 0.0005 mg/kg intranasal injection after 6 hours.

Supplementary Video 2

Distribution of EGFP protein expression (in green) in the heart 72 hours after i.m. injection of 0.0005 mg/kg LNP-EGFP mRNA.

Supplementary Video 3

Distribution of LNP-spike mRNA (in magenta) in the heart 6 hours after i.m. injection of a dose of 0.0005 mg/kg.

Supplementary Video 4

Distribution of DNA origami (in red) at the cell level throughout the entire mouse body, especially in the spleen, liver, and lung.

Supplementary Video 5

Distribution of GFP expressed from PHP.eB AAV-delivered DNA two weeks after intravenous injection in different brain regions. Scale bar 1 mm. Color-coding per Allen Brain Atlas CCF3 regions.

Supplementary Video 6

3D visualization of the distribution of GFP expressed from RetroAAV-delivered DNA throughout the entire mouse body at cell-level resolution, with GFP in cyan and the background in grey.
