# Supplementary material for: Nanocarrier imaging at single-cell resolution across entire mouse bodies with deep learning
Source: Nat Biotechnol. 2025 Jan 14;43(12):2009–22. doi: 10.1038/s41587-024-02528-1 (PMC12700832; doi:10.1038/s41587-024-02528-1)
Supplement: Supplementary file 2 — Reporting Summary [file 41587_2024_2528_MOESM2_ESM.pdf]

nature portfolio

Corresponding author(s): Dr. Ali Erturk

Last updated by author(s): Oct 11, 2024

Reporting Summary

Nature Portfolio wishes to improve the reproducibility of the work that we publish. This form provides structure for consistency and transparency in reporting. For further information on Nature Portfolio policies, see our Editorial Policies and the Editorial Policy Checklist.

Statistics

For all statistical analyses, confirm that the following items are present in the figure legend, table legend, main text, or Methods section.

n/a	Confirmed	
☐	☒	The exact sample size (*n*) for each experimental group/condition, given as a discrete number and unit of measurement
☐	☒	A statement on whether measurements were taken from distinct samples or whether the same sample was measured repeatedly
☐	☒	The statistical test(s) used AND whether they are one- or two-sided<br>*Only common tests should be described solely by name; describe more complex techniques in the Methods section.*
☐	☒	A description of all covariates tested
☐	☒	A description of any assumptions or corrections, such as tests of normality and adjustment for multiple comparisons
☐	☒	A full description of the statistical parameters including central tendency (e.g. means) or other basic estimates (e.g. regression coefficient) AND variation (e.g. standard deviation) or associated estimates of uncertainty (e.g. confidence intervals)
☐	☒	For null hypothesis testing, the test statistic (e.g. *F*, *t*, *r*) with confidence intervals, effect sizes, degrees of freedom and *P* value noted<br>*Give P values as exact values whenever suitable.*
☒	☐	For Bayesian analysis, information on the choice of priors and Markov chain Monte Carlo settings
☒	☐	For hierarchical and complex designs, identification of the appropriate level for tests and full reporting of outcomes
☒	☐	Estimates of effect sizes (e.g. Cohen's *d*, Pearson's *r*), indicating how they were calculated

Our web collection on statistics for biologists contains articles on many of the points above.

Software and code

Policy information about availability of computer code

Data collection	Data collection was performed using: ImSpector (Version 7.3.2, MiltenyiBioTec GmbH), Imaris (Version 9.6.0 Bitplane AG), and syGlass(version 1.7.2)
Data analysis	Data analysis was performed using: Fiji (version 1.51) , GraphpadPrism (version 8).<br>Proteomics data were analyzed using Scanpy (v. 1.10.2) and AnnData (v. 0.10.8) in Python 3.10, along with the Scikit-learn package (v. 1.5.1).<br>The missing values were input using KNNImputer from sklearn package (v. 1.5.1).<br>Proteomics data undergo batch correction using ComBat from scib tools: https://github.com/theislab/scib (v. 1.1.3).<br>SCP-Nano pipeline codes are available here: https://github.com/erturklab/SCP-Nano.

For manuscripts utilizing custom algorithms or software that are central to the research but not yet described in published literature, software must be made available to editors and reviewers. We strongly encourage code deposition in a community repository (e.g. GitHub). See the Nature Portfolio guidelines for submitting code & software for further information.

Data

Policy information about availability of data

All manuscripts must include a data availability statement. This statement should provide the following information, where applicable:

- Accession codes, unique identifiers, or web links for publicly available datasets
- A description of any restrictions on data availability
- For clinical datasets or third party data, please ensure that the statement adheres to our policy

All data that support the findings of this study are available from the corresponding author upon reasonable request. The SCP-Nano pipeline code is shared here: https://github.com/erturklab/SCP-Nano. Proteomics data undergo batch correction using ComBat from scib tools: https://github.com/theislab/scib (v. 1.1.3). The proteomics data was uploaded to the PRIDE partner repository with the dataset identifier PXD056871, accessible at http://proteomecentral.proteomexchange.org. Source data are provided with this paper.

Research involving human participants, their data, or biological material

Policy information about studies with human participants or human data. See also policy information about sex, gender (identity/presentation), and sexual orientation and race, ethnicity and racism.

Reporting on sex and gender	N/A
Reporting on race, ethnicity, or other socially relevant groupings	N/A
Population characteristics	N/A
Recruitment	N/A
Ethics oversight	N/A

Note that full information on the approval of the study protocol must also be provided in the manuscript.

Field-specific reporting

Please select the one below that is the best fit for your research. If you are not sure, read the appropriate sections before making your selection.

☒ Life sciences ☐ Behavioural & social sciences ☐ Ecological, evolutionary & environmental sciences

For a reference copy of the document with all sections, see nature.com/documents/nr-reporting-summary-flat.pdf

Life sciences study design

All studies must disclose on these points even when the disclosure is negative.

Sample size	If not specifically stated in the figure legends, the n number is 3.
Data exclusions	No collected data were excluded.
Replication	The experiments were repeated at least three times.
Randomization	Four-week-old mixed-gender mice were randomly used in the experiments.
Blinding	Blinding was not performed in this study because the primary outcomes were measured using objective, quantitative methods (e.g., biochemical assays, deep-learning based imaging analyses) that minimize the potential for subjective bias. Additionally, the experimental design focused on standardized procedures and automated data collection, reducing the risk of observer bias. Given these objective endpoints, blinding was deemed unnecessary for maintaining the integrity and reliability of the results.

Reporting for specific materials, systems and methods

We require information from authors about some types of materials, experimental systems and methods used in many studies. Here, indicate whether each material, system or method listed is relevant to your study. If you are not sure if a list item applies to your research, read the appropriate section before selecting a response.

Materials & experimental systems

n/a	Involved in the study
☐	☒ Antibodies
☒	☐ Eukaryotic cell lines
☒	☐ Palaeontology and archaeology
☐	☒ Animals and other organisms
☒	☐ Clinical data
☒	☐ Dual use research of concern
☒	☐ Plants

Methods

n/a	Involved in the study
☒	☐ ChIP-seq
☒	☐ Flow cytometry
☒	☐ MRI-based neuroimaging

Antibodies

Antibodies used	Perilipin 1 (Cell Signaling, Cat# 3470S) Podocalyxin (R and D Systems, Cat# MAB1556), Alpha smooth muscle actin SMA (Abcam, Cat# ab5694), Cx3cr1 Antibody (Thermofisher, Cat# PA1-29224), Rat Anti-CD45 (BD Biosciences, Cat# 14-0451-82), SARS-CoV-2 Spike Antibody (GeneTex, Cat# GTX135356), Alexa Fluor 568-conjugated anti-GFP signal-enhancing nanobodies (Chromotek, Cat# gb2AF568-50), Alexa Fluor 647-conjugated anti-GFP signal-enhancing nanobodies (Chromotek, Cat# gb2AF647)
Validation	Perilipin 1 (Cell Signaling, Cat# 3470S) was validated for adipocytes in vitro.<br>Podocalyxin (R&D Systems, Cat# MAB1556), alpha smooth muscle actin (SMA; Abcam, Cat# ab5694), and rat anti-CD45 (BD Biosciences, Cat# 14-0451-82) were validated for heart tissue.<br>Cx3cr1 antibody (Thermo Fisher, Cat# PA1-29224), SARS-CoV-2 spike antibody (GeneTex, Cat# GTX135356), Alexa Fluor 568-conjugated anti-GFP signal-enhancing nanobodies (Chromotek, Cat# gb2AF568-50), and Alexa Fluor 647-conjugated anti-GFP signal-enhancing nanobodies (Chromotek, Cat# gb2AF647) were validated for whole-mouse-body immunostaining.

Animals and other research organisms

Policy information about studies involving animals; ARRIVE guidelines recommended for reporting animal research, and Sex and Gender in Research

Laboratory animals	4-week-old wildtype mice were purchased from Charles River Laboratories. The animals were housed under a 12/12-hour light/dark cycle and had random access to food and water. The temperature was maintained at 18–23°C and humidity was at 40–60%.
Wild animals	This study does not involve wild animals.
Reporting on sex	Wild-type mixed-gender mice (C57BL/6J) were used randomly.
Field-collected samples	This study does not involve field-collected samples.
Ethics oversight	The animal experiments were conducted according to institutional guidelines of the Klinikum der Universität München/Ludwig Maximilian University of Munich and after approval of the Ethical Review Board of the Government of Upper Bavaria (Regierung von Oberbayern, Munich, Germany) and under the European Directive 2010/63/EU for animal research.

Note that full information on the approval of the study protocol must also be provided in the manuscript.

Plants

Seed stocks	*Report on the source of all seed stocks or other plant material used. If applicable, state the seed stock centre and catalogue number. If plant specimens were collected from the field, describe the collection location, date and sampling procedures.*
Novel plant genotypes	*Describe the methods by which all novel plant genotypes were produced. This includes those generated by transgenic approaches, gene editing, chemical/radiation-based mutagenesis and hybridization. For transgenic lines, describe the transformation method, the number of independent lines analyzed and the generation upon which experiments were performed. For gene-edited lines, describe the editor used, the endogenous sequence targeted for editing, the targeting guide RNA sequence (if applicable) and how the editor was applied.*
Authentication	*Describe any authentication procedures for each seed stock used or novel genotype generated. Describe any experiments used to assess the effect of a mutation and, where applicable, how potential secondary effects (e.g. second site T-DNA insertions, mosiacism, off-target gene editing) were examined.*
